# Improving Energy and Molecular Properties by Convergence of the One‐Particle Reduced Density Matrix in Variational Quantum Eigensolvers (VQE)

**DOI:** 10.1002/jcc.70289

**Published:** 2026-01-05

**Authors:** Amanda Marques de Lima, Erico Souza Teixeira, Eivson Darlivam Rodrigues de Aguiar Silva, Ricardo Luiz Longo

**Affiliations:** ^1^ Departamento de Química Fundamental Universidade Federal de Pernambuco Recife Brazil; ^2^ Centro de Excelência em Computação Quântica Venturus Campinas Brazil

**Keywords:** density matrix, molecular properties, quantum computing, variational quantum eigensolver

## Abstract

The variational quantum eigensolver (VQE) is a relevant method for simulating molecular systems on near‐term quantum computers. While its primary application is the estimation of ground‐state energies, VQE also produces the one‐particle reduced density matrix (1‐RDM), from which other relevant molecular properties can be obtained. The accuracy of these properties depends on the reliability and convergence of the 1‐RDM, which is not guaranteed by energy‐only optimization. Thus, two new algorithms were introduced: VQE* that incorporates the RMSD of consecutive 1‐RDM as a convergence criterion and VQE‐LD that modifies the cost function by adding to the energy a term involving the RMSD of 1‐RDM weighted by a proper factor. These algorithms were tested for protonated methane, CH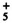, at equilibrium and four dissociation geometries, with the k‐UpCCGSD (4,4)‐ and GateFabric (2,2)‐active space *ansätze*. For k‐UpCCGSD, whose energies are already close to CASCI(4,4), improvements were mainly observed in density‐dependent properties such as electron density, dipole moments, and Mulliken charges. For GateFabric, which initially displayed larger energy deviations, both approaches significantly improved the energy accuracy and the quality of the 1‐RDM. Overall, our findings show that the convergence of the energy and of the 1‐RDM provides a simple yet effective strategy to improve the accuracy of energies and molecular properties in variational quantum algorithms.

## Graphical abstract

Two new strategies that include the convergence of the one‐particle reduced density matrix (1‐RDM) were introduced in Variational Quantum Eigensolvers (VQE). Improved energy and, most importantly, density‐based properties (dipole moments, electron density) were obtained. So, simple strategies for more reliable molecular predictions with near‐term quantum computers were devised.

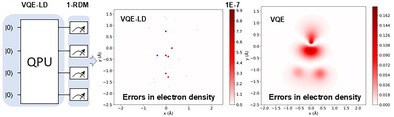

## 1 Introduction

Quantum mechanics is fundamental for the accurate description of molecular properties such as relative energies, charge densities, and dissociation profiles [1]. These properties can be obtained from the solution of the Schrödinger equation. However, the exact solution of this equation for systems with many electrons is computationally challenging, which motivated the development of approximate methods [1].

Despite their success, these methods often come with a computational cost that grows exponentially with the size and complexity of the system [2]. This limitation is evident in systems with strong electron correlation, such as those containing transition metals. For example, the accurate description of the FeMo cofactor can require petabytes of memory and impractical execution times [3, 4]. Despite significant progress in classical computing, further advancements are increasingly limited by fundamental physical constraints, including saturation of transistor scaling, memory bandwidth, storage capacity bottlenecks, and limited scalability of parallel algorithms due to Amdahl's Law and interconnect latency [5].

These limitations are particularly evident in the simulation of quantum systems. As Feynman argued [6], “nature itself is governed by quantum mechanics, suggesting that more natural and efficient simulations should be carried out on devices that also obey quantum laws.” In this scenario, quantum computers have emerged as a promising alternative to overcome such limitations [7]. Although these improvements are still theoretical and depend on advances in *hardware* and algorithms, these computers offer a compact representation of complex systems. For example, while the classical simulation of the caffeine molecule would require approximately $10^{48}$ bits, a quantum computer could represent the same system with approximately 160 quantum bits or qubits [8].

In this context, one of the most promising applications of quantum computing is the simulation of chemical systems. This has the potential to transform areas such as medicine, materials science, catalysis, and nanotechnology [9, 10]. To this end, several quantum algorithms have been proposed, with Quantum Phase Estimation [11], or QPE, being one of the best known. However, applying QPE to chemically relevant systems typically requires fault‐tolerant quantum computers with millions of logical qubits and deep quantum circuits, which makes it impractical for current devices of the NISQ (Noisy Intermediate‐Scale Quantum) era [12, 13].

Alternatively, the Variational Quantum Eigensolver (VQE) stands out as a hybrid algorithm that combines measurements on quantum computers with optimizations on classical computers [14, 15]. VQE allows the estimation of the ground‐state energy of molecules with greater practical feasibility than QPE, having been successfully tested in systems such as protocatechuic acid [16], benzene [17], and diazene and hydrogen chains with up to 12 atoms [18].

Despite these advances, VQE still faces challenges in accuracy due to the dependence of the results on the quantum circuit (sequence of logic gates applied to each qubit) and the search space accessed during optimization [19], and improvements to this method are being sought [10]. An important direction for improving VQE involves moving beyond energy as the sole metric of performance. Although the primary goal of VQE is to minimize the total energy, this does not ensure that the one‐particle reduced density matrix (1‐RDM) also converges satisfactorily. This could limit the appropriate description of properties more sensitive to fluctuations of the 1‐RDM, such as forces, dipole moments, electron density topology, and population analyses [20, 21]. Therefore, enhancing the estimation of the 1‐RDM within VQE is essential for obtaining more reliable predictions of molecular properties, especially in systems with strong electron correlation.

Current developments, related with the 1‐RDM, of VQE have been in two directions: using properties derived from the 1‐RDM as validation metrics or exploring alternative strategies for optimizing the 1‐RDM. In the first category, Skogh et al. [22] proposed validating VQE by comparing experimentally accessible properties derived from 1‐RDM, such as electron density topologies and atomic partial charges, rather than comparing to 1‐RDM directly. Similarly, Le et al. [23] employed properties, such as dipole moments, also derived from 1‐RDM, as benchmarks for assessing the quality of the VQE results.

The second category of developments focuses on incorporating density matrices into iterative refinement schemes. For example, Tilly et al. [24] integrated VQE into a complete active‐space self‐consistent field (CASSCF) framework, where the active‐space wave function and molecular orbitals are iteratively optimized using the two‐particle reduced density matrix (2‐RDM) obtained from VQE. This 2‐RDM is used to classically update the orbital rotation matrix, allowing variational mixing of orbitals. The molecular Hamiltonian is then reconstructed in the new orbital basis, and VQE is rerun until convergence is achieved. This method yielded accurate dipole moments and Fermi liquid parameters.

Lew et al. [25] proposed a conceptually different approach in which the VQE generates a wavefunction and extracts the 1‐RDM. This 1‐RDM is diagonalized to yield natural orbitals and occupation numbers, which are then inserted into a selected natural orbital functional (NOF), an approximate energy functional of the 1‐RDM, to compute the energy of the system without directly measuring the Hamiltonian. Because the Hamiltonian and orbitals are not updated during optimization, the accuracy and efficiency of this approach are determined by the quality of the chosen functional.

Inspired by these strategies, we propose two new VQE approaches: (i) VQE* that incorporates the root‐mean‐square deviation (RMSD) between consecutive 1‐RDM as a convergence criterion and (ii) VQE‐LD that modifies the cost function by adding to the energy a weighted term involving the RMSD of 1‐RDM. The goal of both methods is to converge the energy and the 1‐RDM sequentially or simultaneously. It is noteworthy that these approaches preserve the original VQE structure, avoiding repeated Hamiltonian reconstructions or complete diagonalization of the 1‐RDM, and thus provide a simpler alternative to the optimizations discussed above. Furthermore, we compare the molecular properties obtained with both methods with those of standard VQE, highlighting that minimizing the energy alone does not necessarily guarantee optimal performance.

The next section presents some relevant concepts of VQE and 1‐RDM, detailing how molecular properties are obtained from this matrix. It also presents the details of VQE* and VQE‐LD, along with the techniques employed in this work, both on classical computers and quantum simulators. It is followed by the presentation and discussions of the results obtained for the CH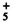 molecule, highlighting the benefits of these proposed approaches. The final section summarizes the main findings and outlines possible directions for future research.

## 2 Methods

This section describes the computational methods employed to investigate the impact of optimizing the 1‐RDM within the VQE framework. The standard VQE algorithm, including its mathematical formulation, circuit design, and *ansatz* used for quantum state preparation, is initially outlined. Next, the formalism of the 1‐RDM and its connection to some chemical properties, such as electron density, dipole moment, and Mulliken charges, is presented. Two new VQE approaches are introduced: VQE* that sequentially minimizes the energy and converges the 1‐RDM and VQE‐LD that minimizes a cost function, which incorporates the energy and its gradient, as well as the 1‐RDM and its gradient. The systems of interest are presented along with their simulation parameters and the corresponding active space used.

### 2.1 Variational Quantum Eigensolver

In the VQE algorithm, the cost function is given by the expectation value of the Hamiltonian of the system, Ĥ, evaluated over a parameterized trial wavefunction, in accordance with the Schrödinger equation [15]:

(1) $$Ĥ|\Psi_{0}(\theta )\rangle =E_{0}|\Psi_{0}(\theta )\rangle ,$$

where $E_{0}$ is the ground‐state energy, and $\left|\Psi_{0}(\theta )\right\rangle$ is a variational parameterized quantum state that approximates the eigenvector corresponding to the lowest eigenvalue $E_{0}$. The goal of VQE is to adjust the variational parameters θ to minimize the expectation value of Ĥ, providing an accurate estimate of the ground‐state energy. This procedure is based on the variational principle, ensuring that the estimated energy will always be greater than or equal to the exact energy of the ground‐state [26].

**FIGURE 1:**
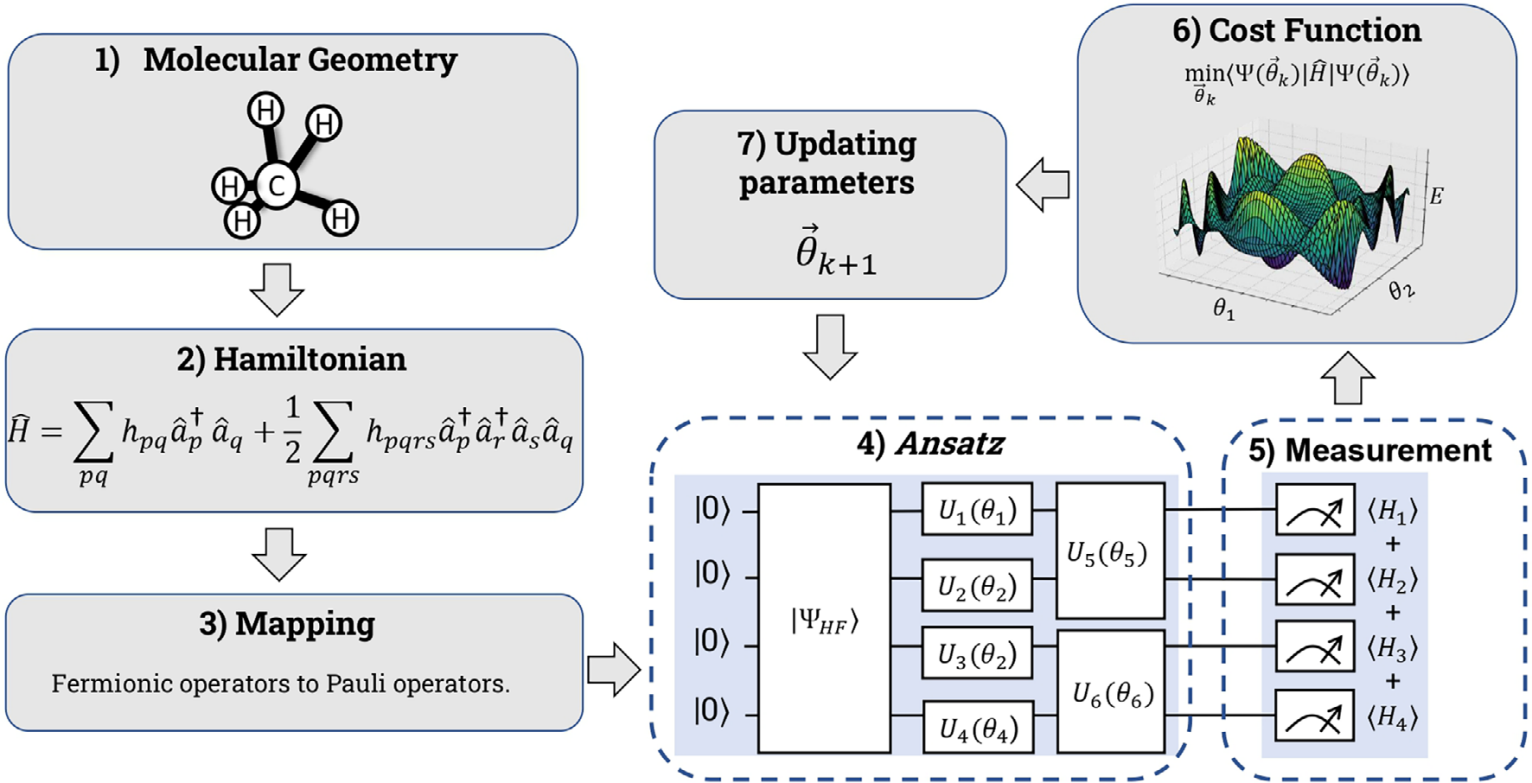
Representation of the flow of the VQE algorithm. The steps in gray (1, 2, 3, 6, and 7) refer to the processes executed on the classical computer.

The main steps of the VQE algorithm are illustrated in Figure 1. Initially, for a given molecular geometry, the Ĥ of the molecule is obtained in the second quantized form. This Hamiltonian is expressed in the fermionic basis and, therefore, cannot be directly implemented in qubit‐based quantum devices. To make it compatible, it is necessary to transform it to the Pauli operator basis [19, 27].

This transformation can be performed through different mappings, such as the Jordan–Wigner (JW), Bravyi‐Kitaev, and Parity schemes. Among them, the JW mapping is widely used because it offers a direct correspondence between spin‐orbitals and qubits. In this case, occupied and unoccupied spin‐orbitals are mapped to the qubit states |1⟩ and |0⟩, respectively [28]. The JW mapping transforms the creation, $a_{p}^{†}$ and annihilation, $a_{q}$, operators into:

(2) $$\begin{array}{c} a_{p}^{†}=\frac{1}{2}\left(X_{p}-iY_{p}\right)\overset{p-1}{\underset{k=0}{\prod }}Z_{k},\\ a_{q}=\frac{1}{2}\left(X_{q}+iY_{q}\right)\overset{q-1}{\underset{k=0}{\prod }}Z_{k}. \end{array}$$

with *X*, *Y*, and *Z* being the Pauli operators. As a result, the molecular fermionic Hamiltonian is mapped onto a qubit Hamiltonian which is expressed in terms of Pauli strings [19]:

(3) $$Ĥ=\underset{j}{\sum }\alpha_{j}P_{j},$$

where $\alpha_{j}$ are real coefficients and $P_{j}$ represent products of Pauli operators acting on the qubits.

Once the Hamiltonian Ĥ is expressed in the Pauli basis, the next step is to choose a parameterized *ansatz*, together with the corresponding initial state and variational parameters θ. The *ansatz* is a trial quantum circuit designed to approximate the ground state of Ĥ by preparing a family of wavefunctions that depend smoothly on the parameters θ. A common choice for the initial state is the Hartree–Fock (HF) wavefunction [19]. If the variational parameters θ are initialized to zero, the prepared state reduces exactly to the HF reference. In contrast, initializing θ with random values produces a state that deviates from the HF configuration [19].

The quantum computer (or simulator) estimates the expectation value of the Hamiltonian Ĥ for the parametrized state |Ψ(θ)⟩ by sampling measurement outcomes of each Pauli operator $P_{j}$ [19]. A classical optimizer then iteratively updates the *ansatz* parameters to minimize the estimated energy, using feedback from the quantum device at each step until convergence [19, 29]. The optimized parameters yield an approximation to the ground‐state energy and wavefunction that can subsequently be used to compute molecular properties.

### 2.2 Reduced Density Matrix and Molecular Properties

In the second quantization formalism, the expectation value of any operator $\hat{\Omega }$ can be expressed as [30]:

(4) $$\langle \Psi |\hat{\Omega }|\Psi \rangle =\underset{pq}{\sum }D_{pq}\Omega_{pq}+\frac{1}{2}\underset{pqrs}{\sum }d_{pqrs}\Omega_{pqrs}+\Omega_{0},$$

where $\Omega_{pq}$ and $\Omega_{pqrs}$ represent the one‐electron and two‐electron integrals, respectively, and $\Omega_{0}$ is a constant term. The quantities $D_{pq}$ and $d_{pqrs}$ correspond to the one‐ and two‐particle reduced density matrices (1‐RDM and 2‐RDM), which encapsulate information about the electronic structure of the system.

The 1‐RDM provides insight into the distribution and occupation of molecular orbitals and is defined as [30, 31]:

(5) $$D_{pq}=\langle \Psi |a_{p}^{†}a_{q}|\Psi \rangle ,$$

where $a_{p}^{†}$ and $a_{q}$ are the creation and annihilation operators, respectively. This matrix encodes the occupation of molecular orbitals through its diagonal elements and the quantum coherence between orbitals through its off‐diagonal elements.

This 1‐RDM is expressed in the molecular orbital (MO) basis, but can be converted to the atomic orbital basis by the relationship between MOs and AOs (atomic orbitals) given by:

(6) $$\phi_{p}(r)=\underset{\nu }{\sum }C_{\nu p}\varphi_{\nu }(r),$$

where $C_{\nu p}$ are the molecular coefficients that express the molecular orbitals $\phi_{p}(r)$ in terms of the atomic basis functions $\varphi_{\nu }(r)$. The 1‐RDM can be converted from the MO to the AO basis through the transformation:

(7) $$\gamma_{\mu \nu }=\underset{pq}{\sum }C_{\mu p}D_{pq}C_{\nu q}^{*},$$

where C is the coefficient matrix that maps MO to AO, which can be obtained from the HF method using, for instance, PySCF [32].

Several molecular properties can be obtained from 1‐RDM, such as the electron density [33], the molecular electrostatic potential [34], the dipole moment [35], as well as the Mulliken charges and other population analyses [35]. The methods for obtaining these properties from 1‐RDM will be discussed in more detail below.

#### 2.2.1 Electronic Density

The electron density describes the distribution of electrons in a molecule in space [36]. It represents the probability of finding an electron at a given element of volume and is a central concept in quantum chemistry. Electron density is not only used to visualize the shape of molecules and chemical bonds but also serves as the foundation for interpreting chemical structure, reactivity, and bonding patterns [37]. High‐density regions typically occur near atomic nuclei and between bonded atoms, reflecting areas of strong electron localization.

The electron density can be obtained from the 1‐RDM using the following expression [33, 35]:

(8) $$\rho (r)=\;\underset{pq}{\sum }D_{pq}\phi_{p}(r)\phi_{q}^{*}(r),$$

where $D_{pq}$ are the elements of the 1‐RDM, and $\phi_{p}(r)$, and $\phi_{q}(r)$ represent the basis functions associated with the molecular orbitals.

However, to calculate the electron density, the 1‐RDM must first be expressed in the AO basis. This transformation is necessary because the basis functions used by PySCF [32], the framework employed to evaluate these properties, are defined in the AO representation, while the 1‐RDM obtained from both VQE and HF are originally represented in the MO basis.

With the 1‐RDM expressed in the atomic basis, the electron density in the atomic basis could be calculated according to [35]:

(9) $$\rho (r)=\underset{\mu \nu }{\sum }\gamma_{\mu \nu }\varphi_{\mu }(r)\varphi_{\nu }^{*}(r),$$

where $\varphi_{\mu }(r)$ and $\varphi_{\nu }^{*}(r)$ are the atomic basis functions.

A robust theoretical approach for analyzing chemical structure and reactivity based on electron density is the Quantum Theory of Atoms‐in‐Molecules (QTAIM) [38]. This approach performs a topological analysis of ρ(r) to identify critical points that represent, for instance, regions of large electron concentration around nuclei or between pairs of neighboring nuclei [38].

The topological characterization is based on the identification of critical points (CPs), defined as the locations where the gradient of the electron density vanishes [22]. These points are classified according to the local curvature of the density, which is described by the Hessian matrix of ρ(r), as shown in Equation (10) [38]. Diagonalizing this matrix yields eigenvalues that determine whether a critical point corresponds to a maximum, minimum, or saddle point.

(10) $$\nabla^{2}\rho (r)=\left[\begin{array}{ccc} \frac{\partial^{2}\rho }{\partial x^{2}} & \frac{\partial^{2}\rho }{\partial x\partial y} & \frac{\partial^{2}\rho }{\partial x\partial z}\\ \frac{\partial^{2}\rho }{\partial y\partial x} & \frac{\partial^{2}\rho }{\partial y^{2}} & \frac{\partial^{2}\rho }{\partial y\partial z}\\ \frac{\partial^{2}\rho }{\partial z\partial x} & \frac{\partial^{2}\rho }{\partial z\partial y} & \frac{\partial^{2}\rho }{\partial z^{2}} \end{array}\right]$$

Among the main types of critical points, the bond critical points (BCPs) are particularly important. These are located between two nuclei and are indicative of the presence of a chemical bond [38]. They are characterized by two negative and one positive eigenvalue of the Hessian, reflecting two directions of electron density decrease and one of increase. Nuclear critical points (NCPs), found at the positions of atomic nuclei, correspond to local maxima in electron density and are typically associated with positive eigenvalues, indicating high electron concentration around the nucleus [38]. Other types of critical points, such as ring and cage critical points, also exist, but will not be discussed in this work.

#### 2.2.2 Electrostatic Potential

The electrostatic potential represents the electric potential generated by the nuclei charges and electron density in a molecule [39]. It describes how a test positive charge would experience force at any point in space around the molecule. This potential plays a central role in understanding chemical reactivity, non‐covalent interactions, and molecular recognition, as regions of high or low potential influence how molecules interact with each other [34].

The molecular electrostatic potential generated at a position r in the vicinity of a molecular system is given by [34]:

(11) $$V(\text{r})=\;\overset{N}{\underset{A}{\sum }}\frac{Z_{A}}{\left|\text{r}-{\text{R}}_{A}\right|}-\int \frac{\rho ({\text{r}}^{\prime })}{\left|\text{r}-{\text{r}}^{\prime }\right|}d{\text{r}}^{\prime },$$

where $Z_{A}$ is the charge of the nucleus A, $\rho ({\text{r}}^{\prime })$ is the electron density at r', obtained from the 1‐RDM, while $\left|\text{r}-{\text{r}}^{\prime }\right|$ is the distance between the point r, whose potential is being calculated, and the point ${\text{r}}^{\prime }$, where the charge $\rho ({\text{r}}^{\prime })$ is located.

#### 2.2.3 Dipole Moment

The dipole moment is a vector quantity that measures the separation of positive and negative charge distributions within a molecule [40]. Molecules with large dipole moments tend to interact more strongly with electric fields and polar solvents [40].

The molecular dipole moment can be calculated from the 1‐RDM using the following expression [35]:

(12) $$\mu_{x}=-\underset{\mu }{\sum }\underset{\nu }{\sum }\gamma_{\mu \nu }(\nu |x|\mu )+\underset{A}{\sum }Z_{A}X_{A},$$

where $\mu_{x}$ is the *x*‐component of the total dipole moment vector of the molecule, $\gamma_{\mu \nu }$ are the elements of the 1‐RDM, (ν|x|μ) represents the integral of the position operator in the x direction, $Z_{A}$ is the charge of the nucleus A and $X_{A}$ its x‐coordinate. The y‐ and z‐components have analogous forms, allowing the construction of the complete dipole vector.

#### 2.2.4 Mulliken Population Analysis

Population analyses aim at separating the electron density of a molecule into specific atomic contributions [41]. Partial atomic charges serve as an important rationalization of reactivity and are relevant for understanding non‐covalent interactions [41].

Mulliken analysis allows estimating the electron populations and partial charges on atoms from the 1‐RDM and the overlap matrix, defined as $S_{\nu \mu }=\langle \varphi_{\nu }|\varphi_{\mu }\rangle$, where $\varphi_{\nu }$ and $\varphi_{\mu }$ are basis functions centered on different atoms [35, 41].

The electron population associated with an atom A is given by [35]:

(13) $$P_{A}=\underset{\mu \in A}{\sum }\underset{\nu }{\sum }\gamma_{\mu \nu }S_{\nu \mu },$$

where μ∈A indicates that the μ orbital is centered on the A atom. The Mulliken charge on the A atom is then obtained as [35]:

(14) $$q_{A}=Z_{A}-P_{A},$$

where $Z_{A}$ is the atomic number of the atom A.

### 2.3 1‐RDM With Active Space

Modeling the electronic structure with quantum computing is challenging because the qubit requirements scale linearly with the size of the basis set, an asymptotic advantage over classical computing, but the number of qubits needed for realistic systems still exceeds current hardware capacities [19]. A common solution is to use small basis sets and to select an active space as a subset of MOs [19], such as in the CASSCF method [42]. A well‐constructed, effective Hamiltonian of a small active space can nearly capture the effects of the entire orbital space [43]. This space typically includes the most relevant MOs, so that MOs that do not belong to the active space remain frozen [44].

**FIGURE 2:**
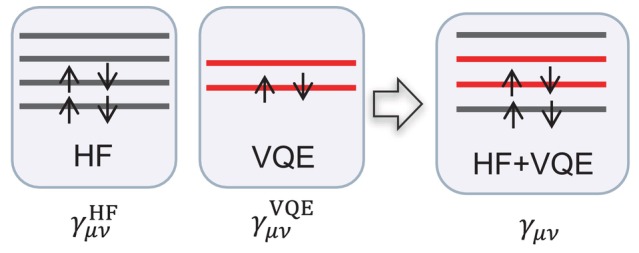
Construction of 1‐RDM using active space. The active and frozen orbitals are generated by VQE and HF, respectively.

When an active space is employed in a VQE calculation, the resulting 1‐RDM contains elements involving only the active orbitals. As shown in the Figure 2, to reconstruct the full molecular 1‐RDM, including both active and frozen orbitals, it is necessary to merge the VQE output ($\gamma_{\mu \nu }^{\text{VQE}}$) with information from a HF density matrix ($\gamma_{\mu \nu }^{\text{HF}}$). This is achieved by replacing the elements of the HF 1‐RDM corresponding to the active orbitals with those computed via VQE, while retaining the HF values for the frozen orbitals (Equation 15). In this hybrid approach, the electron correlation within the active space is captured by VQE, whereas the frozen orbitals remain described by the HF wavefunction.

(15) $$\gamma_{\mu \nu }=\left\{\begin{array}{cc} \gamma_{\mu \nu }^{\text{VQE}},\; & \text{if}\;\mu ,\nu \in \text{active space}\\ \gamma_{\mu \nu }^{\text{HF}},\; & \text{otherwise} \end{array}\right.$$

### 2.4 Evaluation of 1‐RDM

The 1‐RDM in the MO basis, denoted as $D_{pq}$ (Equation 5), can be constructed from the expectation values of the fermionic creation and annihilation operators mapped to qubits, through the Jordan–Wigner transformation (Equation 2), with the optimized parameters via VQE.

The diagonal elements of the 1‐RDM are obtained by measuring the expectation value of the Z operator on each qubit [18]:

(16) $$\langle a_{p}^{†}a_{p}\rangle =\frac{I-\langle Z_{p}\rangle }{2},$$

where I is the identity operator.

In contrast, the off‐diagonal elements (p≠q) encode quantum coherences between orbitals, reflecting superpositions and relative phases [18]. In the Hartree–Fock approximation, the 1‐RDM is strictly diagonal in the canonical MO basis due to its single‐determinant nature, but post‐Hartree–Fock methods and quantum computing approaches generally yield non‐negligible off‐diagonal terms. Because the operators $a_{p}^{†}a_{q}$ (p≠q) are not Hermitian, their expectation values may be complex. However, only Hermitian operators are directly measurable on quantum devices. To circumvent this limitation, the expectation values of the Hermitian combinations of these excitation operators are measured. These Hermitian combinations will correspond to the real and imaginary components of the 1‐RDM elements as follows [18, 45]:

(17) $$\begin{array}{c} \begin{array}{cc} \text{Re}[D_{pq}] & \;=\frac{1}{2}\;\left\langle \Psi (\theta )\;|\;a_{p}^{†}a_{q}+a_{q}^{†}a_{p}\;|\;\Psi (\theta )\right\rangle \\ & \;=\frac{1}{4}\;\left\langle \Psi (\theta )\;|\;X_{p}X_{q}+Y_{p}Y_{q}\;|\;\Psi (\theta )\right\rangle , \end{array} \end{array}$$

(18) $$\begin{array}{c} \begin{array}{cc} \text{Im}[D_{pq}] & \;=\frac{1}{2i}\;\left\langle \Psi (\theta )\;|\;a_{p}^{†}a_{q}-a_{q}^{†}a_{p}\;|\;\Psi (\theta )\right\rangle \\ & \;=\frac{1}{4}\;\left\langle \Psi (\theta )\;|\;X_{p}Y_{q}-Y_{p}X_{q}\;|\;\Psi (\theta )\right\rangle . \end{array} \end{array}$$

When the *ansatz* is restricted to real‐valued rotations, such as circuits implementing SO(N) transformations, off‐diagonal elements are guaranteed to be real, and the imaginary part vanishes [18]. However, more sophisticated *ansätze*, including those employed in this work, involve parameterized excitations and general qubit rotations that generate complex amplitudes. In such cases, the Jordan–Wigner transformation produces qubit operators with explicit complex factors, and the off‐diagonal elements of the 1‐RDM can acquire nonzero imaginary components. Measuring both parts separately is therefore essential to recover a Hermitian and physically meaningful 1‐RDM.

The complete 1‐RDM can be reconstructed by evaluating only the independent elements in the upper triangular part of the matrix (p≤q). This procedure naturally includes the diagonal terms (p=q), which correspond to orbital occupations, as well as the real and imaginary parts of the off‐diagonal coherences. These elements are

(19) $$D_{pq}=\text{Re}[D_{pq}]+i\;\text{Im}[D_{pq}],\;p\leq q,$$

and the matrix is completed by Hermitian conjugation,

(20) $$D_{qp}=D_{pq}^{*},\;p<q.$$

This assembly avoids redundant measurements and explicitly preserves the Hermitian character of the 1‐RDM.

### 2.5 Optimization of 1‐RDM

**FIGURE 3:**
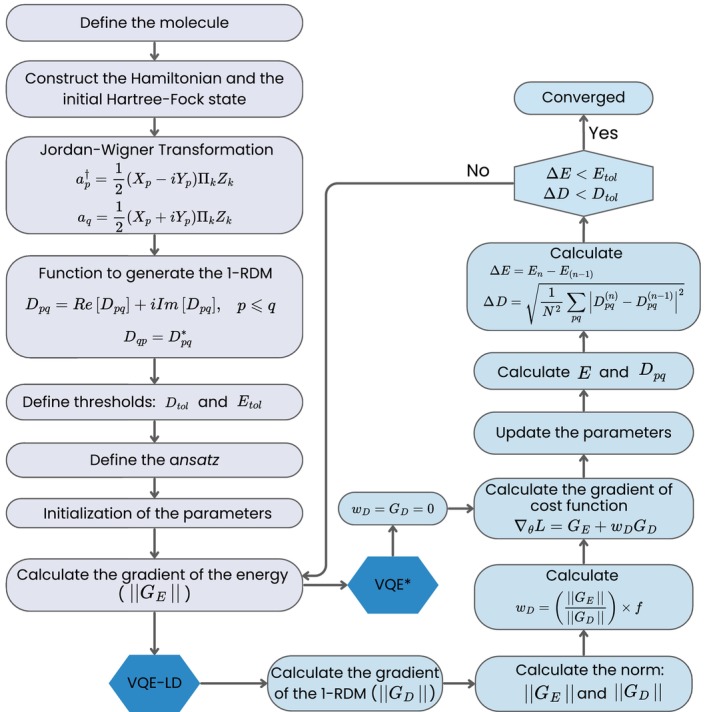
Flowchart for obtaining the convergence of the energy and of the 1‐RDM through VQE, using the VQE* and VQE‐LD methods proposed in this work. In the first step, $G_{D}=0$.

In this work, two modifications to the traditional VQE were proposed and are illustrated in the flowchart presented in Figure 3. The procedure begins as in the standard VQE, namely, providing the molecular geometry and the basis set, followed by the construction of the second‐quantized Hamiltonian in a defined active space. The fermionic creation and annihilation operators are mapped to qubit operators using the JW transformation. Then, the convergence thresholds of the method are established: $E_{\text{tol}}$ for the energy and $D_{\text{tol}}$ for the 1‐RDM, followed by the selection of the *ansatz* and its initial parameters. Subsequently, the energy gradient with respect to these parameters is calculated.

The first algorithm, denoted as VQE*, consists of a simplified version that preserves the structure of the standard VQE, but introduces an additional convergence criterion based on the RMSD [46] between the 1‐RDM of the current iteration ($D_{pq}^{\left(n\right)}$) and that of the previous iteration ($D_{pq}^{\left(n-1\right)}$), defined as:

(21) $$\Delta D=\sqrt{\frac{1}{N^{2}}\underset{pq}{\sum }{\left|D_{pq}^{\left(n\right)}-D_{pq}^{\left(n-1\right)}\right|}^{2}},$$

where N represents the dimension of the 1‐RDM (number of active orbitals). In this way, convergence depends not only on energy minimization but also on the stability of the 1‐RDM.

The second approach, named VQE‐LD (VQE with Loss function associated with reduced Density matrix), further expands this modification. In this case, the cost function is modified by adding to the energy a term involving the RMSD of 1‐RDM (ΔD), weighted properly by $w_{D}$. The energy and ΔD as well as the gradient of the energy ($G_{E}$) and the gradient of the RMSD of 1‐RDM ($G_{D}$) have different units and very different magnitudes, with the norm of $G_{E}$ usually being significantly smaller than that of $G_{D}$. This prevents the direct combination of these quantities in the cost function. Thus, the norms of both gradients were calculated, and a weighting factor $w_{D}$ was introduced as the ratio of the norm of $G_{E}$ to the norm of $G_{D}$:

(22) $$w_{D}=\left(\frac{\left\|G_{E}\right\|}{\left\|G_{D}\right\|}\right)\times f,$$

The factor f is a learning rate parameter, which can be adjusted to accelerate the convergence of the VQE algorithm.

With this modification, the cost function of the VQE‐LD algorithm is defined as a weighted sum of the energy and the RMSD of the 1‐RDM between consecutive iterations:

(23) $$L(\theta )=E(\theta )+w_{D}\;\Delta D(\theta )$$

where E(θ) is the expectation value of the Hamiltonian.

During optimization, the gradient of this cost function with respect to the variational parameters is computed as

(24) $$\nabla_{\theta }L=G_{E}+w_{D}\;G_{D},$$

where $G_{E}=\nabla_{\theta }E(\theta )$ and $G_{D}=\nabla_{\theta }\Delta D(\theta )$ are obtained using analytical differentiation. The term $G_{D}$ represents the gradient of the current 1‐RDM‐based loss ΔD(θ) with respect to the circuit parameters, while $D^{\left(n-1\right)}$ from the previous iteration is treated as a constant reference. This formulation ensures that both components, energy minimization and 1‐RDM fluctuations, contribute consistently to the parameter updates and that all gradients have the same dimension (units).

In both algorithms, after calculating the loss function, the parameters of the quantum circuit are updated by a classical optimizer, and the new energy and $D_{pq}$ are defined. After that, the variation in energy and RMSD of the 1‐RDM are calculated to determine if the VQE has converged.

### 2.6 System of Interest and Computational Details

To apply these ideas, the dissociation profile of the protonated methane CH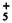 was analyzed, a molecule whose structure has been widely discussed in the literature. In its equilibrium geometry, CH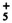 exhibits the character of a complex between CH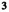 and H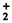 (CH
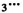
H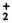), evolving to CH
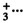
H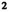 during the dissociation [47]. It is one of the simplest and, at the same time, most enigmatic carbocations, acting as a highly reactive intermediate in processes involving hydrocarbons [48]. Furthermore, CH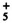 has relevance in several contexts, such as organic reaction mechanisms [49], astrochemistry [50], planetary atmospheres [51], and mass spectrometry [52].

The dissociation coordinate of CH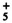 (Figure S1 and Table S1) was defined as the distance between the carbon and the center of mass of the leaving H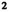, which is 1.3 Å in the equilibrium region. Dissociation geometries with distances, R, of 1.4, 1.6, 1.8, and 2.1 Å were obtained. These distances were chosen to provide a comprehensive view of the dissociation curve of this molecule, capturing both the binding and dissociation regimes.

Geometry optimization and energies were obtained using CISD (configuration interaction singles and doubles) [30] and STO‐3G basis set [53, 54]. These calculations were performed using Gaussian 09 [55].

The STO‐3G basis set was selected for the quantum simulator calculations due to its low computational cost, making it suitable for the VQE algorithm by minimizing the number of qubits required to model molecules. All simulations were implemented with the PennyLane library [56].

In preliminary tests, we used the UCCSD [57], k‐UpCCGSD [58], GRSD [59], GateFabric [60], and the Entanglement‐variational Hardware‐efficient [59] *ansätze*, with active spaces formed by 4, 8, 12, and 16 qubits. From these tests, the k‐UpCCGSD (with k=1) demonstrated excellent energy accuracy compared to the CASCI(4,4) values when applied to the (4,4)‐active space (i.e., 4 electrons within 4 MOs), corresponding to 8 qubits. For this reason, both k‐UpCCGSD and (4,4)‐active space were chosen.

The k‐UpCCGSD is an extension of UCCSD, designed to reduce circuit depth by two key modifications [58]. First, excitations are generalized to allow transitions between any orbitals without distinguishing occupied and virtual. Second, double excitations adopt a pair coupled‐cluster (pCC) structure, involving only linked‐clusters, thus reducing the number of terms in the circuit [58]. The *ansatz* is applied in k repeated layers, each with a distinct set of excitation operators. This layered structure increases expressiveness, with circuit cost scaling linearly with system size and the prefactor k, allowing a balance between depth and accuracy [19].

Although the energy of the previous test showed good agreement with CASCI(4,4), a scenario in which the energy presents significant errors was also evaluated to test the robustness of the method in obtaining molecular properties. For this purpose, GateFabric was used, which showed significant deviations in the CH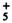 energy for R=1.3 and 1.4 Å, when applied to the (2,2)‐active space (2 electrons within 2 MOs) with 4 qubits. Therefore, this second scenario (GateFabric with reduced active space) was also included in the analysis for these two geometries.

GateFabric uses Givens rotations to perform single and double excitations, in addition to adding fermionic SWAP gates [60]. Furthermore, this circuit performs double excitations only between neighboring qubits. Its construction combines features of a hardware‐efficient *ansätze* (HEA), due to local connectivity and the repetitive gate structure, with aspects typical of chemically inspired *ansätze*, explicitly preserving the particle number and spin symmetries, a property generally absent in conventional HEA [60, 61].

In the VQE, VQE* and VQE‐LD approaches, the convergence criteria were set to $1\times 10^{-6}$ for both $E_{\text{tol}}$ and $D_{\text{tol}}$. The optimization process employed the stochastic gradient method (SGD), with a learning rate equal to 0.4. For VQE‐LD, the learning rate parameter, f, affects the number of optimization steps and can modify the energy. For k‐UpCCGSD, values from f=0.05 to 1.0 were tested, while for GateFabric, these values were varied from 0.1 to 1.0, based on preliminary tests.

For all tests, the initial parameters chosen for the VQE were zero, meaning the calculation is based on a HF reference. After VQE optimization, the following molecular properties were determined: dipole moment, electron density, electrostatic potential, and Mulliken charges and populations. Topological analysis of this density was performed with the Critic2 program [62], after generating the density file in “.cube” format using the PySCF Cubegen module, with a resolution of 0.10.

Preliminary energy comparisons were conducted using CISD and CASCI (Complete Active Space Configuration Interaction) [63] within the (2,2)‐ and (4,4)‐active space, as well as with Full Configuration Interaction (FCI). Because the VQE‐based methods employed relatively small active spaces, a direct comparison with FCI would be unbalanced, as it includes all possible excitations within the entire orbital space. Thus, the comparisons were performed with respect to CISD, CASCI(4,4), and FCI, but the discussions will be focused on CASCI(4,4), because it seems more consistent with the VQE approaches. The comparisons with CISD and FCI are provided in the Supporting Information.

Both VQE* and VQE‐LD approaches yielded more negative energies, in some instances, compared to CISD and CASCI(2,2), indicating better variational performance of the former methods. For a more consistent and insightful comparison, the CASCI(4,4) was employed as reference in the discussion, as it systematically includes all excitations within a moderate active space similar to the VQE framework, which treats only a subset of orbitals explicitly. The CASCI, CISD, and FCI results were obtained using PySCF [32].

## 3 Results and Discussion

In this section, the results of the optimization and acquisition of the molecular properties of the CH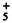 dissociation geometries using k‐UpCCGSD in the (4,4)‐ and GateFabric (2,2)‐active space will be presented, using three algorithms: (i) the traditional VQE; (ii) VQE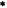; and (iii) VQE‐LD. The results of these approaches were compared with those obtained using CASCI(4,4) method. First, the results will be presented in detail for the k‐UpCCGSD‐(4,4) *ansatz*, then they are complemented by the GateFabric‐(2,2) results.

### 3.1 Improving Molecular Properties of the k‐UpCCGSD *Ansatz* With Converged 1‐RDM

The calculations with the k‐UpCCGSD‐(4,4) *ansatz* aimed at improving the molecular properties are presented and discussed. For the VQE‐LD approach, the rate f needs to be optimized, and it was found that increasing f led to an increase in the number of steps to reach convergence (Table S3). Notice that only for R=1.8 and 2.1 Å did this increase in f clearly affect the energy values. However, these energy changes occur after the fourth decimal place, and depending on f, many steps are needed to achieve this precision. Therefore, the criterion adopted was to choose the value of f that balanced the number of steps and energy error, but in such a way that the 1‐RDM continued to affect the cost function L. Thus, for R=1.8 Å, f=0.1 was used, while for the other geometries, f=0.05 was chosen.

#### 3.1.1 Energy and Density Matrix Analysis

The VQE* and VQE‐LD approaches showed smaller energy differences relative to CASCI(4,4) than did traditional VQE (Table 1). However, these errors remain at the fifth or sixth decimal place, indicating that both the proposed methods and the traditional VQE remain within chemical precision (error $<1.6\times 10^{-3}$ $E_{\text{h}}$) in all geometries evaluated. Despite the slight energy improvement, traditional VQE requires fewer optimization steps than the two proposed methods; among them, VQE* converges in fewer steps than VQE‐LD. Notice that the accuracy of all three methods decreases for distances larger or equal to 1.8 Å, i.e., in the bond‐breaking region, where changes in the dominant determinants of the wavefunction modify the convergence behavior of the 1‐RDM and in the optimal learning‐rate parameter f for both VQE* and VQE‐LD.

**TABLE 1:** CASCI(4,4) reference energies and energy errors ($E_{\text{method}}-E_{\mathrm{CASCI}(4,4)}$) (in $E_{\text{h}}$) for the VQE, VQE*, and VQE‐LD methods, computed with the k‐UpCCGSD *ansatz* in a (4,4)‐active space for CH structures at different R.

R (Å)	CASCI(4,4) ($E_{\text{h}}$)	Error ($\times 10^{-6}$ $E_{\text{h}}$)			ΔD ($\times 10^{-6}$)			Steps		
		VQE	VQE*	VQE‐LD	VQE	VQE*	VQE‐LD	VQE	VQE*	VQE‐LD
1.3	−39.91925976	3.99	3.30	3.30	64.0	0.92	0.93	7	20	23
1.4	−39.91888589	2.99	2.54	2.55	44.8	0.75	0.87	7	19	22
1.6	−39.91533506	4.38	3.78	3.78	10.7	0.91	0.96	8	15	19
1.8	−39.91719786	95.72	85.17	84.85	50.5	0.96	0.99	17	40	41
2.1	−39.90743429	10.13	8.09	8.05	69.6	0.95	0.95	14	39	44

*Note:* Also shown are ΔD and the number of optimization steps.

Energy comparisons with CISD in the (4,4)‐active space and with FCI are available in Table S2 (Supporting Information). At some distances, VQE* and VQE‐LD are superior to CISD, resulting in more negative energy, but they are less precise than FCI, as expected by the *ansätze* (Table S2).

As shown in Table 1, the ΔD obtained with VQE* and VQE‐LD is about two orders of magnitude smaller than with traditional VQE (from $10^{-5}$ to $10^{-7}$), indicating a substantially more accurate 1‐RDM. This is significant because 1‐RDM accuracy underpins density‐sensitive molecular properties, particularly in strongly correlated regimes [21]. The 1‐RDMs obtained with CISD, CASCI, VQE, VQE*, and VQE‐LD in (4,4)‐active space and using the k‐UpCCGSD *ansatz* are presented in Figure S2. Figure S3 compares the CASCI(4,4) 1‐RDM with those from VQE, VQE*, and VQE‐LD. The differences are similar, but slightly larger, than those obtained with CISD (Figure S4). In summary, energy gains are marginal since VQE is already close to CASCI(4,4), but the pronounced reduction in ΔD suggests better performance for density‐dependent observables, at the cost of additional optimization steps.

#### 3.1.2 Dipole Moment

The errors in the dipole moments, Δμ, relative to the CASCI(4,4) reference values, are smaller with VQE* and VQE‐LD compared to traditional VQE (Table 2). The same trend was observed with CISD in the same active space (Table S4). This improvement is particularly noticeable at R=1.8 Å, where the Δμ for traditional VQE is $-1.40\times 10^{-2}$ D. In contrast, with VQE* and VQE‐LD, the error decreases to $-3.20\times 10^{-3}$ and $-2.10\times 10^{-3}$ D, representing an improvement of 4.38 and 6.67 times, respectively. These results show that optimizing the 1‐RDM or incorporating the RMSD gradient between consecutive 1‐RDM into the cost function significantly enhances the description of the dipole moment of CH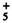 at equilibrium and dissociating structures.

**TABLE 2:** Total dipole moment (μ) and corresponding error $\left(\Delta \mu =\mu_{\text{method}}-\mu_{\mathrm{CASCI}(4,4)}\right)$, in Debye (D), for CH geometries at different R, computed with the k‐UpCCGSD *ansatz* in a (4,4)‐active space.

R (Å)	μ (D)				Δμ ($\times 10^{-3}$ D)		
	CASCI(4,4)	VQE	VQE*	VQE‐LD	VQE	VQE*	VQE‐LD
1.3	1.9208	1.9252	1.9209	1.9209	4.40	0.10	0.10
1.4	1.8150	1.8178	1.8150	1.8145	2.80	0.00	−0.50
1.6	1.1189	1.1196	1.1189	1.1190	7.00	0.00	0.10
1.8	0.5812	0.5672	0.5780	0.5791	−14.00	−3.20	−2.10
2.1	0.6764	0.6913	0.6766	0.6768	14.90	0.23	0.43

#### 3.1.3 Electron Density

**FIGURE 4:**
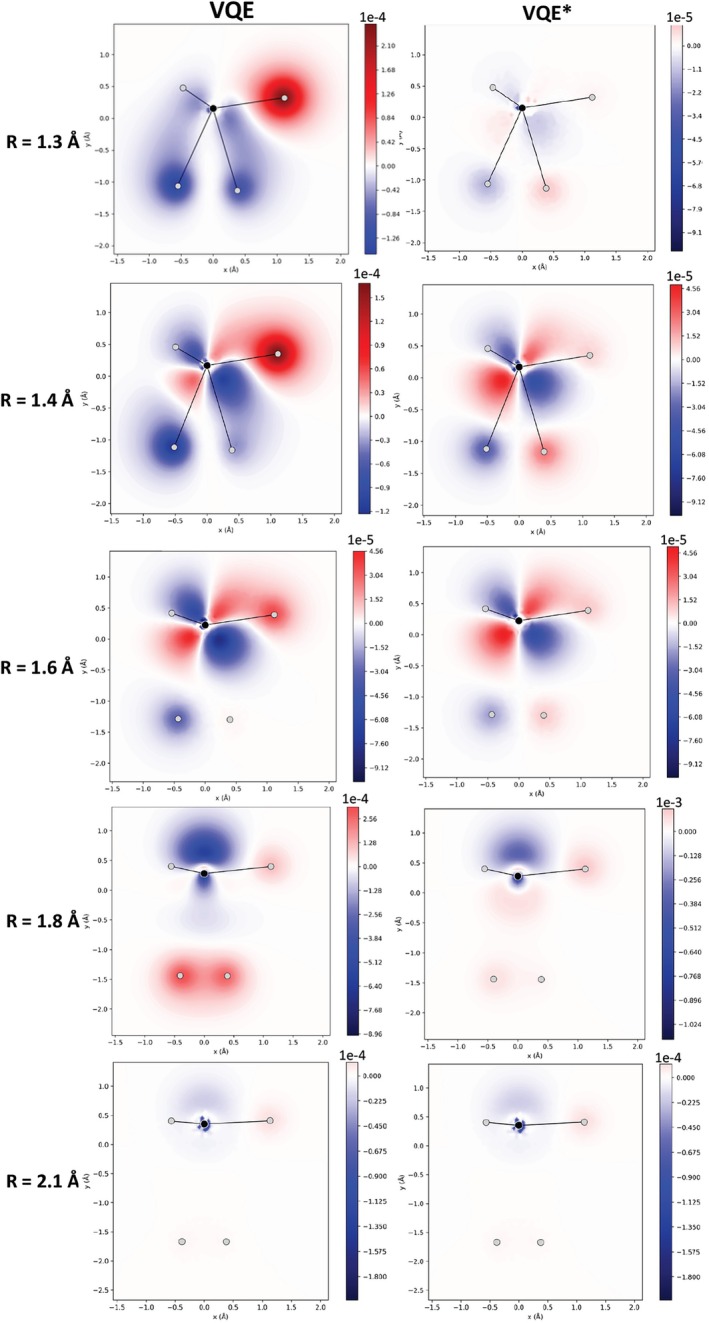
Difference in electron density (in $e/a_{0}^{3}$) along the x and y axes for dissociating CH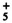 geometries computed with VQE, VQE*, and VQE‐LD, using the k‐UpCCGSD *ansatz* in a (4,4)‐active space, relative to CASCI(4,4). In the molecular layout, H3 and H4 lie at the bottom of the structure; H5 is at the upper left; H6 is positioned behind H5 (hence not visible in the figure); and H2 is located at the upper right.

Figure 4 illustrates the difference in the electron density calculated with VQE and VQE* relative to the CASCI(4,4) density for the different CH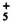 dissociation geometries. The results obtained with VQE‐LD are presented in Figure S6 and are practically the same as those obtained with VQE*. The differences between the three methods in relation to CASCI(4,4) follow the same trends as those obtained with CISD(4,4) (Figure S7).

Notice that all three methods yield similar overall patterns for the electron density differences, suggesting that the general form of the wavefunction was approximately converged before 1‐RDM optimization. However, for R=1.3 and 1.4 Å, the one order of magnitude smaller scale of the VQE* and VQE‐LD errors compared to standard VQE indicates a remarkable improvement in the quantitative description of the electron density. Only for R=1.6 and 2.1 Å, the three methods present practically the same electron density. Thus, it is observed that even when the ground‐state energy approaches the CASCI value, the electron density quality can be significantly improved through 1‐RDM optimization. This can be important for properties that are either local in nature or highly sensitive to the electron distribution.

These findings are supported and visualized by the topological analysis of the electron density—namely, the values of ρ and its Laplacian ($\nabla^{2}\rho$) at nuclear critical points (NCPs) and bond critical points (BCPs), reported in Table 3. Figure S5 shows the positions of the BCPs during dissociation, indicating that even at highly stretched geometries (1.8 and 2.1 Å), critical points persist between the carbon and distant hydrogens. VQE, VQE*, and VQE‐LD recover the same NCPs and BCPs as CASCI(4,4), demonstrating that the electron density topology is preserved despite the use of approximate variational *ansätze*. This highlights the ability of VQE to capture weak intermolecular interactions, a task often challenging for approximate electronic structure methods.

**TABLE 3:** Differences between the results of the electron density (in $e/a_{0}^{3}$) and the Laplacian of the NCPs and BCPs at CH dissociating geometries, R, obtained with VQE, VQE* and VQE‐LD methods, using the k‐UpCCGSD *ansatz* and (4,4)‐active space, relative to CASCI(4,4).

R (Å)	Method	BCP					NCP					
		C1‐H5	C1‐H6	C1‐H2	H3‐H4	C1‐H3	C1	H2	H3	H4	H5	H6
*Electron density*												
1.3	VQE	1.75E‐4	1.75E‐4	7.20E‐4	−3.48E‐7	−4.15E‐5	−4.00E‐5	2.66E‐3	−1.45E‐3	−1.15E‐3	6.47E‐4	6.47E‐4
	VQE*	1.12E‐6	1.11E‐6	1.02E‐6	4.70E‐8	9.49E‐7	3.10E‐5	4.22E‐6	−1.47E‐5	1.08E‐5	1.47E‐6	1.47E‐6
	VQE‐LD	1.06E‐6	1.06E‐6	−6.00E‐9	2.30E‐8	9.37E‐7	3.20E‐5	3.21E‐6	−1.46E‐5	1.07E‐5	1.64E‐6	1.64E‐6
1.4	VQE	1.19E‐5	1.19E‐5	1.19E‐5	1.19E‐5	1.19E‐5	**3.90E‐5**	1.76E‐4	−1.29E‐4	−**3.25E‐5**	3.84E‐5	3.85E‐5
	VQE*	−6.46E‐7	−6.46E‐7	1.69E‐6	−1.48E‐6	1.42E‐6	1.46E‐4	1.53E‐5	−4.56E‐5	3.56E‐5	−3.06E‐6	−3.06E‐6
	VQE‐LD	−5.35E‐7	−5.35E‐7	1.66E‐6	−1.46E‐6	1.41E‐6	1.46E‐4	1.42E‐5	−4.54E‐5	3.57E‐5	−4.24E‐6	−4.24E‐6
1.6	VQE	5.07E‐6	5.07E‐6	1.15E‐5	−3.38E‐6	−3.10E‐6	−4.90E‐5	3.99E‐5	−3.29E‐5	**2.10E‐6**	9.12E‐6	9.13E‐6
	VQE*	−1.80E‐8	−1.70E‐8	3.05E‐6	−1.44E‐6	6.05E‐7	6.00E‐6	1.16E‐5	−1.85E‐5	1.04E‐5	−9.30E‐7	−9.29E‐7
	VQE‐LD	−1.73E‐7	−1.17E‐7	2.62E‐6	−1.44E‐6	7.84E‐7	8.00E‐6	1.02E‐5	−1.85E‐5	1.03E‐5	−1.18E‐6	−1.18E‐6
1.8	VQE	−4.10E‐5	−4.10E‐5	−4.43E‐5	6.66E‐3	−2.98E‐3	9.84E‐3	**6.50E‐5**	1.16E‐2	1.15E‐2	**7.88E‐5**	**7.88E‐5**
	VQE*	4.06E‐5	4.06E‐5	3.99E‐5	1.42E‐5	8.04E‐5	−5.06E‐3	4.07E‐4	8.67E‐5	−6.10E‐5	3.85E‐4	3.85E‐4
	VQE‐LD	4.58E‐5	4.53E‐5	4.47E‐5	−1.80E‐5	1.05E‐4	−5.46E‐3	4.58E‐4	3.80E‐5	−1.24E‐4	4.39E‐4	4.31E‐4
2.1	VQE	**8.16E‐6**	1.01E‐5	1.01E‐5	2.04E‐4	−4.72E‐5	−9.65E‐4	5.67E‐5	3.26E‐4	3.20E‐4	5.83E‐5	5.84E‐5
	VQE*	−1.58E‐4	2.72E‐6	1.63E‐4	4.58E‐6	1.39E‐6	−8.60E‐5	1.29E‐5	6.98E‐6	5.59E‐6	1.31E‐5	1.31E‐5
	VQE‐LD	−1.58E‐4	1.63E‐4	2.72E‐6	4.99E‐6	1.20E‐6	−8.70E‐5	1.30E‐5	8.97E‐6	6.52E‐6	1.30E‐5	1.30E‐5
*Laplacian*												
1.3	VQE	−1.53E‐3	−1.52E‐3	−2.43E‐3	3.62E‐3	−2.07E‐3	−1.32	1.81E‐1	−1.04E‐1	−5.22E‐2	3.02E‐2	3.03E‐2
	VQE*	1.13E‐4	1.13E‐4	−7.96E‐5	1.12E‐4	2.69E‐2	1.12	1.45E‐4	−9.59E‐4	7.05E‐5	5.88E‐4	5.88E‐4
	VQE‐LD	1.57E‐4	1.57E‐4	−1.46E‐3	1.00E‐4	1.64E‐4	1.16	9.08E‐5	−9.74E‐4	4.19E‐4	2.59E‐4	2.59E‐4
1.4	VQE	2.86E‐4	2.86E‐4	−7.60E‐4	−**1.89E‐3**	**4.25E‐4**	1.36	1.18E‐2	−4.95E‐3	−2.66E‐3	3.22E‐3	3.22E‐3
	VQE*	−3.88E‐4	−3.88E‐4	−2.85E‐4	−2.04E‐3	−1.04E‐3	4.99	1.52E‐3	−1.91E‐3	1.49E‐3	−6.07E‐4	−6.07E‐4
	VQE‐LD	−6.15E‐5	−6.15E‐5	2.52E‐4	−2.06E‐3	−1.03E‐3	5.05	1.43E‐3	−1.49E‐3	1.68E‐3	7.43E‐5	7.45E‐5
1.6	VQE	−2.29E‐4	−2.29E‐4	−1.38E‐3	**9.88E‐5**	2.36E‐5	−1.48	2.74E‐3	−3.65E‐3	**1.66E‐4**	7.51E‐4	7.51E‐4
	VQE*	5.53E‐5	5.54E‐5	−1.27E‐3	−3.02E‐4	9.95E‐6	0.17	1.08E‐3	−2.54E‐3	9.73E‐4	−2.16E‐4	−2.16E‐4
	VQE‐LD	1.58E‐4	2.26E‐4	−9.22E‐4	−5.80E‐4	−2.92E‐5	0.26	9.92E‐4	−1.83E‐3	1.02E‐3	−8.73E‐4	−8.73E‐4
1.8	VQE	−1.21E‐2	−1.23E‐2	**5.88E‐4**	2.01E‐2	1.67E‐2	3.27E+2	**1.70E‐3**	5.18E‐1	4.30E‐1	**1.75E‐3**	**1.75E‐3**
	VQE*	−1.95E‐3	−1.95E‐3	−1.36E‐3	4.86E‐4	−2.18E‐4	−1.68E+2	2.17E‐2	3.97E‐3	−1.84E‐3	1.40E‐2	1.40E‐2
	VQE‐LD	−1.82E‐3	−2.15E‐3	−1.73E‐3	8.47E‐4	−4.61E‐4	−1.82E+2	2.54E‐2	1.75E‐3	−4.27E‐3	1.60E‐2	1.61E‐2
2.1	VQE	−4.90E‐2	**1.17E‐4**	4.92E‐2	**3.30E‐4**	7.45E‐5	−**2.78**	**2.34E‐4**	**3.12E‐4**	**1.90E‐4**	**2.03E‐3**	**2.03E‐3**
	VQE*	6.42E‐4	−1.52E‐3	−1.64E‐3	5.04E‐4	2.15E‐5	−32.50	2.79E‐3	1.04E‐2	1.26E‐2	8.22E‐3	8.21E‐3
	VQE‐LD	6.42E‐4	−1.52E‐3	−1.64E‐3	5.04E‐4	2.15E‐5	−32.50	2.79E‐3	1.04E‐2	1.26E‐2	8.22E‐3	8.21E‐3

*Note:* Bold values highlight cases, where VQE yields smaller errors than VQE*.

On average, the three methods show small absolute discrepancies with respect to CASCI(4,4), in both the electron density ρ and its Laplacian $\nabla^{2}\rho$ at the BCPs, typically spanning $10^{-7}$ to $10^{-2}$ (Table 3). Indeed, the values of $\nabla^{2}\rho$ at BCPs have direct implications in the characterization of chemical interactions within the QTAIM framework [38].

At NCPs, the deviations in ρ are generally small, but $\nabla^{2}\rho$ exhibits larger absolute errors, reaching $10^{2}$ at the carbon nucleus for all three VQE variants. This is expected given the extreme density curvature near nuclei: the reference $\nabla^{2}\rho$ there can be as large as $10^{6}$ (Table S5), so even modest relative deviations translate into large absolute differences. Hence, reporting relative or scale‐normalized errors at NCPs is more informative than raw absolute differences. A more detailed analysis along these lines is beyond the scope of this work.

Bold entries in Table 3 indicate cases where traditional VQE yields a smaller difference than VQE* or VQE‐LD, which are just a few cases. In almost all cases, the proposed methods (VQE* and VQE‐LD) reduce the error relative to CASCI(4,4), improving the electron‐density topology. When compared to the FCI (Table S6), both VQE* and VQE‐LD approaches provided smaller errors than VQE, except for several values (14 out of 50) of the Laplacian of the density. This is likely due the convergence of the gradient or the Laplacian of the density not being included in the VQE* and VQE‐LD approaches. Yet, reducing the errors at these critical points increases the reliability in the topological classification of bonds and in the interpretations provided by QTAIM analyses of the electronic structure, in addition to indicating that the proposed modifications to VQE improve the description of molecular properties.

#### 3.1.4 Electrostatic Potential

**FIGURE 5:**
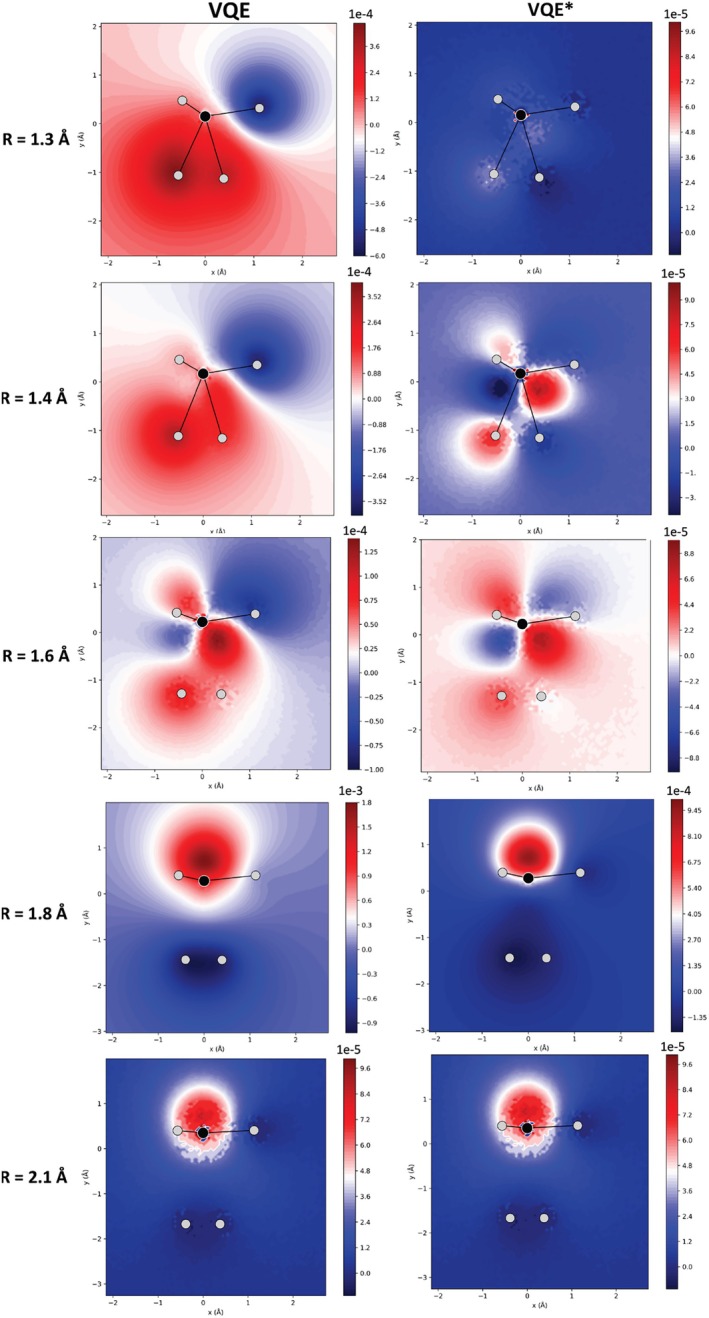
Difference in electrostatic potential (in $e/a_{0}^{3}$) along the x and y axes for dissociating CH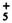 geometries, R, obtained with VQE and VQE*, using the k‐UpCCGSD *ansatz* in a (4,4)‐active space, relative to CASCI(4,4) results. One of the H atoms is superimposed on the H in the upper left corner. One H atom is superimposed on the H located in the upper‐left corner of the structure.

The differences in the electrostatic potential of VQE and VQE* relative to the CASCI(4,4) values (Figure 5) exhibit trends consistent with those observed in the differences in the electron densities. The results obtained with VQE‐LD are presented in Figure S6 and are practically the same as those obtained with VQE*. The results of the differences in electrostatic potential in relation to the CISD(4,4) are available in Figure S8.

The errors in the electrostatic potential generated with VQE* and VQE‐LD are smoother and present smaller absolute values, especially in weakly interacting dissociating structures, as indicated by the color scale (Figure 5). This corroborates what was observed in the electron density and may be an expected trend, given that the electrostatic potential is obtained from this property (Equation 11). Similarly to the electron density, the errors in the electrostatic potential obtained with converged 1‐RDM (VQE* and VQE‐LD) are *ca*. one order of magnitude smaller than standard VQE. Thus, the optimization of 1‐RDM significantly improves the description of the electrostatic potential.

#### 3.1.5 Mulliken Population Analysis

Tables 4 and 5 present the differences in Mulliken charges and populations, respectively, of CH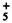 atoms at dissociating structures, obtained by VQE, VQE∗, and VQE‐LD in relation to CASCI(4,4). The values for Mulliken charges and populations are available in Table S7. Whereas the differences with respect to CISD(4,4) are available in Tables S8 and S9. Differences after the second decimal place in the population analysis obtained by different methods were not considered significant [64, 65].

**TABLE 4:** Difference in Mulliken charges (in *e*) obtained with VQE, VQE*, and VQE‐LD methods using the k‐UpCCGSD *ansatz* relative to CASCI(4,4) within the (4,4)‐active space.

R (Å)	Method	C1	H2	H3	H4	H5	H6
1.3	VQE	2.57E‐3	−5.80E‐3	3.40E‐3	2.66E‐3	−1.41E‐3	−1.41E‐3
	VQE*	1.00E‐5	−1.00E‐5	3.00E‐5	−2.00E‐5	−1.00E‐5	−1.00E‐5
	VQE‐LD	1.00E‐5	0.00	3.00E‐5	−2.00E‐5	−1.00E‐5	−1.00E‐5
1.4	VQE	1.80E‐4	−3.90E‐4	2.90E‐4	9.00E‐5	−8.00E‐5	−8.00E‐5
	VQE*	0.00	−4.00E‐5	9.00E‐5	−7.00E‐5	1.00E‐5	1.00E‐5
	VQE‐LD	−1.00E‐5	−4.00E‐5	9.00E‐5	−7.00E‐5	1.00E‐5	1.00E‐5
1.6	VQE	7.00E‐5	−1.00E‐4	7.00E‐5	**0.00**	−2.00E‐5	−2.00E‐5
	VQE*	1.00E‐5	−3.00E‐5	3.00E‐5	−2.00E‐5	0.00	0.00
	VQE‐LD	0.00	−3.00E‐5	3.00E‐5	−2.00E‐5	0.00	0.00
1.8	VQE	5.13E‐2	**‐7.00E‐5**	−2.57E‐2	−2.53E‐2	**‐1.20E‐4**	**‐1.20E‐4**
	VQE*	2.50E‐3	−8.60E‐4	−1.80E‐4	1.80E‐4	−8.20E‐4	−8.20E‐4
	VQE‐LD	2.55E‐3	−9.70E‐4	−7.00E‐5	3.30E‐4	−9.30E‐4	−9.10E‐4
2.1	VQE	1.73E‐3	−1.00E‐4	−7.00E‐4	−6.90E‐4	−1.10E‐4	−1.10E‐4
	VQE*	1.00E‐4	−2.00E‐5	−1.00E‐5	−1.00E‐5	−2.00E‐5	−2.00E‐5
	VQE‐LD	1.10E‐4	−2.00E‐5	−2.00E‐5	−1.00E‐5	−2.00E‐5	−2.00E‐5

*Note:* The values in bold indicate the cases in which VQE presented smaller errors than VQE* and VQE‐LD.

**TABLE 5:** Difference in Mulliken populations (in *e*) obtained with VQE, VQE*, and VQE‐LD methods using the k‐UpCCGSD *ansatz* relative to CASCI(4,4) within the (4,4)‐active space.

R (Å)	Method	C(1s)	C(2s)	C(2px)	C(2py)	C(2pz)	H2 (1s)	H3 (1s)	H4 (1s)	H5 (1s)	H6 (1s)
1.3	VQE	0.00	0.00	−2.49E‐3	−9.00E‐5	1.00E‐5	5.80E‐3	−3.40E‐3	−2.66E‐3	1.41E‐3	1.41E‐3
	VQE*	0.00	0.00	0.00	−1.00E‐5	0.00	1.00E‐5	−3.00E‐5	2.00E‐5	1.00E‐5	1.00E‐5
	VQE‐LD	0.00	0.00	0.00	−1.00E‐5	0.00	0.00	−3.00E‐5	2.00E‐5	1.00E‐5	1.00E‐5
1.4	VQE	0.00	1.00E‐5	−1.50E‐4	−5.00E‐5	1.00E‐5	3.90E‐4	−2.90E‐4	−9.00E‐5	8.00E‐5	8.00E‐5
	VQE*	0.00	1.00E‐5	2.00E‐5	−3.00E‐5	0.00	4.00E‐5	−9.00E‐5	7.00E‐5	−1.00E‐5	−1.00E‐5
	VQE‐LD	0.00	1.00E‐5	2.00E‐5	−3.00E‐5	0	4.00E‐5	−9.00E‐5	7.00E‐5	−1.00E‐5	−1.00E‐5
1.6	VQE	0.00	−1.00E‐5	−1.00E‐5	−8.00E‐5	3.00E‐5	1.00E‐4	−7.00E‐5	3.00E‐5	2.00E‐5	2.00E‐5
	VQE*	0.00	0.00	0.00	−1.00E‐5	0.00	3.00E‐5	−3.00E‐5	2.00E‐5	0.00	0.00
	VQE‐LD	0.00	0.00	0.00	−1.00E‐5	0.00	3.00E‐5	−3.00E‐5	2.00E‐5	0.00	0.00
1.8	VQE	2.00E‐5	2.06E‐3	1.00E‐5	−5.34E‐2	2.00E‐5	7.00E‐5	2.57E‐2	2.53E‐2	1.20E‐4	1.20E‐4
	VQE*	−1.00E‐5	−1.26E‐3	0.00	−1.25E‐3	2.00E‐5	8.60E‐4	1.80E‐4	−1.80E‐4	8.20E‐4	8.20E‐4
	VQE‐LD	−1.00E‐5	−1.38E‐3	0.00	−0.00118	2.00E‐5	0.00097	7.00E‐5	−3.30E‐4	9.30E‐4	9.10E‐4
2.1	VQE	0.00	−1.90E‐4	0.00	−1.56E‐3	2.00E‐5	1.00E‐4	7.00E‐4	6.90E‐4	1.10E‐4	1.10E‐3
	VQE*	0.00	−6.00E‐5	0.00	−4.00E‐5	0.00	2.00E‐5	1.00E‐5	1.00E‐5	2.00E‐5	2.00E‐5
	VQE‐LD	0.00	−6.00E‐5	0.00	−5.00E‐5	0.00	2.00E‐5	2.00E‐5	1.00E‐5	2.00E‐5	2.00E‐5

*Note:* All VQE* and VQE‐LD errors were smaller than those of VQE.

In general, the errors in the Mulliken charges and populations obtained with VQE* are one to two orders of magnitude smaller than the standard VQE ones (Tables 4 and 5). Only at R=1.6 Å in atom H4 and R=1.8 Å in H2, H3, and H6 does the VQE exhibit slightly smaller errors for the charges, although the differences are minimal. This improved description of Mulliken charges can be useful for analyzing, for example, charge transfer in complexes formed by hydrogen bonds [66].

### 3.2 Improving the Energy and Molecular Properties of the GateFabric *Ansatz* With Converged 1‐RDM

This section focuses on the comparisons of the results obtained with the VQE, VQE*, and VQE‐LD approaches for the GateFabric *ansatz* within the (2,2)‐active space with respect to CASCI(4,4) for CH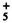 at R=1.3 and 1.4 Å. The energy differences in relation to FCI and CISD are available in Table S11.

The goal is to evaluate whether the convergence of the 1‐RDM can improve the description of the energy and molecular properties of protonated methane. The results obtained with VQE‐LD as a function of the rate f are available in Table S10, which was set to f=0.7 and 0.9 for R=1.3 and 1.4 Å, respectively, considering the best trade‐off between the number of steps and the energy errors.

The improvement in GateFabric energy with VQE* and VQE‐LD compared to the CASCI(4,4) energy is remarkable as shown in Table 6. The standard VQE approach presents errors of 0.261 and 0.146 $E_{\text{h}}$ compared to CASCI(4,4), while the relative errors of VQE* and VQE‐LD were reduced to about $10^{-3}$ $E_{\text{h}}$, achieving energies within chemical accuracy (Table 6). It is worth mentioning that the comparisons involve different active spaces, where all three VQE approaches consider a (2,2)‐active space, whereas the CASCI method employed a (4,4)‐active space. Thus, the VQE* and VQE‐LD methods achieved chemical accuracy even compared to a larger active space.

**TABLE 6:** CASCI(4,4) energy and error (in $E_{\text{h}}$) obtained between the energy of VQE, VQE*, and VQE‐LD with respect to CASCI(4,4), using the GateFabric *ansatz* and the (2,2)‐active space.

R (Å)	CASCI(4,4) energy	Error			ΔD			Steps		
		VQE	VQE*	VQE‐LD	VQE	VQE*	VQE‐LD	VQE	VQE*	VQE‐LD
1.3	−39.91925976	2.61E‐1	1.60E‐3	1.60E‐3	1.51E‐2	9.97E‐7	9.59E‐7	23	627	68
1.4	−39.91888589	1.46E‐1	1.27E‐3	1.27E‐3	1.33E‐2	9.94E‐7	8.53E‐7	41	1221	67

*Note:* The ΔD and the number of steps are presented.

Although the number of optimization steps required for VQE* and VQE‐LD was higher than for the standard VQE, this is compensated by the drastic improvement in accuracy. The VQE* method took *ca*. 30 times more steps to converge than the standard VQE. By incorporating the gradient of ΔD into the cost function, VQE‐LD reduced significantly the number of steps to convergence with only 1.6–2.9 times the steps of VQE. In other words, VQE‐LD required 559 and 1154 fewer steps than VQE*, representing a substantial gain in efficiency. The reason is that the addition of a gradient ratio term guided the optimization more effectively, avoiding flat regions in the parameter space. In contrast, in VQE*, the gradient of ΔD remained nearly constant over hundreds of iterations, slowing down convergence. The 1‐RDM obtained with CISD(4,4), CASCI(4,4), VQE, VQE*, and VQE‐LD in (2,2)‐active space and using the GateFabric *ansatz* are presented in Figure S9, while the differences in 1‐RDM obtained with CASCI(4,4) or CISD(2,2) and VQE are available in Figures S10 and S11.

**FIGURE 6:**
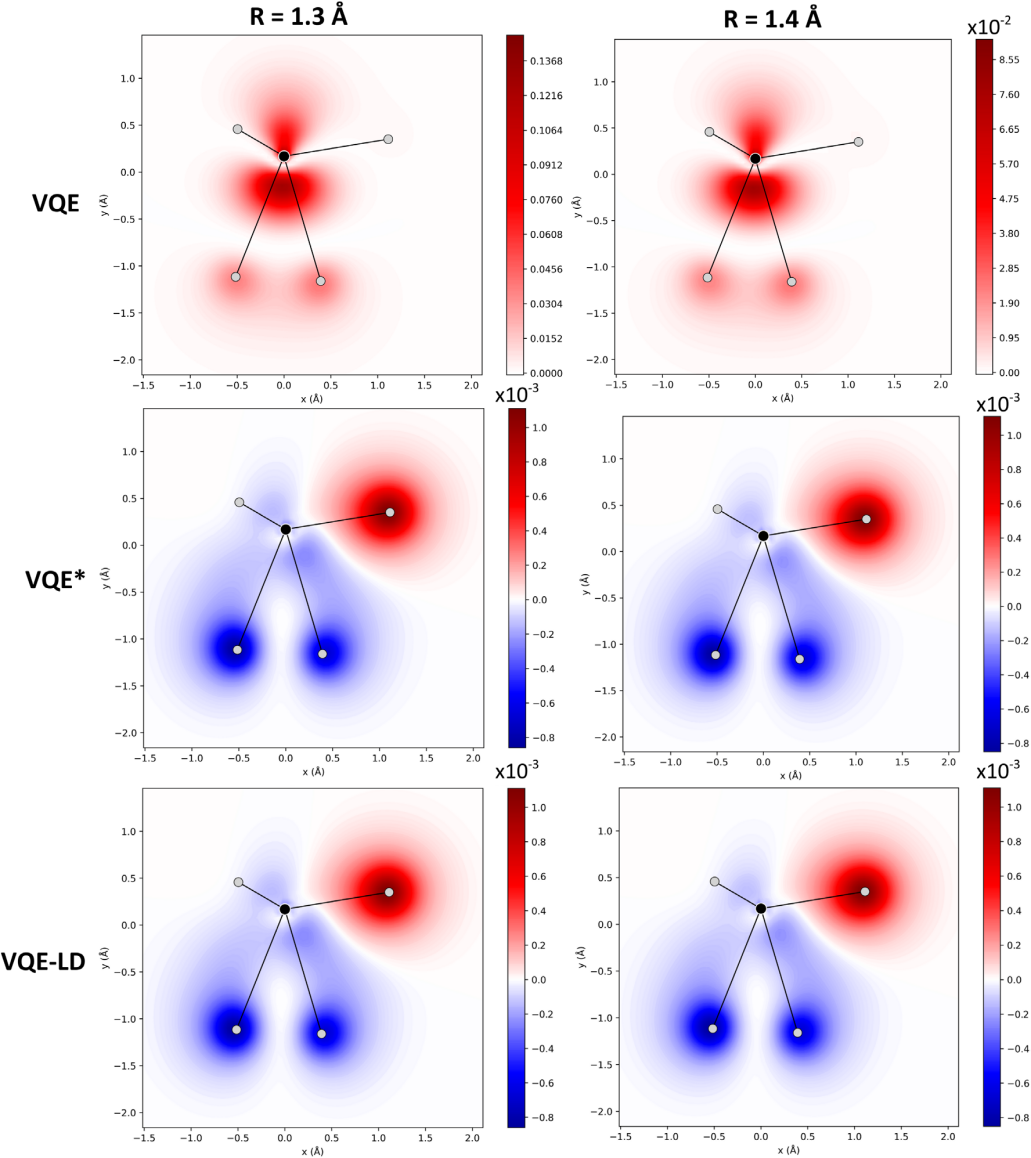
Difference in electron density (in $e/a_{0}^{3}$) along the x and y axes obtained with VQE, VQE*, and VQE‐LD GateFabric *ansatz* compared to CASCI(4,4), using the (2,2)‐active space. In the molecular layout, H3 and H4 lie at the bottom of the structure; H5 is at the upper left; H6 is positioned behind H5 (hence not visible in the figure); and H2 is located at the upper right.

The improvements in the 1‐RDM are also reflected in the molecular properties. For the standard VQE, ΔD remained on the order of $10^{-2}$, while for VQE* and VQE‐LD it was reduced to $10^{-7}$. There are almost no differences between the 1‐RDM of VQE* and VQE‐LD in relation to CASCI(4,4), while the standard VQE presents significant differences (Figure S10). This difference directly impacts the electron density (Figure 6), where the error relative to CASCI(4,4) was reduced from the first decimal place (VQE) to the seventh decimal place (VQE* and VQE‐LD). Consequently, the description of the electronic properties becomes much more precise and accurate.

Other properties, such as dipole moment (Table 7), electrostatic potential (Figure S12) and Mulliken charges and populations (Table 8), were reproduced almost exactly by VQE* and VQE‐LD, while the standard VQE presented significant deviations. Comparisons of these properties with respect to CISD(2,2) are available in Tables S12 and S13, and Figure S13. These results demonstrate that the reliability of the energy obtained with 1‐RDM optimization extends to molecular properties of chemical interest. In summary, while VQE alone fails to reach chemical precision, the 1‐RDM optimization and especially the use of the ΔD gradient led to both accurate and efficient convergence. Thus, VQE‐LD stands out as a promising strategy for larger and more complex systems, where the balance between accuracy and computational cost is crucial.

**TABLE 7:** Total dipole moment (μ) and error $\left(\Delta \mu =\mu_{\text{method}}-\mu_{\mathrm{CASCI}(4,4)}\right)$, in Debye (D), with the GateFabric (2,2)‐active space *ansatz* at CH structures (R).

R (Å)	μ (D)				Δμ (D)		
	CASCI(4,4)	VQE	VQE*	VQE‐LD	VQE	VQE*	VQE‐LD
1.3	1.9208	0.53	1.96	1.96	1.92	0.034	0.034
1.4	1.8150	0.96	1.84	1.84	1.81	0.024	0.024

**TABLE 8:** Differences (Δ=Method−CASCI(4,4)) in Mulliken charges and orbital populations (atomic charge units) obtained with VQE, VQE*, and VQE‐LD using the GateFabric *ansatz* in a (2,2)‐active space, relative to CASCI(4,4).

Atom	1.3				1.4			
	CASCI(4,4)	ΔVQE	ΔVQE*	ΔVQE‐LD	CASCI(4,4)	ΔVQE	ΔVQE*	ΔVQE‐LD
*Mulliken Charges*								
C1	−0.19698	−0.0436	0.0005	0.0005	−0.15027	−0.0371	0.0007	0.0007
H2	0.23503	−0.0045	−0.0035	−0.0035	0.22877	−0.0036	−0.0026	−0.0026
H3	0.26164	−0.0551	0.0026	0.0026	0.24301	−0.0369	0.0018	0.0018
H4	0.26599	−0.0878	0.0021	0.0021	0.24526	−0.0409	0.0014	0.0014
H5	0.21716	0.0955	−0.0009	−0.0009	0.21662	0.0593	−0.0006	−0.0006
H6	0.21716	0.0955	−0.0009	−0.0009	0.21662	0.0593	−0.0006	−0.0006
*Mulliken Populations*								
C1 (1s)	1.99280	0.0005	0.0000	0.0000	1.99302	0.0003	0.0000	0.0000
C1 (2s)	1.30083	0.0382	−0.0000	0.0000	1.31201	0.0202	−0.0000	−0.0000
C1 (2px)	1.12725	0.0011	−0.0002	−0.0002	1.13488	0.0000	−0.0003	−0.0003
C1 (2py)	0.61459	0.2772	−0.0003	−0.0003	0.55058	0.1848	−0.0004	−0.0004
C1 (2pz)	1.16151	−0.2735	0.0000	0.0000	1.15982	−0.1683	−0.0000	−0.0000
H2 (1s)	0.76497	0.0045	0.0035	0.0035	0.77123	0.0036	0.0026	0.0026
H3 (1s)	0.73836	0.0551	−0.0026	−0.0026	0.75699	0.0369	−0.0018	−0.0018
H4 (1s)	0.73401	0.0878	−0.0021	−0.0021	0.75474	0.0409	−0.0014	−0.0014
H5 (1s)	0.78284	−0.0955	0.0009	0.0009	0.78338	0.0593	0.0006	0.0006
H6 (1s)	0.78284	−0.0955	0.0009	0.0009	0.78338	0.0593	0.0006	0.0006

## 4 Conclusion

Two new approaches to converge the variational quantum eigensolver (VQE) were proposed and successfully validated. The first, VQE*, modifies the convergence criterion by including both the energy and the RMSD between consecutive one‐particle reduced density matrices (1‐RDM). The second, VQE‐LD, further incorporates the RMSD gradient of the 1‐RDM directly into the cost function. Inspired by classical electronic structure methods, these strategies aim at improving the accuracy of ground‐state energies and 1‐RDM‐dependent properties. To assess these strategies, the protonated methane CH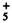 at several dissociating geometries was used as a reference system. Two *ansätze* were selected from a preliminary set, namely, k‐UpCCGSD with a (4,4)‐active space, which is energy‐accurate, and GateFabric with a (2,2)‐active space, which presents larger errors, but is represented by much simpler quantum circuits. Results were compared to CASCI(4,4) calculations within the same active spaces.

For k‐UpCCGSD, both VQE* and VQE‐LD brought no significant energy gains, because the standard VQE already agreed well with CASCI(4,4) (within $10^{-5}$–$10^{-7}$ $E_{\text{h}}$). Both methods produced nearly identical results, with VQE‐LD requiring more steps, but each substantially improved the description of molecular properties compared to standard VQE.

On the other hand, using the GateFabric *ansatz* in the (2,2)‐active space with standard VQE, the converged energies differed significantly from the CASCI(4,4) values (within 0.261–0.15 $E_{\text{h}}$). However, VQE* and VQE‐LD resulted in significant improvements in both energy accuracy and molecular properties, being virtually equal to CASCI(4,4). Therefore, incorporating 1‐RDM gradient information stabilizes the optimization and increases convergence efficiency, achieving chemical accuracy even relative to CASCI that employs a larger active space than considered in VQEs.

It was shown that the energy‐only criterion is not reliable for the convergence of VQE, when accurate molecular properties are desired. While VQE may reproduce the CASCI(4,4) energies, the associated 1‐RDM may remain poorly optimized. By incorporating the 1‐RDM either into the convergence criterion or directly into the cost function, the reliability of the wavefunction is significantly enhanced, leading to more accurate properties such as dipole moments and Mulliken charges. This strategy can also improve energy estimates, especially when the chosen *ansatz* lacks sufficient expressiveness.

The proposed method, therefore, offers a conceptually simple and computationally feasible improvement to VQE, making it especially valuable for NISQ‐era simulations, where *ansätze* are often shallow and prone to noise. Looking ahead, this strategy can be extended to larger systems, integrated into 2‐RDM refinement, or applied in multireference regimes, further advancing the reliability of quantum algorithms for molecular characterization.

## Funding

This work was supported by the Conselho Nacional de Desenvolvimento Científico e Tecnológico (Grant No. 308000/2022‐6).

## Conflicts of Interest

The authors declare no conflicts of interest.

## Supporting information



Data S1: jcc70289‐sup‐0001‐Supinfo.pdf.

Data S2: jcc70289‐sup‐0002‐Supinfo.zip.

## Data Availability

The data that supports the findings of this study are available in the Supporting Information of this article or from the corresponding author upon reasonable request, and all Python scripts required to reproduce the results are available at http://bit.ly/467CTMr.
