# Supplementary material for: Improving Energy and Molecular Properties by Convergence of the One‐Particle Reduced Density Matrix in Variational Quantum Eigensolvers (VQE)
Source: J Comput Chem. 2026 Jan 5;47(1):e70289. doi: 10.1002/jcc.70289 (PMC12766880; doi:10.1002/jcc.70289)
Supplement: Supplementary file 1 — Data S1: jcc70289‐sup‐0001‐Supinfo.pdf. [file JCC-47-0-s001.pdf]

# Supplementary Material for: Improving Energy and Molecular Properties by Convergence of the One-particle Reduced Density Matrix in Variational Quantum Eigensolvers (VQE)

Amanda Marques de Lima,[1,*] Erico Souza Teixeira,[2,†] Eivson Darlivam Rodrigues de Aguiar Silva,[1,‡] and Ricardo Luiz Longo[1,§]

[1]*Departamento de Química Fundamental, Universidade Federal de Pernambuco, Recife-PE, Brazil*
[2]*Centro de Excelência em Computação Quântica, Venturus, Campinas-SP, Brazil*

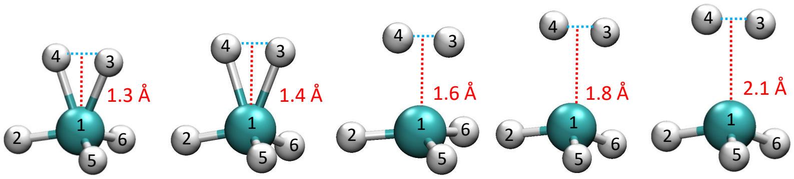


FIG. S1. Dissociation intermediate geometries of $CH_5^+$.

TABLE S1. X, Y, and Z coordinates obtained by optimizing the geometries of $CH_5^+$, R, using CISD/STO-3G.

| Atom | 1.3 | | | 1.4 | | | 1.6 | | | 1.8 | | | 2.1 | | |
|---|---|---|---|---|---|---|---|---|---|---|---|---|---|---|---|
| | X | Y | Z | X | Y | Z | X | Y | Z | X | Y | Z | X | Y | Z |
| C1 | 0 | 0.152552 | 0 | 0 | 0.168216 | 0 | 0 | 0.226127 | 0 | 0 | 0.280210 | 0 | 0 | 0.353191 | 0 |
| H2 | 1.116559 | 0.321770 | 0 | 1.112783 | 0.349956 | 0 | 1.114812 | 0.391534 | 0 | 1.124328 | 0.398212 | 0 | 1.134594 | 0.409237 | 0 |
| H3 | -0.555030 | -1.061130 | 0 | -0.515290 | -1.115260 | 0 | -0.436220 | -1.285270 | 0 | -0.404350 | -1.438190 | 0 | -0.382790 | -1.667340 | 0 |
| H4 | 0.381302 | -1.130930 | 0 | 0.392186 | -1.159990 | 0 | 0.400270 | -1.297120 | 0 | 0.391958 | -1.443000 | 0 | 0.378729 | -1.670040 | 0 |
| H5 | -0.471420 | 0.477487 | 0.959211 | -0.494840 | 0.457998 | 0.960102 | -0.539430 | 0.417047 | 0.967258 | -0.555970 | 0.400858 | 0.975637 | -0.565270 | 0.404500 | 0.983819 |
| H6 | -0.471420 | 0.477487 | -0.959210 | -0.494840 | 0.457998 | -0.960100 | -0.539430 | 0.417047 | -0.967260 | -0.555970 | 0.400858 | -0.975640 | -0.565270 | 0.404500 | -0.983820 |

TABLE S2. CISD(4,4) and FCI energies, and associated errors (in $E_h$) for the VQE, VQE*, and VQE-LD methods using the k-UpCCGSD *ansatz* within the (4,4) active space for $CH_5^+$ structures at different bond lengths ($R$).

| R (Å) | Energy CISD ($E_h$) | Error ($\times 10^{-6}$ $E_h$) | | | Energy FCI ($E_h$) | Error ($\times 10^{-2}$ $E_h$) | | |
|---|---|---|---|---|---|---|---|---|
| | | VQE | VQE* | VQE-LD | | VQE | VQE* | VQE-LD |
| 1.3 | -39.91925797 | 2.20 | 1.51 | 1.50 | -40.01161394 | 9.23582 | 9.23575 | 9.2357 |
| 1.4 | -39.91888320 | 0.30 | -0.15 | -0.14 | -40.01134578 | 9.24629 | 9.24624 | 9.2462 |
| 1.6 | -39.91533341 | 2.73 | 2.13 | 2.13 | -40.00626161 | 9.09309 | 9.09303 | 9.0930 |
| 1.8 | -39.91716922 | 67.10 | 56.50 | 56.20 | -39.99865922 | 8.15571 | 8.15465 | 8.1546 |
| 2.1 | -39.90742554 | 1.38 | -0.66 | -0.70 | -39.98924407 | 8.18199 | 8.18179 | 8.1818 |

TABLE S3. Energy (in $E_h$) and number of steps obtained with VQE-LD for the $CH_5^+$ dissociation geometries with different rates, $f$, using k-UpCCGSD within a (4,4)-active space.

| $f$ | 1.3 | | 1.4 | | 1.6 | | 1.8 | | 2.1 | |
|---|---|---|---|---|---|---|---|---|---|---|
| | Energy | Steps | Energy | Steps | Energy | Steps | Energy | Steps | Energy | Steps |
| 0.05 | -39.919256465 | 21 | -39.918883345 | 20 | -39.915331281 | 18 | -39.917112651 | 40 | -39.907426216 | 41 |
| 0.10 | -39.919256465 | 22 | -39.918883345 | 21 | -39.915331281 | 19 | -39.917113008 | 41 | -39.907426223 | 42 |
| 0.30 | -39.919256467 | 27 | -39.918883345 | 26 | -39.915331281 | 19 | -39.917190294 | 1137 | -39.907426279 | 50 |
| 0.60 | -39.919256641 | 525 | -39.918883345 | 33 | -39.915331281 | 20 | -39.917190475 | 1409 | -39.907431312 | 2256 |
| 1.0 | -39.919256771 | 1000 | -39.918883345 | 34 | -39.915331281 | 26 | -39.917190512 | 1613 | -39.907431449 | 2939 |

* amanda.aml002@gmail.com
† Correspondence to: E. S. Teixeira (E-mail: erico.teixeira@venturus.org.br)
‡ eivsondras@gmail.com
§ Correspondence to: R. L. Longo (E-mail: ricardo.longo@ufpe.br)

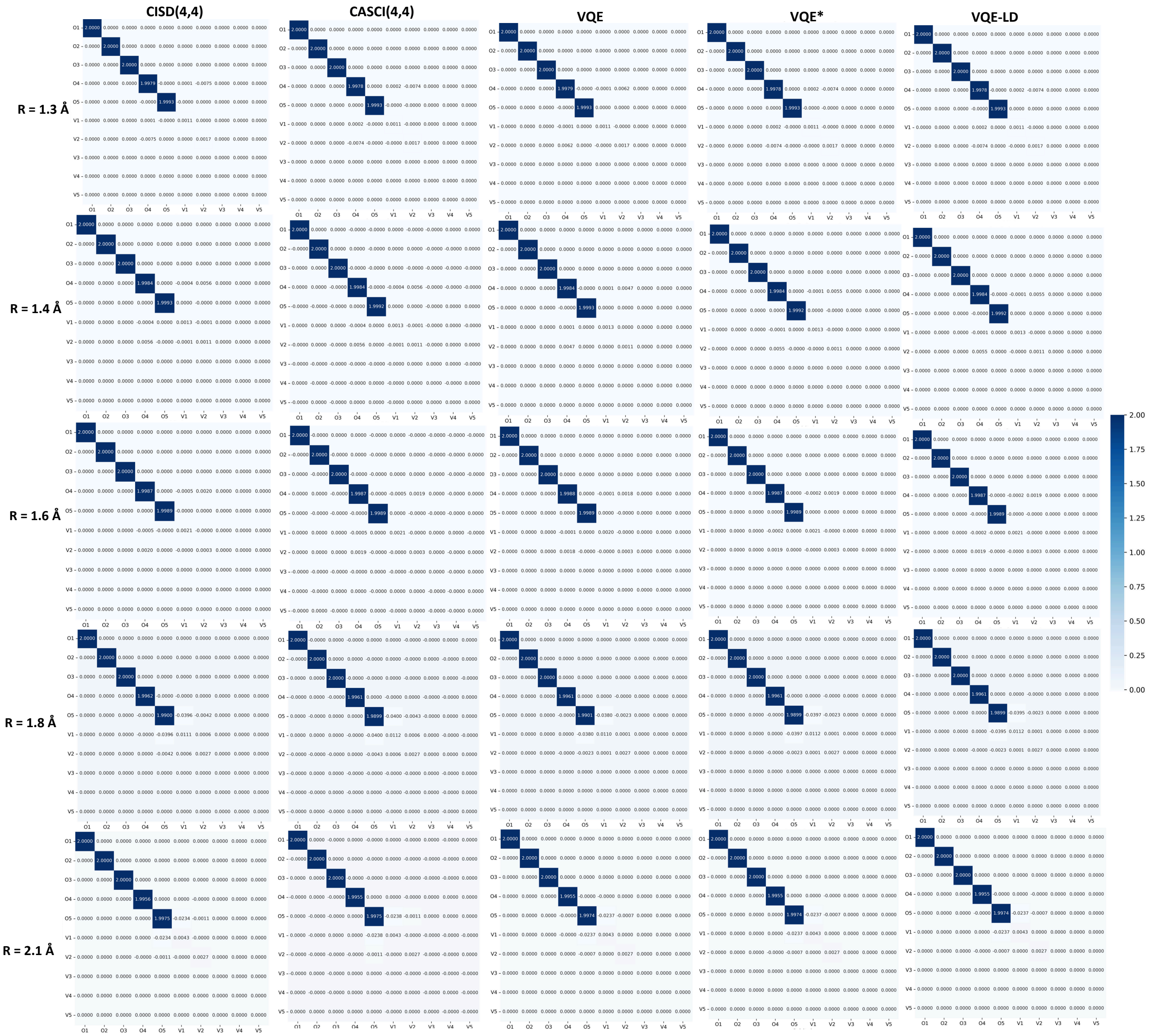


FIG. S2. 1-RDM of the intermediate dissociation geometries of $CH_5^+$, R, obtained with CISD, CASCI, VQE, VQE* and VQE-LD in the (4,4)-active space and k-UpCCGSD *ansatz*. O1 to O5 refer to the occupied orbitals, while V1 to V5 are the virtual orbitals.

TABLE S4. Total dipole moment ($\mu$) and error ($\Delta\mu = \mu_{\text{method}} - \mu_{\text{CISD}}$), in Debye (D), with the k-UpCCGSD (4,4)-active space *ansatz* at $CH_5^+$ structures ($R$).

| **R** (Å) | $\mu$ (D) | | | | $\Delta\mu$ ($\times 10^{-3}$ D) | | |
|---|---|---|---|---|---|---|---|
| | **CISD** | **VQE** | **VQE*** | **VQE-LD** | **VQE** | **VQE*** | **VQE-LD** |
| 1.3 | 1.9210 | 1.9252 | 1.9209 | 1.9209 | 4.23 | -0.10 | -0.10 |
| 1.4 | 1.8149 | 1.8178 | 1.8150 | 1.8145 | 2.89 | 0.12 | -040 |
| 1.6 | 1.1189 | 1.1196 | 1.1189 | 1.1190 | 0.70 | 0.00 | 0.10 |
| 1.8 | 0.5788 | 0.5672 | 0.5780 | 0.5791 | -11.60 | -0.82 | 0.30 |
| 2.1 | 0.6785 | 0.6913 | 0.6766 | 0.6768 | 12.80 | -1.95 | -1.70 |

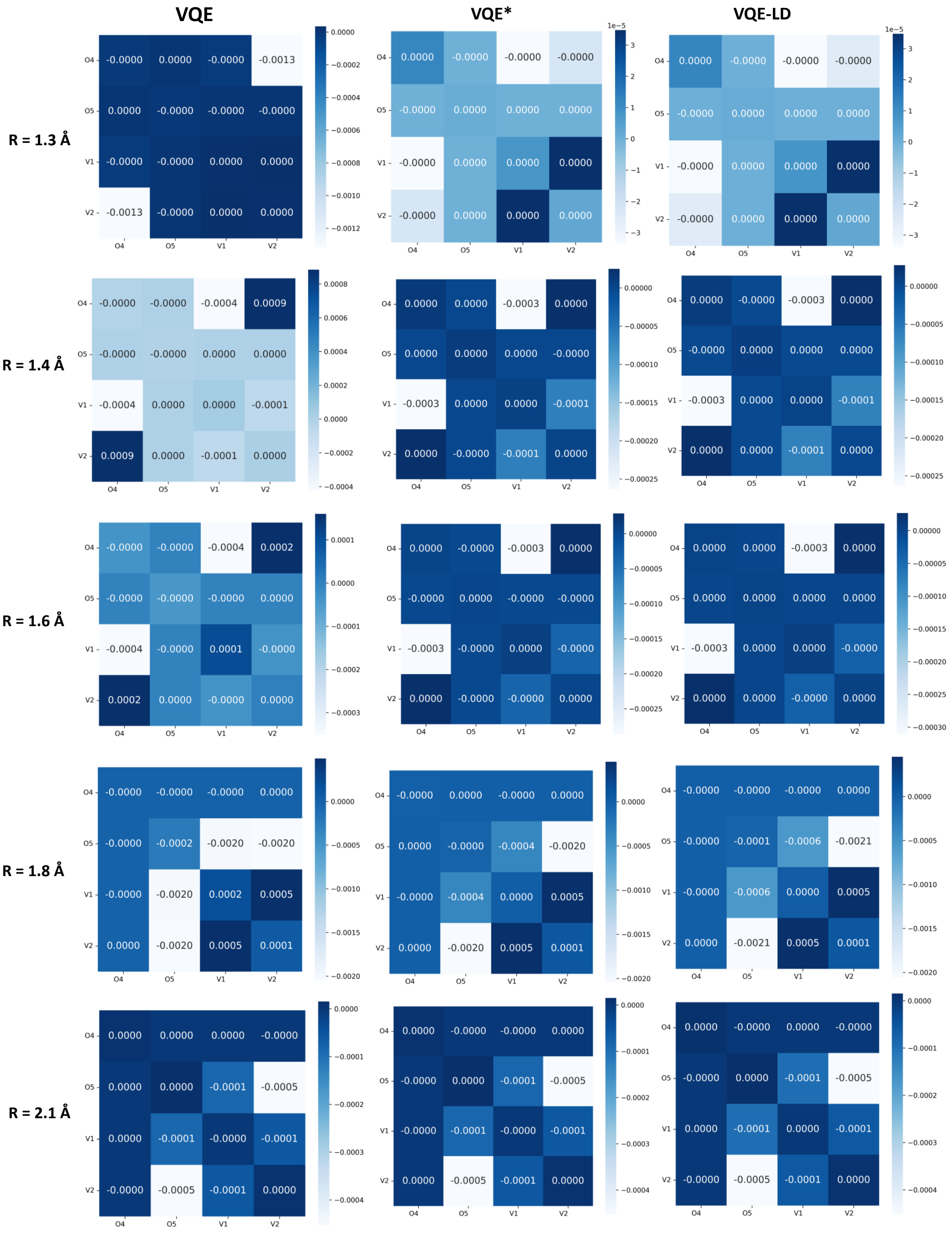


FIG. S3. 1-RDM differences for VQE, VQE, and VQE-LD with respect to CASCI(4,4)* using the k-UpCCGSD *ansatz* in a (4,4)-active space. O4 and O5 refer to the last two occupied orbitals, while V1 and V2 are the first two virtual orbitals.

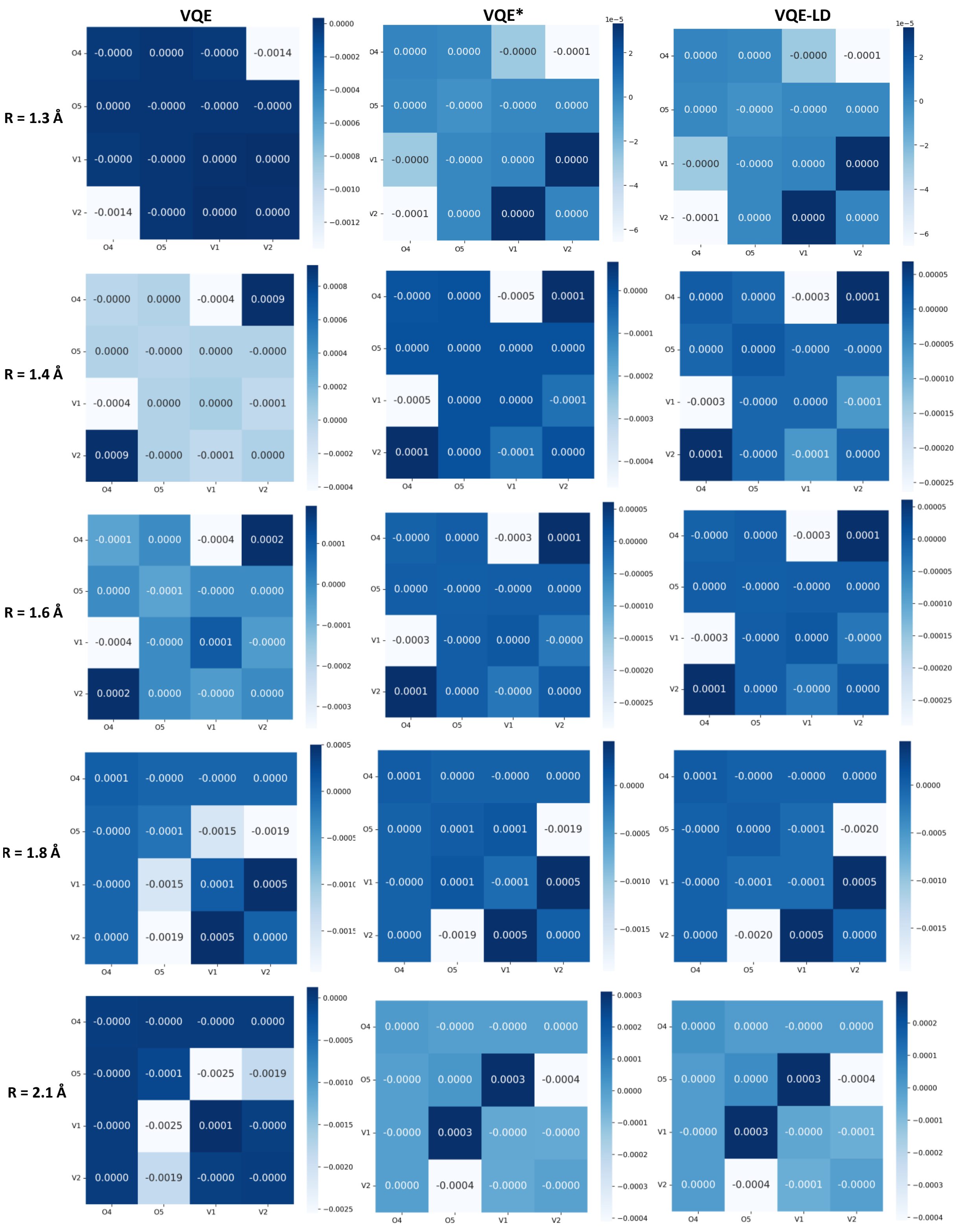

FIG. S4. 1-RDM differences for VQE, VQE, and VQE-LD with respect to CISD(4,4)* using the k-UpCCGSD *ansatz* in a (4,4) active space. O4 and O5 refer to the last two occupied orbitals, while V1 and V2 are the first two virtual orbitals.

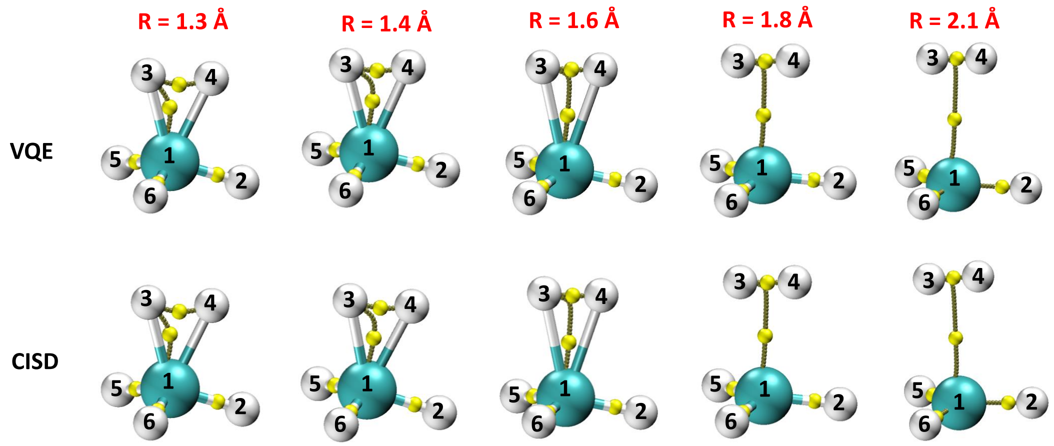


FIG. S5. Critical points of the bonds in the $CH_5^+$ dissociation geometries, obtained using the CASCI(4,4) method and with the VQE without 1-RDM optimization (k-UpCCGSD *anstaz* and (4,4)-active space), both with the STO-3G basis. The results with VQE* and VQE-LD are similar to those of VQE.

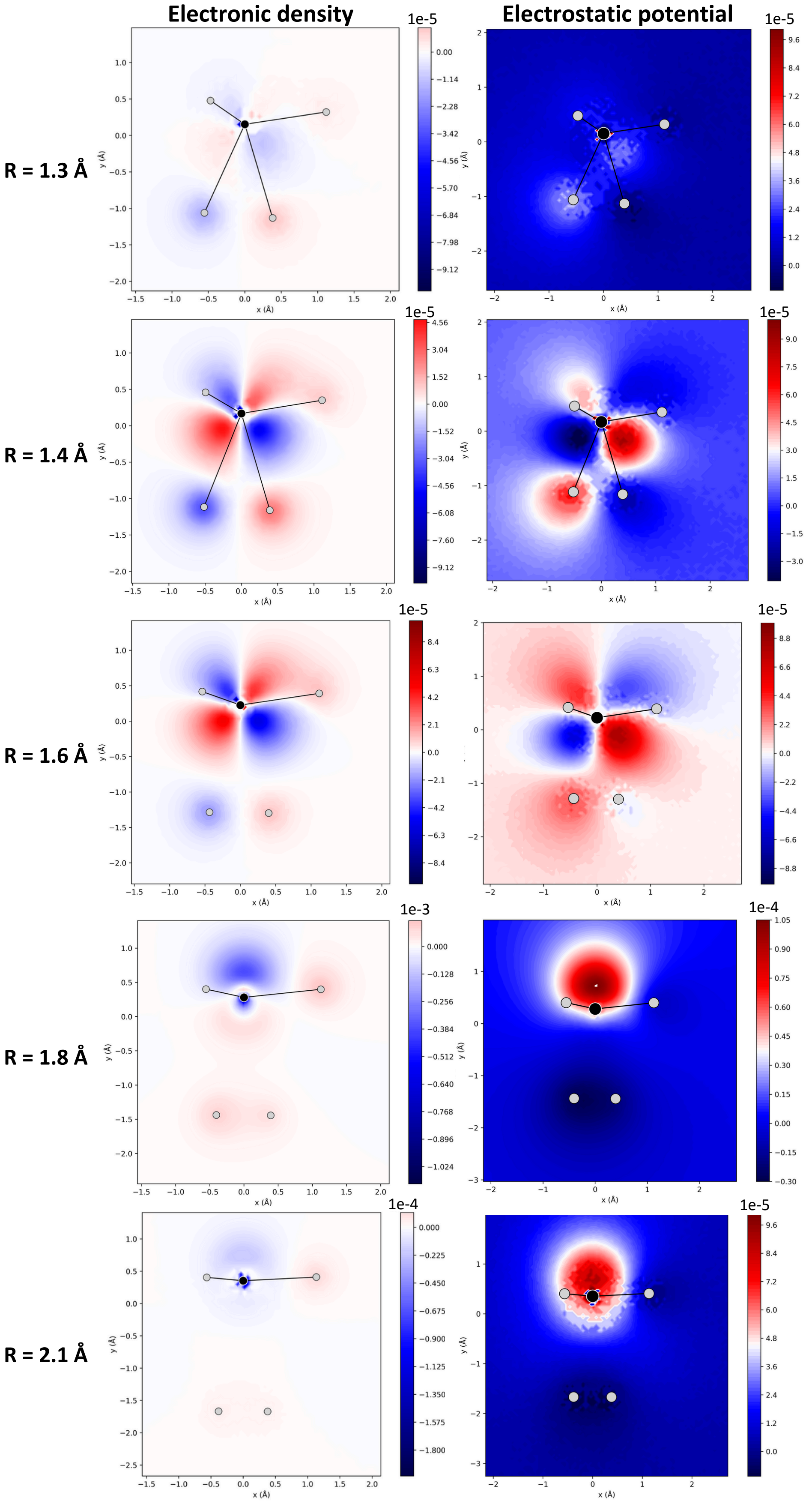


FIG. S6. Difference in electron density (in $e/a_0^3$) and electrostatic potential (in $e/a_0$) along the $x$ and $y$ axes between VQE-LD with k-UpCCGSD *ansatz* and CASCI within the same (4,4)-active space.

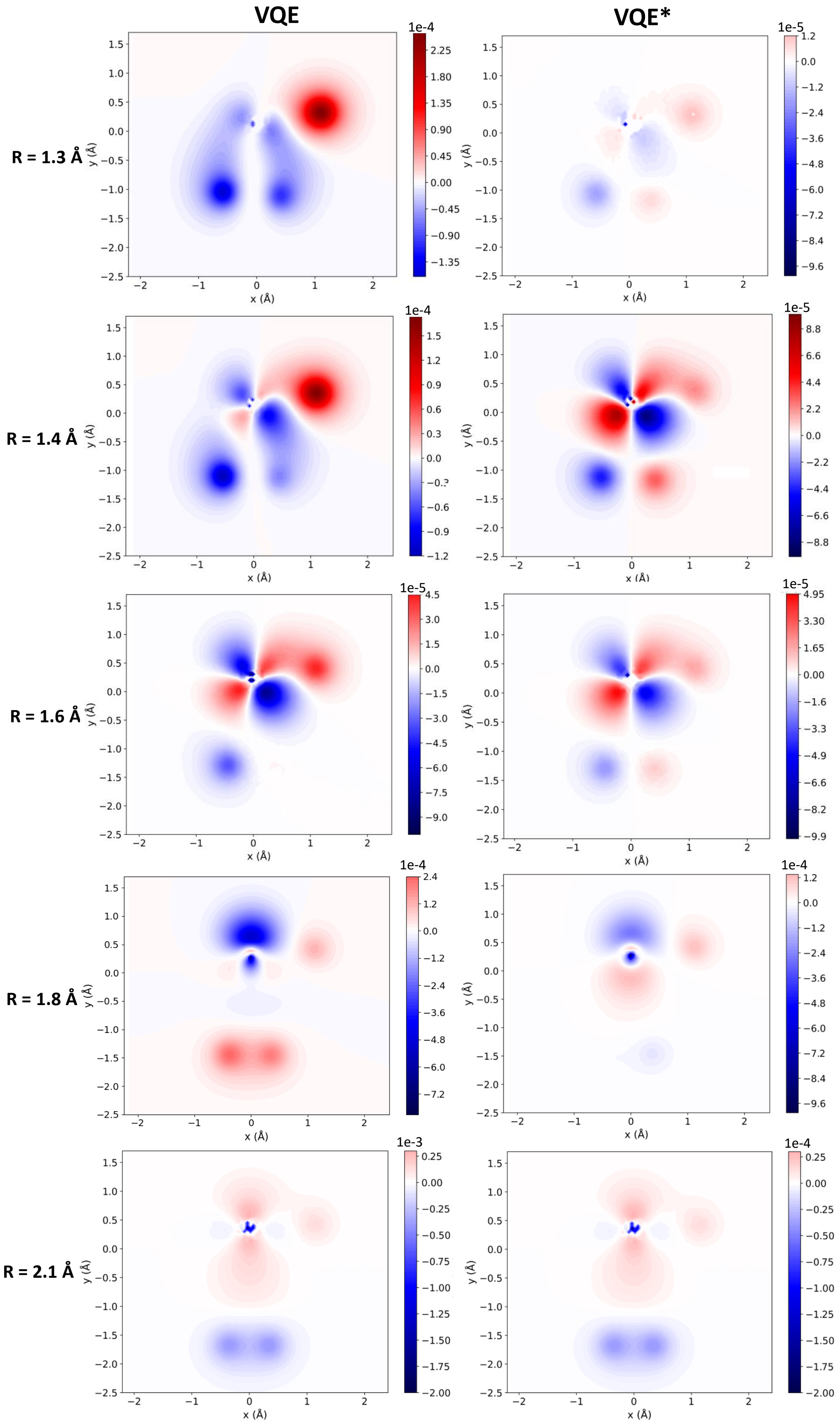


FIG. S7. Difference in electron density (in $e/a_0^3$) along the $x$ and $y$ axes of $CH_5^+$ dissociating geometries, obtained with VQE, VQE* and VQE-LD methods with the k-UpCCGSD *ansatz* relative to CISD within a (4,4)-active space.

TABLE S5. Electron density and Laplacian of the $CH_5^+$ dissociation geometries with VQE, VQE* and VQE-LD compared to CASCI(4,4). The k-UpCCGSD and (4,4)-active space were used.

| CP | 1.3 | | | | 1.4 | | | | 1.6 | | | | 1.8 | | | | 2.1 | | | |
|---|---|---|---|---|---|---|---|---|---|---|---|---|---|---|---|---|---|---|---|---|
| | **CASCI** | **VQE** | **VQE*** | **VQE-LD** | **CASCI** | **VQE** | **VQE*** | **VQE-LD** | **CASCI** | **VQE** | **VQE*** | **VQE-LD** | **CASCI** | **VQE** | **VQE-LD** | **VQE-LD** | **CASCI** | **VQE** | **VQE-LD** | **VQE-LD** |
| | **Electron density** | | | | | | | | | | | | | | | | | | | |
| C | 1.02E+2 | 1.02E+2 | 1.02E+2 | 1.02E+2 | 1.22E+2 | 1.22E+2 | 1.22E+2 | 1.22E+2 | 1.18E+2 | 1.18E+2 | 1.18E+2 | 1.18E+2 | 9.60E+1 | 9.60E+1 | 9.60E+1 | 9.60E+1 | 1.27E+2 | 1.27E+2 | 1.27E+2 | 1.27E+2 |
| H2 | 3.01E-1 | 3.04E-1 | 3.01E-1 | 3.01E-1 | 3.05E-1 | 3.05E-1 | 3.05E-1 | 3.05E-1 | 3.08E-1 | 3.08E-1 | 3.08E-1 | 3.08E-1 | 3.04E-1 | 3.04E-1 | 3.04E-1 | 3.04E-1 | 2.99E-1 | 2.99E-1 | 2.99E-1 | 2.99E-1 |
| H3 | 2.62E-1 | 2.61E-1 | 2.62E-1 | 2.62E-1 | 2.73E-1 | 2.73E-1 | 2.73E-1 | 2.73E-1 | 2.91E-1 | 2.91E-1 | 2.91E-1 | 2.91E-1 | 3.20E-1 | 3.32E-1 | 3.20E-1 | 3.20E-1 | 3.56E-1 | 3.56E-1 | 3.56E-1 | 3.56E-1 |
| H4 | 2.64E-1 | 2.63E-1 | 2.64E-1 | 2.64E-1 | 2.69E-1 | 2.69E-1 | 2.69E-1 | 2.69E-1 | 3.00E-1 | 3.00E-1 | 3.00E-1 | 3.00E-1 | 3.22E-1 | 3.34E-1 | 3.22E-1 | 3.22E-1 | 3.53E-1 | 3.54E-1 | 3.53E-1 | 3.53E-1 |
| H5 | 3.16E-1 | 3.16E-1 | 3.16E-1 | 3.16E-1 | 3.10E-1 | 3.10E-1 | 3.10E-1 | 3.10E-1 | 3.05E-1 | 3.05E-1 | 3.05E-1 | 3.05E-1 | 3.08E-1 | 3.08E-1 | 3.08E-1 | 3.08E-1 | 2.83E-1 | 2.83E-1 | 2.83E-1 | 2.83E-1 |
| H6 | 3.16E-1 | 3.16E-1 | 3.16E-1 | 3.16E-1 | 3.10E-1 | 3.10E-1 | 3.10E-1 | 3.10E-1 | 3.05E-1 | 3.05E-1 | 3.05E-1 | 3.05E-1 | 3.08E-1 | 3.08E-1 | 3.08E-1 | 3.08E-1 | 2.83E-1 | 2.83E-1 | 2.83E-1 | 2.83E-1 |
| C-H5 | 2.53E-1 | 2.53E-1 | 2.53E-1 | 2.53E-1 | 2.53E-1 | 2.53E-1 | 2.53E-1 | 2.53E-1 | 2.51E-1 | 2.51E-1 | 2.51E-1 | 2.51E-1 | 2.47E-1 | 2.47E-1 | 2.47E-1 | 2.47E-1 | 2.43E-1 | 2.44E-1 | 2.43E-1 | 2.43E-1 |
| C-H6 | 2.53E-1 | 2.53E-1 | 2.53E-1 | 2.53E-1 | 2.53E-1 | 2.53E-1 | 2.53E-1 | 2.53E-1 | 2.51E-1 | 2.51E-1 | 2.51E-1 | 2.51E-1 | 2.47E-1 | 2.47E-1 | 2.47E-1 | 2.47E-1 | 2.43E-1 | 2.43E-1 | 2.43E-1 | 2.44E-1 |
| C-H2 | 2.41E-1 | 2.41E-1 | 2.41E-1 | 2.41E-1 | 2.44E-1 | 2.44E-1 | 2.44E-1 | 2.44E-1 | 2.48E-1 | 2.48E-1 | 2.48E-1 | 2.48E-1 | 2.46E-1 | 2.46E-1 | 2.47E-1 | 2.47E-1 | 2.44E-1 | 2.43E-1 | 2.44E-1 | 2.43E-1 |
| H3-H4 | 1.40E-1 | 1.40E-1 | 1.40E-1 | 1.40E-1 | 1.47E-1 | 1.47E-1 | 1.47E-1 | 1.47E-1 | 1.76E-1 | 1.76E-1 | 1.76E-1 | 1.76E-1 | 1.99E-1 | 2.06E-1 | 1.99E-1 | 1.99E-1 | 2.31E-1 | 2.31E-1 | 2.31E-1 | 2.31E-1 |
| C-H3 | 1.33E-1 | 1.32E-1 | 1.33E-1 | 1.33E-1 | 1.21E-1 | 1.21E-1 | 1.21E-1 | 1.21E-1 | 7.97E-2 | 7.97E-2 | 7.97E-2 | 7.97E-2 | 5.21E-2 | 4.91E-2 | 5.22E-2 | 5.22E-2 | 2.51E-2 | 2.50E-2 | 2.51E-2 | 2.51E-2 |
| | **Laplacian** | | | | | | | | | | | | | | | | | | | |
| C | -3.39 E+6 | -3.39 E+6 | -3.39 E+6 | -3.39 E+6 | -4.09 E+6 | -4.09 E+6 | -4.09 E+6 | -4.09 E+6 | -3.97 E+6 | -3.97 E+6 | -3.97 E+6 | -3.97 E+6 | -3.19 E+6 | -3.19 E+6 | -3.19 E+6 | -3.19 E+6 | -4.28 E+6 | -4.28 E+6 | -4.28 E+6 | -4.28 E+6 |
| H2 | 2.17E+1 | 2.19E+1 | 2.17E+1 | 2.17E+1 | 2.04E+1 | 2.04E+1 | 2.04E+1 | 2.04E+1 | 1.76E+1 | 1.77E+1 | 1.77E+1 | 1.76E+1 | 1.86E+1 | 1.86E+1 | 1.86E+1 | 1.86E+1 | 1.41E+1 | 1.41E+1 | 1.41E+1 | 1.41E+1 |
| H3 | 1.99E+1 | 1.98E+1 | 1.99E+1 | 1.99E+1 | 1.24E+1 | 1.24E+1 | 1.24E+1 | 1.24E+1 | 3.10E+1 | 3.10E+1 | 3.10E+1 | 3.10E+1 | 1.48E+1 | 1.53E+1 | 1.48E+1 | 1.48E+1 | 1.18E+1 | 1.18E+1 | 1.18E+1 | 1.18E+1 |
| H4 | 1.33E+1 | 1.32E+1 | 1.33E+1 | 1.33E+1 | 1.74E+1 | 1.74E+1 | 1.74E+1 | 1.74E+1 | 1.57E+1 | 1.57E+1 | 1.57E+1 | 1.57E+1 | 1.25E+1 | 1.29E+1 | 1.25E+1 | 1.25E+1 | 1.42E+1 | 1.42E+1 | 1.42E+1 | 1.42E+1 |
| H5 | 1.59E+1 | 1.59E+1 | 1.59E+1 | 1.59E+1 | 2.49E+1 | 2.49E+1 | 2.49E+1 | 2.49E+1 | 2.59E+1 | 2.59E+1 | 2.59E+1 | 2.59E+1 | 1.28E+1 | 1.28E+1 | 1.28E+1 | 1.28E+1 | 4.25E+1 | 4.26E+1 | 4.25E+1 | 4.25E+1 |
| H6 | 1.59E+1 | 1.59E+1 | 1.59E+1 | 1.59E+1 | 2.49E+1 | 2.49E+1 | 2.49E+1 | 2.49E+1 | 2.59E+1 | 2.59E+1 | 2.59E+1 | 2.59E+1 | 1.28E+1 | 1.28E+1 | 1.28E+1 | 1.28E+1 | 4.25E+1 | 4.25E+1 | 4.25E+1 | 4.25E+1 |
| C-H5 | -7.43E-1 | -7.42E-1 | -7.43E-1 | -7.44E-1 | -7.43E-1 | -7.55E-1 | 7.55E-1 | -7.55E-1 | -7.59E-1 | -7.59E-1 | -7.59E-1 | -7.59E-1 | -7.78E-1 | -7.65E-1 | -7.75E-1 | -7.76E-1 | -7.51E-1 | -7.02E-1 | -7.51E-1 | -7.51E-1 |
| C-H6 | -7.43E-1 | -7.42E-1 | -7.44E-1 | -7.44E-1 | -7.43E-1 | -7.55E-1 | 7.55E-1 | -7.55E-1 | -7.59E-1 | -7.59E-1 | -7.59E-1 | -7.59E-1 | -7.77E-1 | -7.65E-1 | -7.76E-1 | -7.76E-1 | -7.05E-1 | -7.06E-1 | -7.04E-1 | -7.05E-1 |
| C-H2 | -6.73E-1 | -6.72E-1 | -6.74E-1 | -6.73E-1 | -6.98E-1 | -6.98E-1 | 6.99E-1 | -6.99E-1 | -7.43E-1 | -7.41E-1 | -7.42E-1 | -7.42E-1 | -7.59E-1 | -7.59E-1 | -7.57E-1 | -7.57E-1 | -7.02E-1 | -7.51E-1 | -7.00E-1 | -7.01E-1 |
| H3-H4 | -7.64E-2 | -7.98E-2 | -7.63E-2 | -7.62E-2 | -1.09E-1 | -1.09E-1 | 1.08E-1 | -1.08E-1 | -3.07E-1 | -3.07E-1 | -3.06E-1 | -3.06E-1 | -4.50E-1 | -4.70E-1 | -4.50E-1 | -4.51E-1 | -6.73E-1 | -6.73E-1 | -6.73E-1 | -6.73E-1 |
| C-H3 | -1.08E-1 | -1.06E-1 | -1.08E-1 | -1.08E-1 | -8.09E-2 | -8.09E-2 | 7.99E-2 | -7.99E-2 | 4.17E-2 | 4.17E-2 | 4.17E-2 | 4.16E-2 | 8.04E-2 | 9.70E-2 | 8.02E-2 | 7.99E-2 | 7.85E-2 | 7.85E-2 | 7.85E-2 | 7.83E-2 |

TABLE S6. Differences in the electron density (in $e/a_0^3$) and in the Laplacian at the NCP and BCP for dissociating $CH_5^+$ geometries, R, computer with VQE, VQE*, and VQE-LD methods, using k-UpCCGSD *ansatz* within the (4,4)-active space, relative to full-CI. Bold values highlight cases where VQE yields smaller errors than VQE*.

| | | **BCP** | | | | | **NCP** | | | | | |
|---|---|---|---|---|---|---|---|---|---|---|---|---|
| **R** (Å) | **Method** | C1-H5 | C1-H6 | C1-H2 | H3-H4 | C1-H3 | C1 | H2 | H3 | H4 | H5 | H6 |
| | | | | | | **Electron density** | | | | | | |
| | VQE | 3.19E-03 | 3.19E-03 | 3.97E-03 | 2.98E-03 | 1.16E-03 | **3.15E-04** | **-6.03E-03** | -3.79E-03 | -4.26E-03 | -7.35E-03 | -7.35E-03 |
| 1.3 | VQE* | 3.01E-03 | 3.01E-03 | 3.25E-03 | 2.98E-03 | 1.03E-03 | 3.86E-04 | -8.69E-03 | -2.36E-03 | -3.10E-03 | -7.99E-03 | -7.99E-03 |
| | VQE-LD | 3.01E-03 | 3.01E-03 | 3.25E-03 | 2.98E-03 | 1.03E-03 | 3.87E-04 | -8.69E-03 | -2.36E-03 | -3.10E-03 | -7.99E-03 | -7.99E-03 |
| | VQE | 3.05E-03 | 3.05E-03 | 3.05E-03 | 3.55E-03 | 3.05E-03 | 5.03E-03 | -8.56E-03 | -1.57E-03 | -1.95E-03 | -8.08E-03 | -8.08E-03 |
| 1.4 | VQE* | 3.03E-03 | 3.03E-03 | 3.35E-03 | 3.52E-03 | 2.39E-04 | 5.13E-03 | -8.52E-03 | -1.48E-03 | -1.88E-03 | -8.08E-03 | -8.08E-03 |
| | VQE-LD | 3.03E-03 | 3.03E-03 | 3.35E-03 | 3.52E-03 | 2.39E-04 | 5.13E-03 | -8.52E-03 | -1.48E-03 | -1.88E-03 | -8.08E-03 | -8.08E-03 |
| | VQE | 2.98E-03 | 2.98E-03 | 3.21E-03 | 5.59E-03 | -1.64E-03 | 1.78E-02 | -9.13E-03 | 1.75E-03 | 1.63E-03 | -9.05E-03 | -9.05E-03 |
| 1.6 | VQE* | 2.97E-03 | 2.97E-03 | 3.20E-03 | 5.59E-03 | -1.63E-03 | 1.77E-02 | -9.13E-03 | 1.75E-03 | 1.63E-03 | -9.05E-03 | -9.05E-03 |
| | VQE-LD | 2.97E-03 | 2.97E-03 | 3.20E-03 | 5.59E-03 | -1.63E-03 | 1.77E-02 | -9.13E-03 | 1.75E-03 | 1.63E-03 | -9.05E-03 | -9.05E-03 |
| | VQE | 2.78E-03 | 2.78E-03 | 3.13E-03 | 8.68E-03 | -2.85E-03 | 2.69E-02 | -8.68E-03 | 6.33E-03 | 6.30E-03 | -9.52E-03 | -9.52E-03 |
| 1.8 | VQE* | 2.75E-03 | 2.75E-03 | 3.12E-03 | 2.04E-03 | 2.12E-04 | 1.20E-02 | -8.60E-03 | -5.15E-03 | -5.27E-03 | -9.22E-03 | -9.22E-03 |
| | VQE-LD | 2.76E-03 | 2.75E-03 | 3.12E-03 | 2.01E-03 | 2.37E-04 | 1.15E-02 | -8.55E-03 | -5.20E-03 | -5.33E-03 | -9.16E-03 | -9.17E-03 |
| | VQE | 2.76E-03 | 2.60E-03 | 2.77E-03 | 3.38E-03 | -1.40E-04 | 2.26E-02 | -8.59E-03 | -3.95E-03 | -3.95E-03 | -8.55E-03 | -8.55E-03 |
| 2.1 | VQE* | 2.59E-03 | 2.59E-03 | 2.92E-03 | 3.18E-03 | -9.17E-05 | 2.25E-02 | -8.58E-03 | -3.94E-03 | -3.93E-03 | -8.54E-03 | -8.54E-03 |
| | VQE-LD | 2.59E-03 | 2.75E-03 | 2.76E-03 | 3.18E-03 | -9.18E-05 | 2.25E-02 | -8.58E-03 | -3.94E-03 | -3.93E-03 | -8.54E-03 | -8.54E-03 |
| | | | | | | **Laplacian** | | | | | | |
| | VQE | 9.99E-02 | 9.99E-02 | **5.73E-02** | **2.72E-02** | 1.45E-02 | 1.30E+01 | **-3.43E-01** | -2.21E-01 | **-1.47E-01** | **-2.65E-01** | **-2.65E-01** |
| 1.3 | VQE* | 9.99E-02 | 9.96E-02 | 5.97E-02 | 2.37E-02 | 1.04E-02 | 1.27E+01 | -5.24E-01 | -1.18E-01 | -9.47E-02 | -2.94E-01 | -2.94E-01 |
| | VQE-LD | 9.99E-02 | 9.96E-02 | 5.83E-02 | 2.37E-02 | 1.67E-02 | 1.28E+01 | -5.24E-01 | -1.18E-01 | -9.44E-02 | -2.94E-01 | -2.95E-01 |
| | VQE | 5.61E-02 | 5.61E-02 | **6.15E-02** | 5.44E-02 | 2.03E-02 | 1.79E+02 | -4.67E-01 | -1.11E-02 | -6.09E-02 | -5.45E-01 | -5.45E-01 |
| 1.4 | VQE* | 5.54E-02 | 5.54E-02 | 6.20E-02 | 5.42E-02 | 1.92E-02 | 1.72E+02 | -4.67E-01 | -8.10E-03 | -5.68E-02 | -5.45E-01 | -5.45E-01 |
| | VQE-LD | 5.57E-02 | 5.57E-02 | 6.25E-02 | 5.42E-02 | 1.92E-02 | 1.72E+02 | -4.67E-01 | -7.69E-03 | -5.66E-02 | -5.44E-01 | -5.44E-01 |
| | VQE | 6.54E-02 | 6.54E-02 | 2.23E-02 | 4.38E-02 | 1.09E-02 | 6.02E+02 | **-4.07E-01** | **2.36E-01** | 1.47E-01 | **-6.65E-01** | **-**6.66E-01 |
| 1.6 | VQE* | 6.54E-02 | 6.52E-02 | 2.23E-02 | 4.34E-02 | 1.09E-02 | 6.02E+02 | -4.09E-01 | 2.37E-01 | 1.47E-01 | -6.66E-01 | -6.67E-01 |
| | VQE-LD | 6.55E-02 | 6.54E-02 | 2.27E-02 | 4.31E-02 | 1.09E-02 | 6.02E+02 | -4.09E-01 | 2.37E-01 | 1.48E-01 | -6.67E-01 | -6.68E-01 |
| | VQE | 5.95E-02 | **5.94E-02** | 6.57E-02 | **6.42E-02** | 1.58E-02 | 8.94E+02 | -4.45E-01 | 3.46E-01 | 2.97E-01 | -2.92E-01 | -2.92E-01 |
| 1.8 | VQE* | 6.95E-02 | 6.97E-02 | 6.38E-02 | 4.46E-02 | -1.06E-03 | 3.98E+02 | -4.25E-01 | -1.68E-01 | -1.35E-01 | -2.79E-01 | -2.79E-01 |
| | VQE-LD | 6.96E-02 | 6.95E-02 | 6.34E-02 | 4.49E-02 | -1.31E-03 | 3.85E+02 | -4.22E-01 | -1.70E-01 | -1.37E-01 | -2.77E-01 | -2.77E-01 |
| | VQE | -1.79E-01 | -1.67E-01 | 6.22E-02 | **5.78E-02** | **7.16E-04** | 7.57E+02 | -2.95E-01 | -4.80E-02 | -7.47E-02 | -1.19E+00 | -1.19E+00 |
| 2.1 | VQE* | -1.30E-01 | -1.69E-01 | 1.14E-02 | 5.80E-02 | 6.63E-04 | 7.28E+02 | -2.92E-01 | -3.79E-02 | -6.23E-02 | -1.18E+00 | -1.18E+00 |
| | VQE-LD | -1.30E-01 | -1.69E-01 | 1.14E-02 | 5.80E-02 | 6.63E-04 | 7.28E+02 | -2.92E-01 | -3.79E-02 | -6.23E-02 | -1.18E+00 | -1.18E+00 |

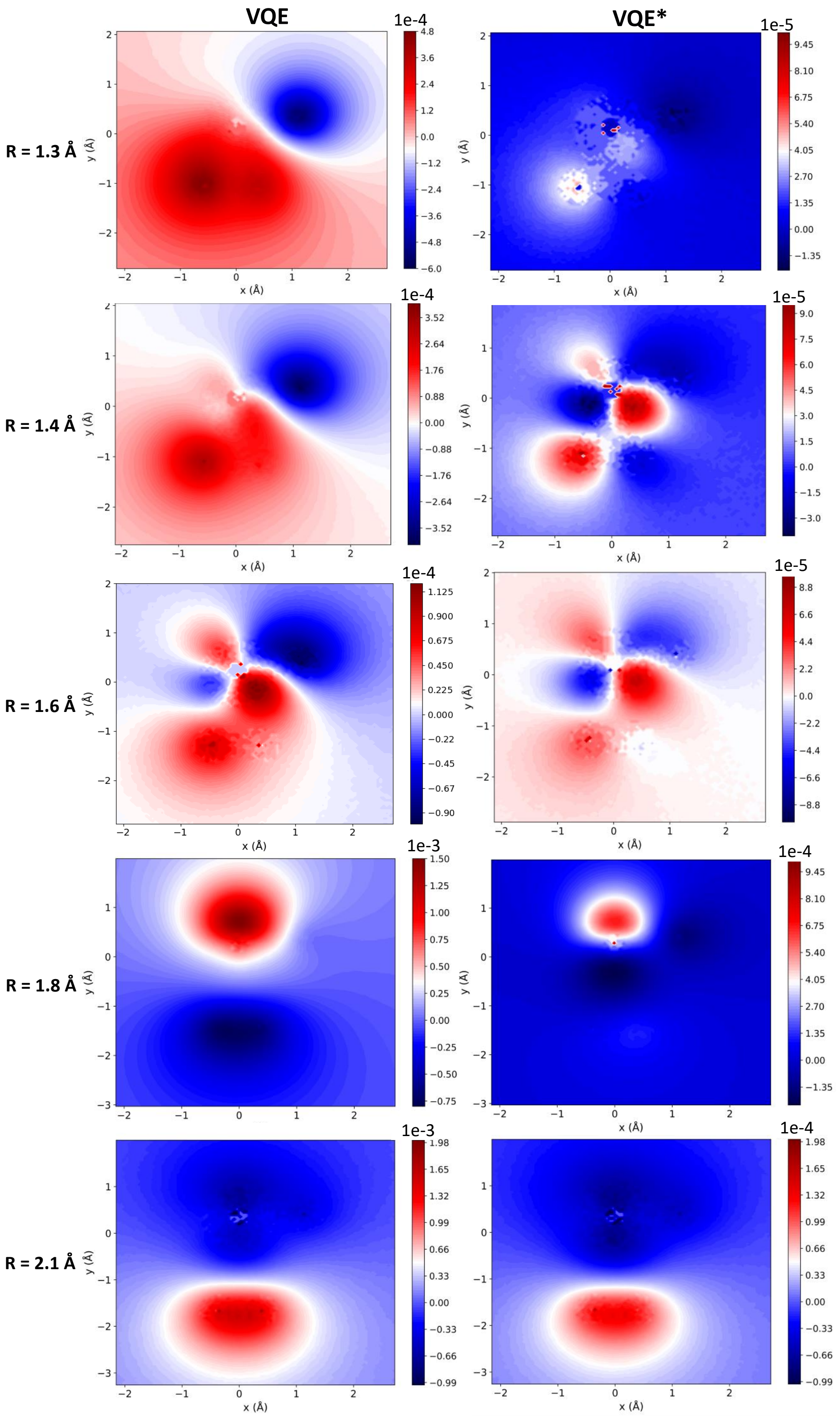


FIG. S8. Difference in electrostatic potential (in $e/a_0$) along the $x$ and $y$ axes of $CH_5^+$ dissociating geometries, obtained with VQE and VQE* methods with the k-UpCCGSD *ansatz* relative to CISD within a (4,4)-active space.

TABLE S7. Mulliken population analysis of the $CH_5^+$ dissociation geometries with VQE and VQE* compared to CASCI(4,4). The k-UpCCGSD and the (4,4)-active space were used.

| Atom | 1.3 | | | | 1.4 | | | | 1.6 | | | | 1.8 | | | | 2.1 | | | |
|---|---|---|---|---|---|---|---|---|---|---|---|---|---|---|---|---|---|---|---|---|
| | **CASCI** | **VQE** | **VQE*** | **VQE-LD** | **CASCI** | **VQE** | **VQE*** | **VQE-LD** | **CASCI** | **VQE** | **VQE*** | **VQE-LD** | **CASCI** | **VQE** | **VQE*** | **VQE-LD** | **CASCI** | **VQE** | **VQE*** | **VQE-LD** |
| **Mulliken Charges** | | | | | | | | | | | | | | | | | | | | |
| C | -0.19698 | -0.19441 | -0.19697 | -0.19697 | -0.15027 | -0.15009 | -0.15027 | -0.15028 | -0.00513 | -0.00506 | -0.00512 | -0.00513 | 0.05817 | 0.10945 | 0.06067 | 0.06072 | 0.16015 | 0.16188 | 0.16025 | 0.16026 |
| H2 | 0.23503 | 0.22923 | 0.23502 | 0.23503 | 0.22877 | 0.22838 | 0.22873 | 0.22873 | 0.22408 | 0.22398 | 0.22405 | 0.22405 | 0.23047 | 0.2304 | 0.22961 | 0.2295 | 0.24248 | 0.24238 | 0.24246 | 0.24246 |
| H3 | 0.26164 | 0.26504 | 0.26167 | 0.26167 | 0.24301 | 0.24330 | 0.24310 | 0.24310 | 0.16834 | 0.16841 | 0.16837 | 0.16837 | 0.12394 | 0.09828 | 0.12376 | 0.12387 | 0.05411 | 0.05341 | 0.0541 | 0.05409 |
| H4 | 0.26599 | 0.26865 | 0.26597 | 0.26597 | 0.24526 | 0.24535 | 0.24519 | 0.24519 | 0.16896 | 0.16896 | 0.16894 | 0.16894 | 0.12386 | 0.09855 | 0.12404 | 0.12419 | 0.05441 | 0.05372 | 0.05440 | 0.05440 |
| H5 | 0.21716 | 0.21575 | 0.21715 | 0.21715 | 0.21666 | 0.21654 | 0.21663 | 0.21663 | 0.22188 | 0.22186 | 0.22188 | 0.22188 | 0.23178 | 0.23166 | 0.23096 | 0.23085 | 0.24442 | 0.24431 | 0.2444 | 0.24440 |
| H6 | 0.21716 | 0.21575 | 0.21715 | 0.21715 | 0.21666 | 0.21654 | 0.21663 | 0.21663 | 0.22188 | 0.22186 | 0.22188 | 0.22188 | 0.23178 | 0.23166 | 0.23096 | 0.23087 | 0.24442 | 0.24431 | 0.24440 | 0.24440 |
| **Mulliken Pop** | | | | | | | | | | | | | | | | | | | | |
| C (1s) | 1.99280 | 1.99280 | 1.99280 | 1.99280 | 1.99302 | 1.99302 | 1.99302 | 1.99302 | 1.99369 | 1.99369 | 1.99369 | 1.99369 | 1.99411 | 1.99413 | 1.99410 | 1.99410 | 1.99442 | 1.99442 | 1.99442 | 1.99442 |
| C (2s) | 1.30083 | 1.30083 | 1.30083 | 1.30083 | 1.31201 | 1.31202 | 1.31202 | 1.31202 | 1.34835 | 1.34834 | 1.34835 | 1.34835 | 1.37297 | 1.37505 | 1.37173 | 1.37161 | 1.39454 | 1.39436 | 1.39449 | 1.39449 |
| C (2px) | 1.12726 | 1.12476 | 1.12725 | 1.12725 | 1.13485 | 1.13469 | 1.13486 | 1.13486 | 1.14891 | 1.14889 | 1.1489 | 1.1489 | 1.15819 | 1.1582 | 1.15819 | 1.15819 | 1.16995 | 1.16995 | 1.16995 | 1.16995 |
| C(2py) | 0.61459 | 0.61450 | 0.61458 | 0.61458 | 0.55058 | 0.55053 | 0.55055 | 0.55055 | 0.35656 | 0.35649 | 0.35656 | 0.35656 | 0.2562 | 0.20322 | 0.25536 | 0.25543 | 0.11202 | 0.11067 | 0.11219 | 0.11218 |
| C (2pz) | 1.16151 | 1.16152 | 1.16151 | 1.16151 | 1.15982 | 1.15983 | 1.15982 | 1.15982 | 1.15762 | 1.15765 | 1.15762 | 1.15762 | 1.15997 | 1.15995 | 1.15995 | 1.15995 | 1.16871 | 1.16872 | 1.1687 | 1.1687 |
| H2 (1s) | 0.76495 | 0.77077 | 0.76498 | 0.76497 | 0.77122 | 0.77162 | 0.77127 | 0.77127 | 0.77591 | 0.77602 | 0.77595 | 0.77595 | 0.76952 | 0.76960 | 0.77039 | 0.7705 | 0.75752 | 0.75762 | 0.75754 | 0.75754 |
| H3 (1s) | 0.73837 | 0.73496 | 0.73833 | 0.73833 | 0.7570 | 0.75670 | 0.75690 | 0.75690 | 0.83167 | 0.83159 | 0.83163 | 0.83163 | 0.87625 | 0.90172 | 0.87624 | 0.87613 | 0.94599 | 0.94659 | 0.9459 | 0.94591 |
| H4 (1s) | 0.73402 | 0.73135 | 0.73403 | 0.73403 | 0.75475 | 0.75465 | 0.75481 | 0.75481 | 0.83104 | 0.83104 | 0.83106 | 0.83106 | 0.87632 | 0.90145 | 0.87596 | 0.87581 | 0.94569 | 0.94628 | 0.9456 | 0.9456 |
| H5 (1s) | 0.78284 | 0.78425 | 0.78285 | 0.78285 | 0.78338 | 0.78346 | 0.78337 | 0.78337 | 0.77812 | 0.77814 | 0.77812 | 0.77812 | 0.76823 | 0.76834 | 0.76904 | 0.76915 | 0.75558 | 0.75569 | 0.7556 | 0.7556 |
| H6 (1s) | 0.78284 | 0.78425 | 0.78285 | 0.78285 | 0.78338 | 0.78346 | 0.78337 | 0.78337 | 0.77812 | 0.77814 | 0.77812 | 0.77812 | 0.76823 | 0.76834 | 0.76904 | 0.76913 | 0.75558 | 0.75569 | 0.7556 | 0.7556 |

TABLE S8. Differences in Mulliken charges (in $e$) obtained with VQE, VQE* and VQE-LD methods, using the k-UpCCGSD *ansatz*, relative to CISD within the same (4,4)-active space. Bold values mark cases where VQE yields smaller errors than both VQE* and VQE-LD.

| **R (Å)** | **Method** | **C1** | **H2** | **H3** | **H4** | **H5** | **H6** |
|---|---|---|---|---|---|---|---|
| 1.3 | VQE | 2.58E-3 | -5.82E-3 | 3.41E-3 | 2.67E-3 | -1.41E-3 | -1.41E-3 |
| | VQE* | 2.00E-5 | -3.00E-5 | 4.00E-5 | -1.00E-5 | -1.00E-5 | -1.00E-5 |
| | VQE-LD | 2.00E-5 | -2.00E-5 | 4.00E-5 | -1.00E-5 | -1.00E-5 | -1.00E-5 |
| 1.4 | VQE | 1.90E-4 | -4.00E-4 | 3.00E-4 | 1.00E-4 | -8.00E-5 | -8.00E-5 |
| | VQE* | 1.00E-5 | -5.00E-5 | 1.00E-4 | -6.00E-5 | 1.00E-5 | 1.00E-5 |
| | VQE-LD | 0.00 | -5.00E-5 | 1.00E-4 | -6.00E-5 | 1.00E-5 | 1.00E-5 |
| 1.6 | VQE | 8.00E-5 | -1.10E-4 | 8.00E-5 | **0.00** | -2.00E-5 | -2.00E-5 |
| | VQE* | 2.00E-5 | -4.00E-5 | 4.00E-5 | -2.00E-5 | 0.00 | 0.00 |
| | VQE-LD | 1.00E-5 | -4.00E-5 | 4.00E-5 | -2.00E-5 | 0.00 | 0.00 |
| 1.8 | VQE | 5.09E-2 | -2.55E-2 | -2.55E-2 | -2.51E-2 | **-1.10E-4** | **-1.10E-4** |
| | VQE* | 2.11E-3 | 1.00E-5 | 1.00E-5 | 3.60E-4 | -8.10E-4 | -8.10E-4 |
| | VQE-LD | 2.16E-3 | -9.80E-4 | 1.20E-4 | 5.10E-4 | -9.20E-4 | -9.00E-4 |
| 2.1 | VQE | 1.52E-3 | -1.00E-4 | -6.00E-4 | -5.90E-4 | -1.10E-4 | -1.10E-4 |
| | VQE* | -1.10E-4 | -2.00E-5 | 9.00E-5 | 9.00E-5 | -2.00E-5 | -2.00E-5 |
| | VQE-LD | -1.00E-4 | -2.00E-5 | 8.00E-5 | 9.00E-5 | -2.00E-5 | -2.00E-5 |

TABLE S9. Differences in Mulliken populations (in $e$) obtained with VQE, VQE*, and VQE-LD methods, using the k-UpCCGSD *ansatz*, relative CISD within the same (4,4)-active space. Bold values mark cases where VQE yields smaller errors than both VQE* and VQE-LD.

| **R (Å)** | **Method** | **C(1s)** | **C(2s)** | **C(2px)** | **C(2py)** | **C(2pz)** | **H2 (1s)** | **H3 (1s)** | **H4 (1s)** | **H5 (1s)** | **H6 (1s)** |
|---|---|---|---|---|---|---|---|---|---|---|---|
| 1.3 | VQE | 0.00 | 0.00 | -2.50E-3 | -9.00E-5 | 1.00E-5 | 5.82E-3 | -3.41E-3 | -2.67E-3 | 1.41E-3 | 1.41E-3 |
| | VQE* | 0.00 | 0.00 | -1.00E-5 | -1.00E-5 | 0.00 | 3.00E-5 | -4.00E-5 | 1.00E-5 | 1.00E-5 | 1.00E-5 |
| | VQE-LD | 0.00 | 0.00 | -1.00E-5 | -1.00E-5 | 0.00 | 2.00E-5 | -4.00E-5 | 1.00E-5 | 1.00E-5 | 1.00E-5 |
| 1.4 | VQE | 0.00 | 1.00E-5 | -1.60E-4 | -5.00E-5 | 1.00E-5 | 4.00E-4 | -3.00E-4 | -1.00E-4 | 8.00E-5 | 8.00E-5 |
| | VQE* | 0.00 | 1.00E-5 | 1.00E-5 | -3.00E-5 | 0.00 | 5.00E-5 | -1.00E-4 | 6.00E-5 | -1.00E-5 | -1.00E-5 |
| | VQE-LD | 0.00 | 1.00E-5 | 1.00E-5 | -3.00E-5 | 0.00 | 5.00E-5 | -1.00E-4 | 6.00E-5 | -1.00E-5 | -1.00E-5 |
| 1.6 | VQE | 0.00 | -1.00E-5 | -2.00E-5 | -7.00E-5 | 3.00E-5 | 1.10E-4 | -8.00E-5 | 6.00E-5 | 2.00E-5 | 2.00E-5 |
| | VQE* | 0.00 | 0.00 | -1.00E-5 | 0.00 | 0.00 | 4.00E-5 | -4.00E-5 | 2.00E-5 | 0.00 | 0.00 |
| | VQE-LD | 0.00 | 0.00 | -1.00E-5 | 0.00 | 0.00 | 4.00E-5 | -4.00E-5 | 2.00E-5 | 0.00 | 0.00 |
| 1.8 | VQE | 2.00E-5 | 2.08E-3 | 1.00E-5 | -5.30E-2 | -2.00E-5 | 8.00E-5 | 2.55E-2 | 2.51E-2 | **1.10E-4** | **1.10E-4** |
| | VQE* | -1.00E-5 | -1.24E-3 | 0.00 | -8.40E-4 | -2.00E-5 | 8.70E-4 | -1.00E-5 | -3.60E-4 | 8.10E-4 | 8.10E-4 |
| | VQE-LD | -1.00E-5 | -1.36E-3 | 0.00 | -7.70E-4 | -2.00E-5 | 9.80E-4 | -1.20E-5 | -5.10E-4 | 9.20E-4 | 8.00E-4 |
| 2.1 | VQE | 0.00 | -1.80E-4 | 0.00 | -1.35E-3 | 1.00E-5 | 1.00E-4 | 6.00E-4 | 5.90E-4 | 1.10E-4 | 1.10E-4 |
| | VQE* | 0.00 | -5.00E-5 | 0.00 | 1.70E-4 | -1.00E-5 | 2.00E-5 | -9.00E-5 | -9.00E-5 | 2.00E-5 | 2.00E-5 |
| | VQE-LD | 0.00 | -5.00E-5 | 0.00 | 1.60E-4 | -1.00E-5 | 2.00E-5 | -7.00E-5 | -9.00E-5 | 2.00E-5 | 2.00E-5 |

TABLE S10. Energies (in $E_h$) and corresponding numbers of optimization steps obtained with VQE-LD for dissociating $CH_5^+$ geometries at $R = 1.3$ and 1.4 Å. Calculations were performed with the GateFabric *ansatz* in a (2,2)-active space for different convergence rates, $f$.

| $f$ | **1.3** | | **1.4** | |
|---|---|---|---|---|
| | **Energy** | **Steps** | **Energy** | **Steps** |
| 0.1 | -39.917589475 | 313 | -39.917619351 | 502 |
| 0.3 | -39.917589475 | 347 | -39.917619351 | 249 |
| 0.4 | -39.917589475 | 175 | -39.917619351 | 252 |
| 0.5 | -39.917589475 | 168 | -39.917619351 | 244 |
| 0.6 | 39.917589475 | 113 | -39.917619351 | 178 |
| 0.7 | -39.917589475 | 68 | -39.917619351 | 192 |
| 0.8 | -39.917589475 | 760 | -39.917619351 | 149 |
| 0.9 | -39.917589475 | 71 | -39.917619351 | 67 |
| 1 | -39.496312631 | 6 | -39.842484498 | 7 |

TABLE S11. CISD(2,2) and full-CI reference energie (in $E_\mathrm{h}$), together with the corresponding errors of VQE, VQE, and VQE-LD*, obtained using the GateFabric *ansatz* in a (2,2)-active space for $CH_5^+$ structures at $R = 1.3$ and $R = 1.4$ Å.

| R (Å) | Energy CISD ($E_\mathrm{h}$) | Error | | | Energy FCI ($E_\mathrm{h}$) | Error | | |
|---|---|---|---|---|---|---|---|---|
| | | VQE | VQE* | VQE-LD | | VQE | VQE* | VQE-LD |
| 1.3 | -39.91925976 | 2.60E-1 | -7.50E-8 | -7.45E-8 | -40.01161394 | 2.615E-01 | 1.670E-03 | 1.670E-03 |
| 1.4 | -39.91888589 | 1.45E-1 | -5.10E-8 | -5.10E-8 | -40.01134578 | 1.461E-01 | 1.267E-03 | 1.267E-03 |

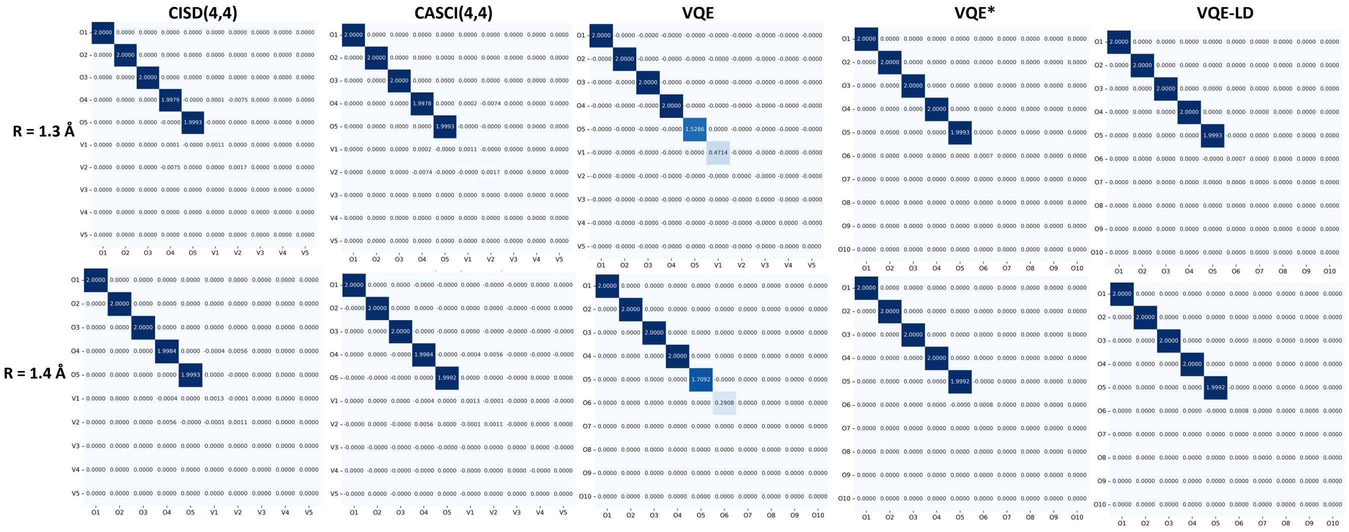


FIG. S9. 1-RDM for intermediate $CH_5^+$ structures at $R = 1.3$ and $R = 1.4$ Å, obtained with CISD(4,4), CASCI(4,4), and with VQE, VQE* and VQE-LD using the GateFabric *ansatz* in a (2,2)-active space. O1 to O5 refer to the occupied orbitals, while V1 to V5 are the virtual orbitals.

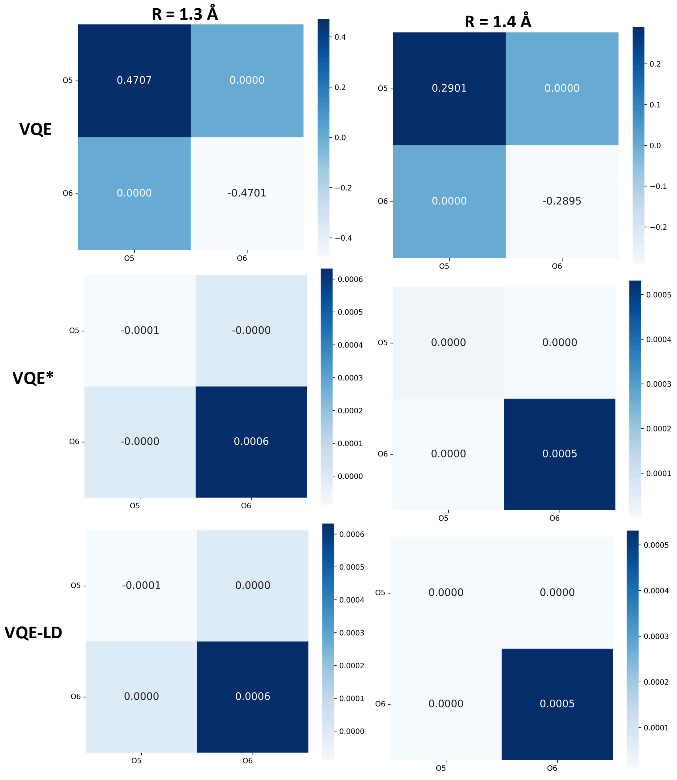


FIG. S10. Difference in the 1-RDM obtained with VQE and VQE*, using the GateFabric *ansatz* and the (2,2)-active space, relative to CASCI(4,4). VQE-LD yields the same 1-RDM differences as VQE*. O4 and O5 refer to the last two occupied orbitals, while V1 and V2 are the first two virtual orbitals. Here, O5 and V1 are the active orbitals.

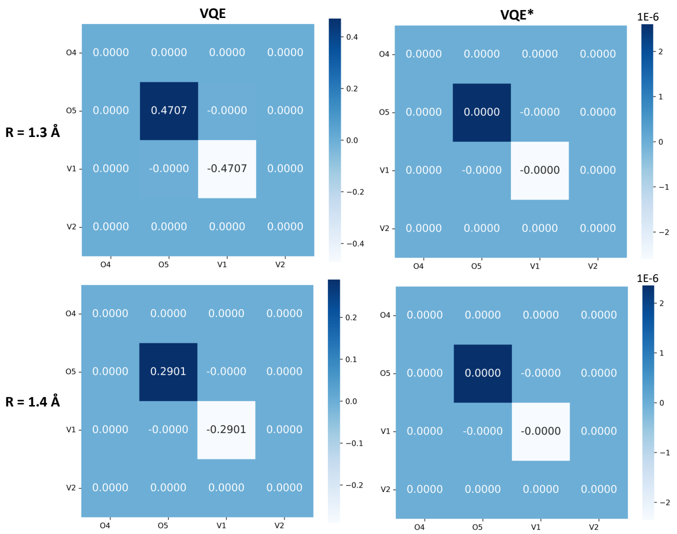


FIG. S11. Difference in 1-RDM obtained with VQE and VQE* compared to CISD. The VQE-LD presented the same result as the VQE*. The GateFabri (2,2)-active space *ansatz* was used. O4 and O5 refer to the last two occupied orbitals, while V1 and V2 are the first two virtual orbitals. Here, O5 and V1 are the active orbitals.

TABLE S12. Total dipole moment ($\mu$) and corresponding error ($\Delta\mu = \mu_{\text{method}} - \mu_{\text{CISD}}$), in Debye (D), computed for $CH_5^+$ structures with $R = 1.3$ and $R = 1.4$ Åusing the GateFabric *ansatz* within a (2,2)-active space.

| **R** (Å) | $\mu$ (D) | | | | $\Delta\mu$ (D) | | |
|---|---|---|---|---|---|---|---|
| | **CISD** | **VQE** | **VQE*** | **VQE-LD** | **VQE** | **VQE*** | **VQE-LD** |
| 1.3 | 1.955 | 0.532 | 1.955 | 1.955 | -1.420 | 0.00 | 0.00 |
| 1.4 | 1.839 | 0.961 | 1.839 | 1.839 | -0.878 | 0.00 | 0.00 |

TABLE S13. Mulliken charges and populations (in atomic charge units) obtained by VQE, VQE*, VQE-LD (with GateFabric *ansatz*) and CISD, using (2,2)-active space.

| **Atom** | **1.3** | | | | **1.4** | | | |
|---|---|---|---|---|---|---|---|---|
| | **CISD** | **VQE** | **VQE*** | **VQE-LD** | **CISD** | **VQE** | **VQE*** | **VQE-LD** |
| **Mulliken Charges** | | | | | | | | |
| C | -0.19645 | -0.24056 | -0.19645 | -0.19645 | -0.14961 | -0.18735 | -0.14962 | -0.14962 |
| H2 | 0.23150 | 0.23057 | 0.23150 | 0.23150 | 0.22617 | 0.22512 | 0.22617 | 0.22617 |
| H3 | 0.26423 | 0.2065 | 0.26423 | 0.26423 | 0.24479 | 0.20610 | 0.24479 | 0.24479 |
| H4 | 0.26811 | 0.17815 | 0.26811 | 0.26811 | 0.24665 | 0.20440 | 0.24665 | 0.24665 |
| H5 | 0.21631 | 0.31267 | 0.21631 | 0.21631 | 0.21600 | 0.27587 | 0.21600 | 0.21601 |
| H6 | 0.21631 | 0.31267 | 0.21631 | 0.21631 | 0.21600 | 0.27587 | 0.21600 | 0.21600 |
| **Mulliken Populations** | | | | | | | | |
| C (1s) | 1.99280 | 1.99327 | 1.99280 | 1.99280 | 1.99302 | 1.99327 | 1.99302 | 1.99302 |
| C (2s) | 1.30080 | 1.33907 | 1.30080 | 1.3008 | 1.31198 | 1.33221 | 1.31198 | 1.31198 |
| C (2px) | 1.12706 | 1.12835 | 1.12706 | 1.12706 | 1.13459 | 1.13492 | 1.13459 | 1.13459 |
| C(2py) | 0.61428 | 0.89183 | 0.61428 | 0.61428 | 0.55022 | 0.73542 | 0.55022 | 0.55022 |
| C (2pz) | 1.16151 | 0.88803 | 1.16151 | 1.16151 | 1.15981 | 0.99153 | 1.15981 | 1.15981 |
| H2 (1s) | 0.76850 | 0.76943 | 0.76850 | 0.76850 | 0.77383 | 0.77488 | 0.77383 | 0.77383 |
| H3 (1s) | 0.73577 | 0.79350 | 0.73577 | 0.73577 | 0.75521 | 0.7939 | 0.75521 | 0.75521 |
| H4 (1s) | 0.73189 | 0.82185 | 0.73189 | 0.73189 | 0.75335 | 0.79560 | 0.75335 | 0.75335 |
| H5 (1s) | 0.78369 | 0.68733 | 0.78369 | 0.78369 | 0.78400 | 0.72413 | 0.78399 | 0.78399 |
| H6 (1s) | 0.78369 | 0.68733 | 0.78369 | 0.78369 | 0.78400 | 0.72413 | 0.78400 | 0.78400 |

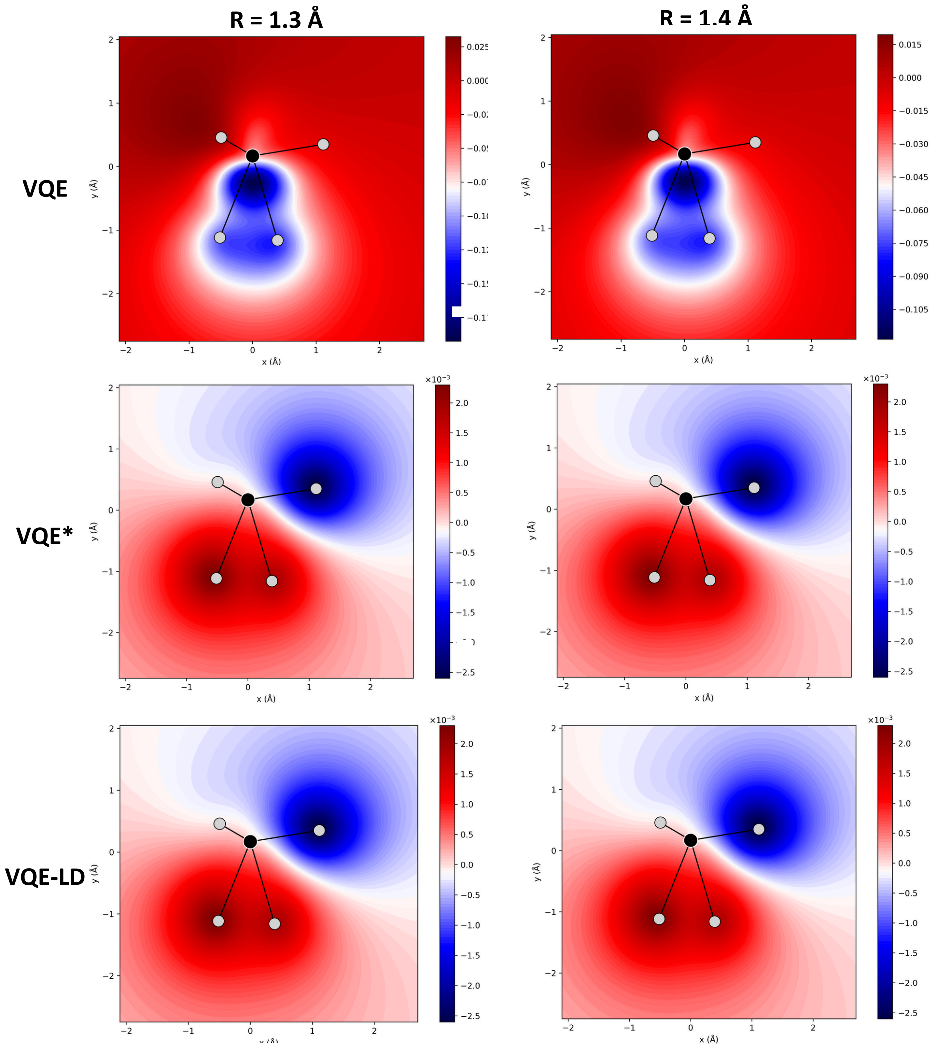


FIG. S12. Differences in electrostatic potential (in $e/a_0^3$) along the $x$ and $y$ axes obtained with VQE, VQE* and VQE-LD with GateFabric relative to CASCI(4,4) within the same (2,2)-active space.

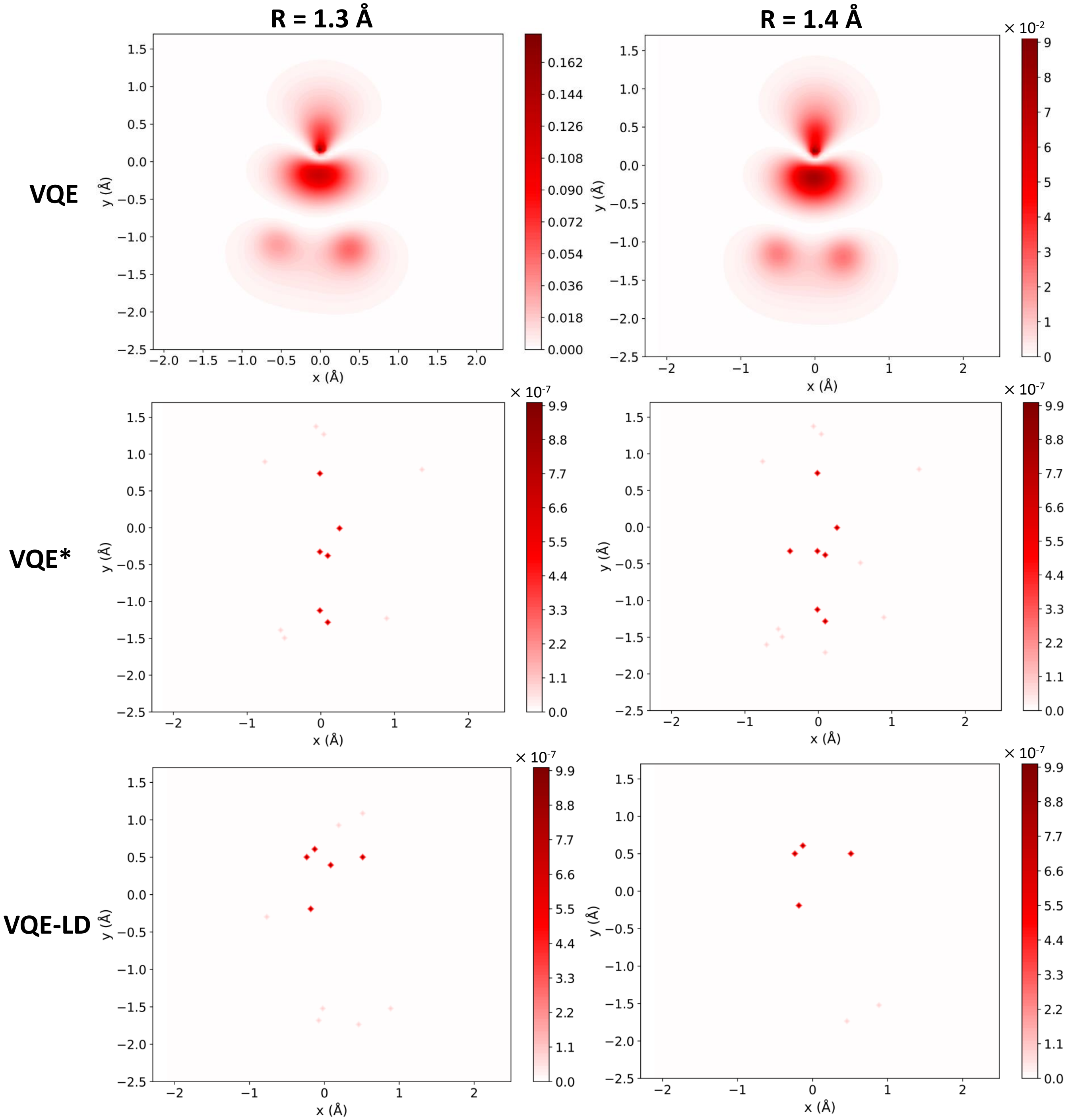


FIG. S13. Differences in electron density (in $e/a_0^3$) along the $x$ and $y$ axes obtained with VQE, VQE* and VQE-LD with GateFabric relative to CISD within the same (2,2)-active space.
