# Supplementary material for: Improving Energy and Molecular Properties by Convergence of the One‐Particle Reduced Density Matrix in Variational Quantum Eigensolvers (VQE)
Source: J Comput Chem. 2026 Jan 5;47(1):e70289. doi: 10.1002/jcc.70289 (PMC12766880; doi:10.1002/jcc.70289)
Supplement: Supplementary file 2 — Data S2: jcc70289‐sup‐0002‐Supinfo.zip. [file JCC-47-0-s002.zip › fig/met/vqe.pdf]

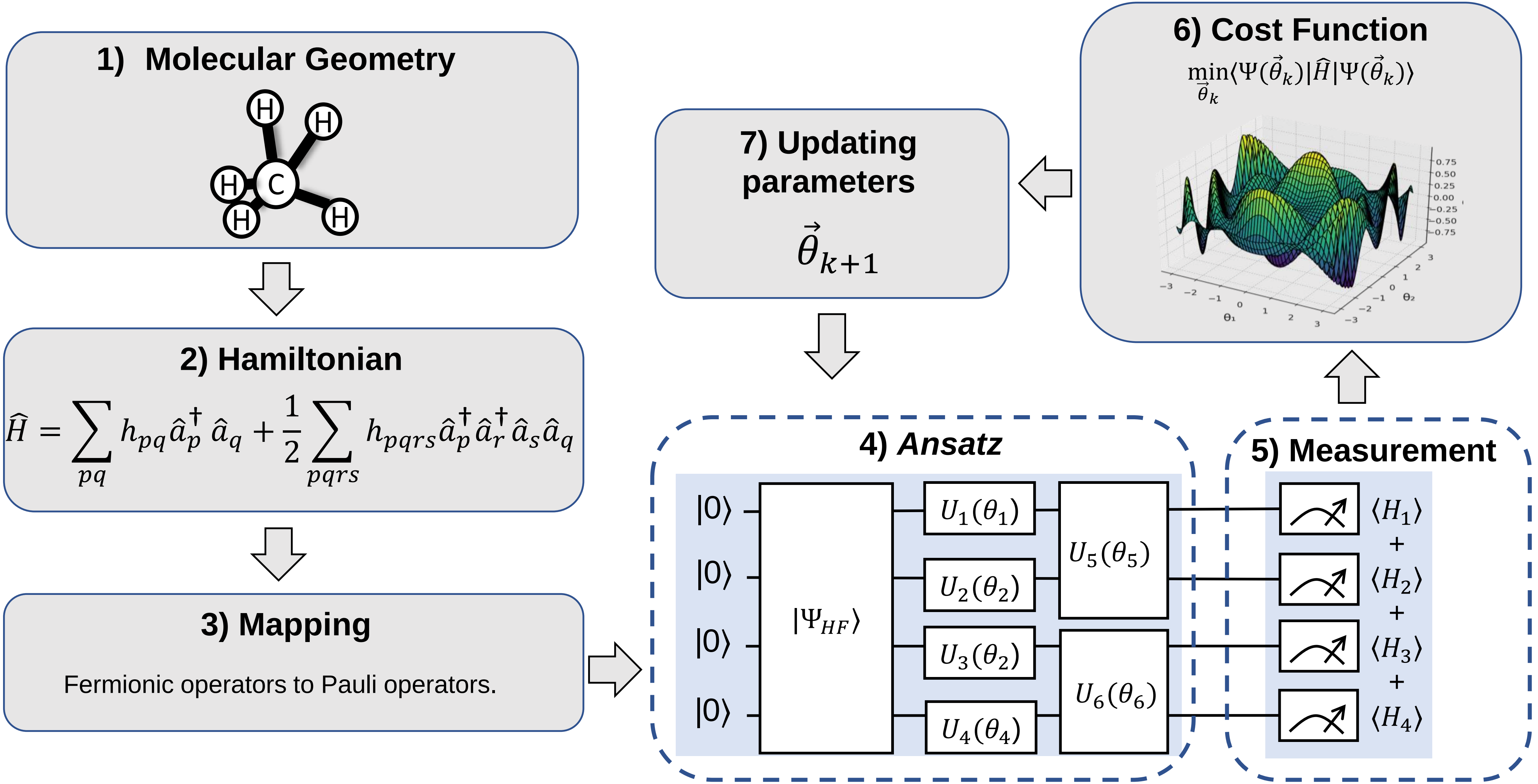

1) Molecular Geometry
H
C
2) Hamiltonian
$\hat{H} = \sum_{pq} h_{pq} \hat{a}_p^\dagger \hat{a}_q + \frac{1}{2} \sum_{pqrs} h_{pqrs} \hat{a}_p^\dagger \hat{a}_r^\dagger \hat{a}_s \hat{a}_q$
3) Mapping
Fermionic operators to Pauli operators.
4) Ansatz
$|0\rangle$
$|\Psi_{HF}\rangle$
$U_1(\theta_1)$
$U_2(\theta_2)$
$U_3(\theta_2)$
$U_4(\theta_4)$
$U_5(\theta_5)$
$U_6(\theta_6)$
5) Measurement
$\langle H_1 \rangle + \langle H_2 \rangle + \langle H_3 \rangle + \langle H_4 \rangle$
6) Cost Function
$\min_{\vec{\theta}_k} \langle \Psi(\vec{\theta}_k) | \hat{H} | \Psi(\vec{\theta}_k) \rangle$
θ₁
θ₂
7) Updating parameters
$\vec{\theta}_{k+1}$
