# Supplementary figures and images for: Improving Energy and Molecular Properties by Convergence of the One‐Particle Reduced Density Matrix in Variational Quantum Eigensolvers (VQE)
Source: J Comput Chem. 2026 Jan 5;47(1):e70289. doi: 10.1002/jcc.70289 (PMC12766880; doi:10.1002/jcc.70289)

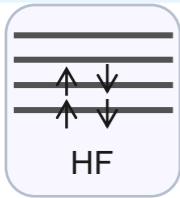

$$\gamma_{\mu\nu}^{\text{HF}}$$

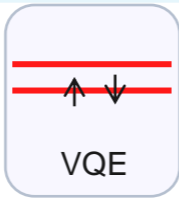

$$\gamma_{\mu\nu}^{\text{VQE}}$$

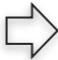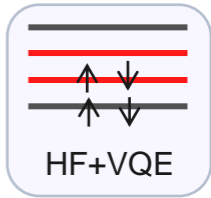

$$\gamma_{\mu\nu}$$

Supplement: Supplementary file 2 — Data S2: jcc70289‐sup‐0002‐Supinfo.zip. [file JCC-47-0-s002.zip › fig/met/dpq.pdf]

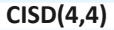

Supplement: Supplementary file 2 — Data S2: jcc70289‐sup‐0002‐Supinfo.zip. [file JCC-47-0-s002.zip › rdm1.pdf]

**R = 1.3 Å**

**VQE**

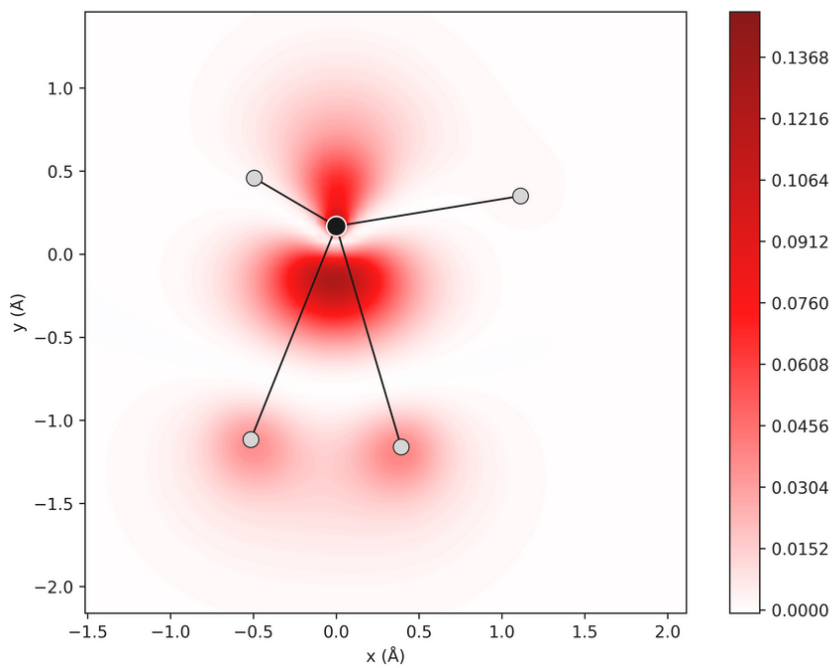

**R = 1.4 Å**

**VQE**

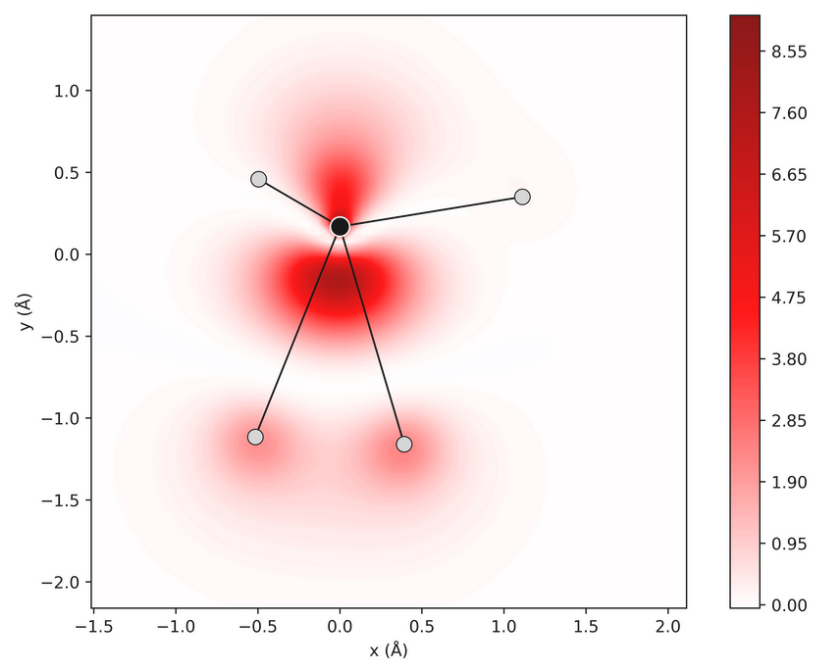

**VQE\***

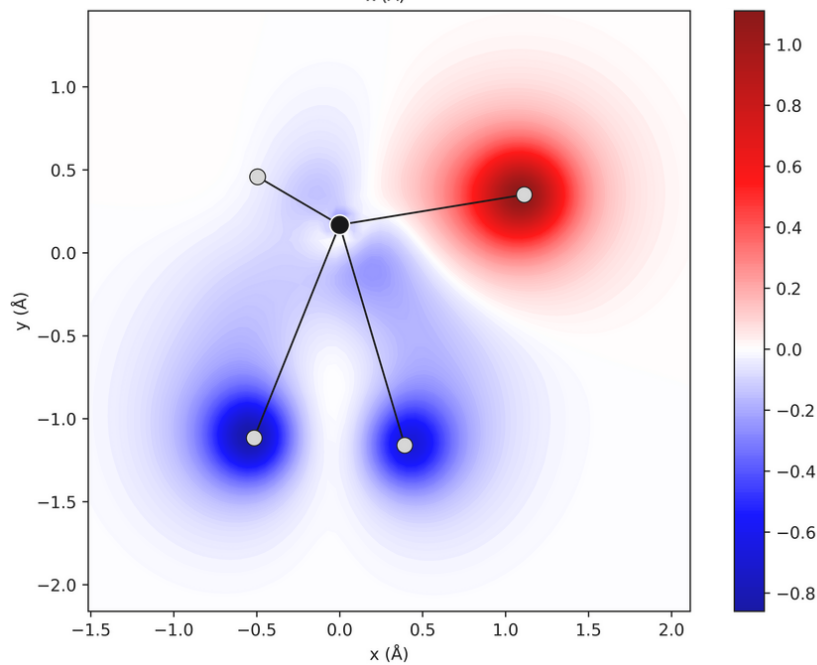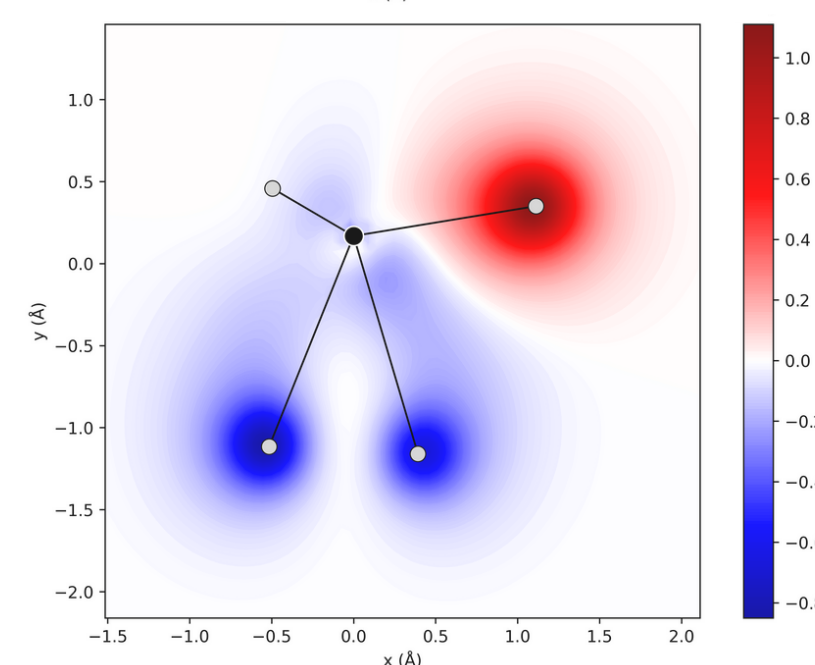

**VQE-LD**

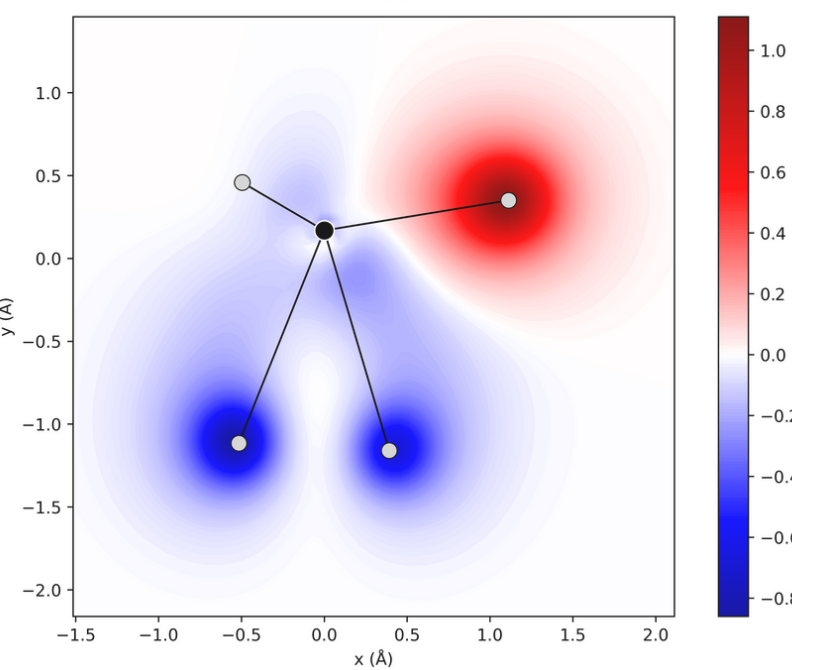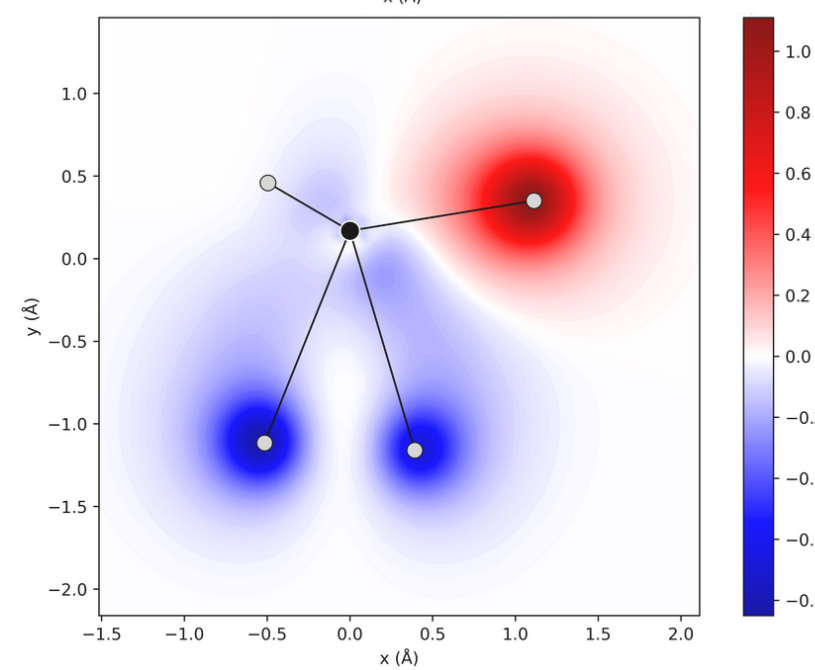

Supplement: Supplementary file 2 — Data S2: jcc70289‐sup‐0002‐Supinfo.zip. [file JCC-47-0-s002.zip › fig/dens-gf-as2-cas(4,4).pdf]

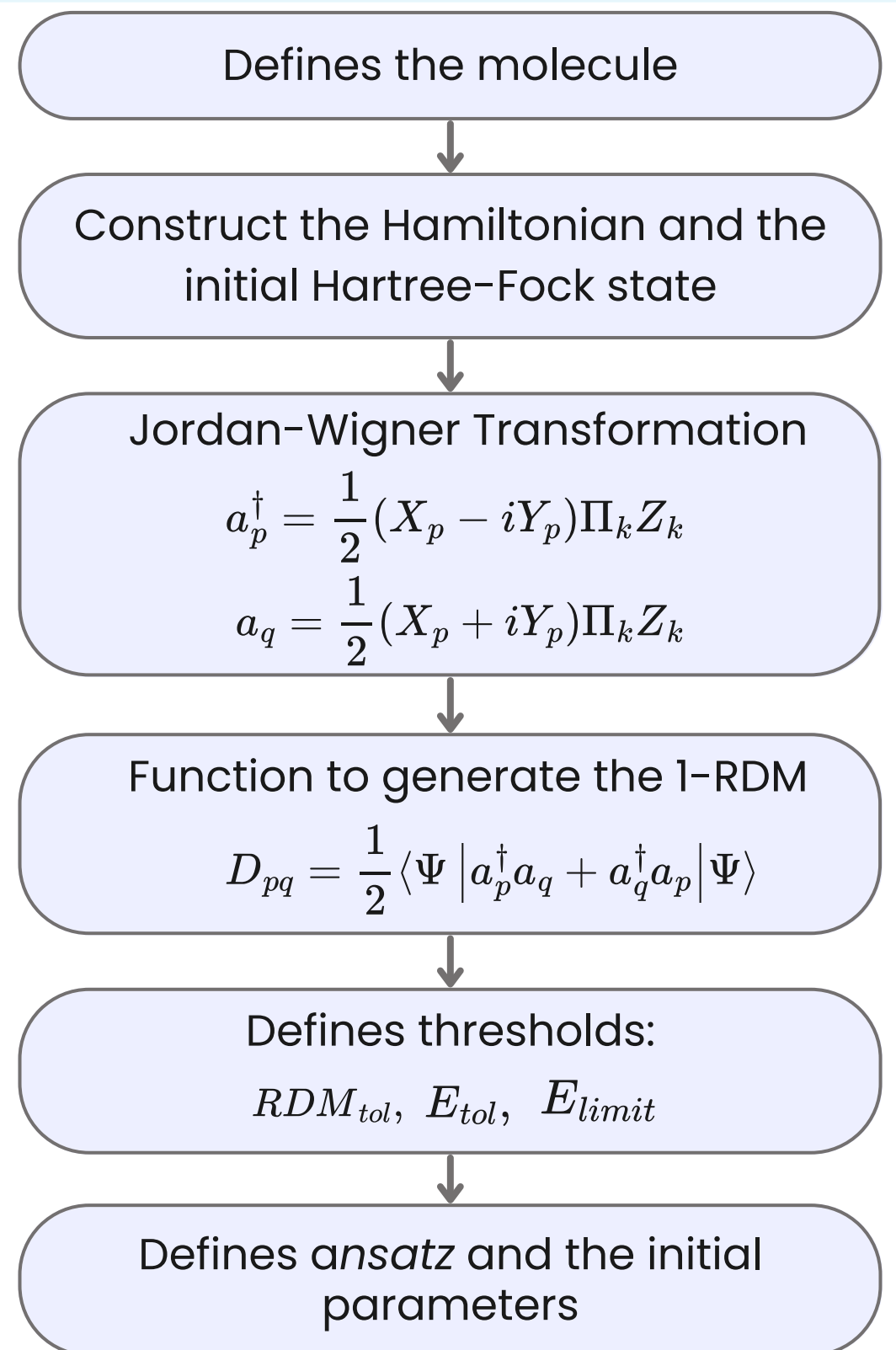

## Phase 2

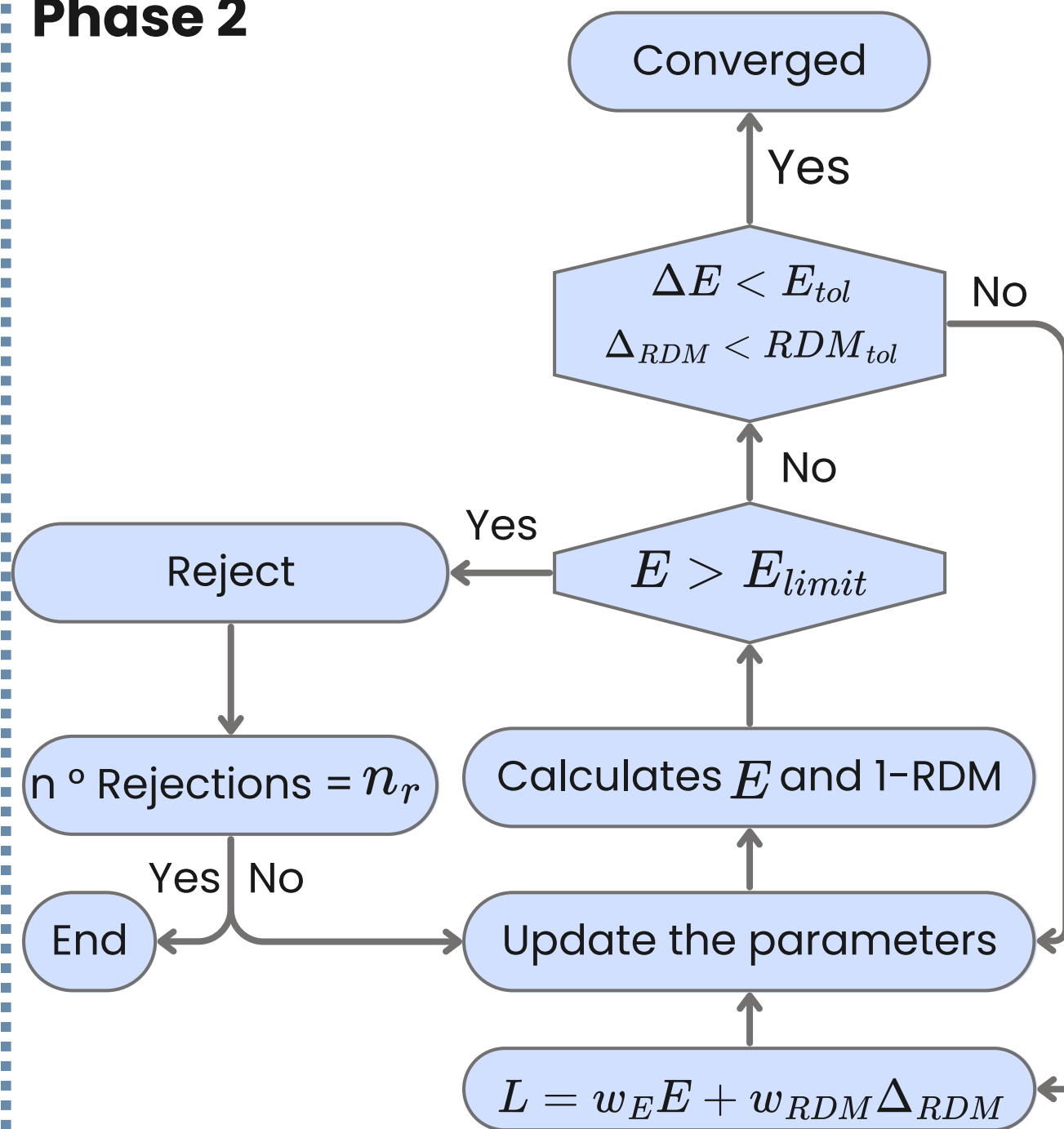

## Phase 1

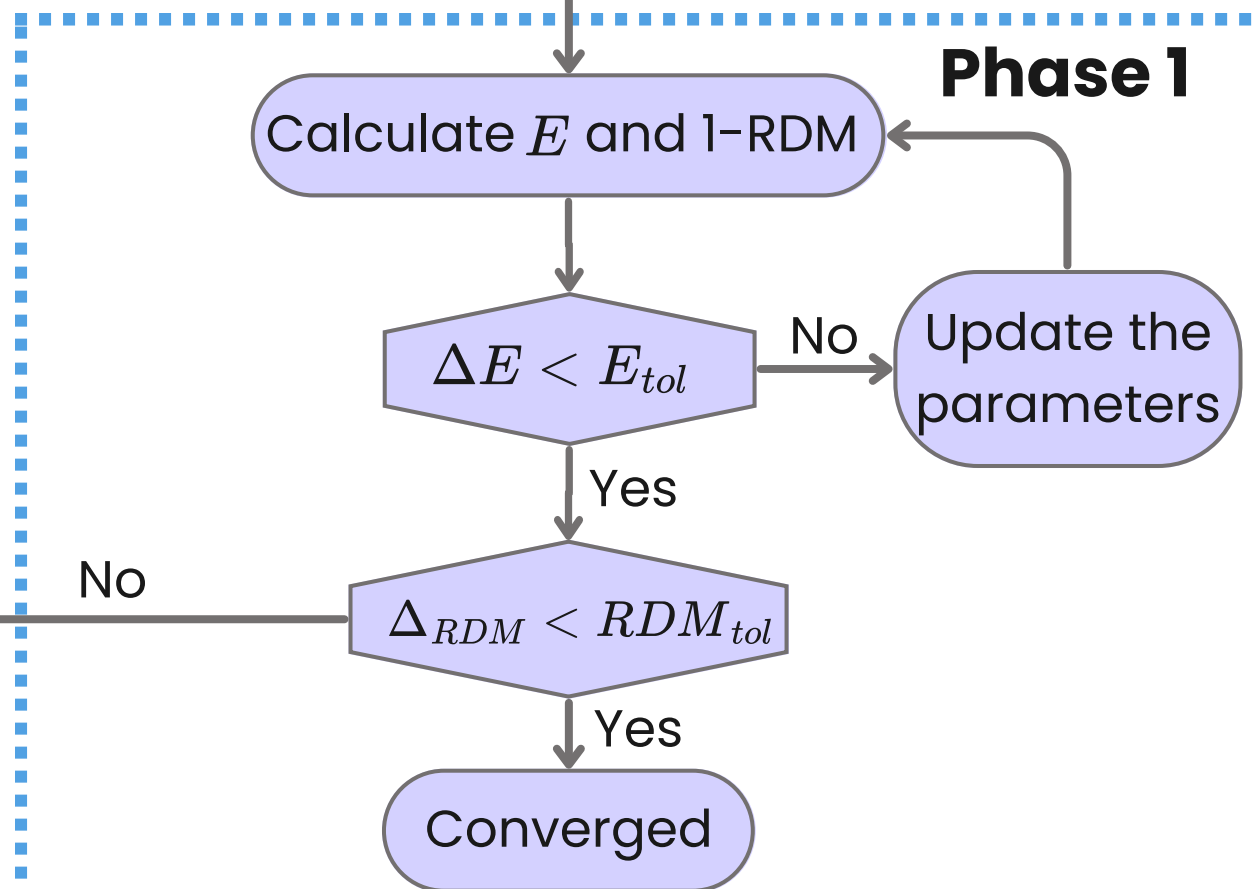

Supplement: Supplementary file 2 — Data S2: jcc70289‐sup‐0002‐Supinfo.zip. [file JCC-47-0-s002.zip › fig/met/fluxograma_rdm1_conv.pdf]

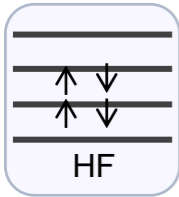

$$D_{pq}^{\text{HF}}$$

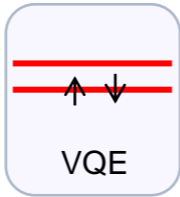

$$D_{pq}^{\text{VQE}}$$

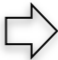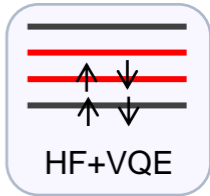

$$D_{pq}$$

Supplement: Supplementary file 2 — Data S2: jcc70289‐sup‐0002‐Supinfo.zip. [file JCC-47-0-s002.zip › fig/met/dpq-mo.pdf]

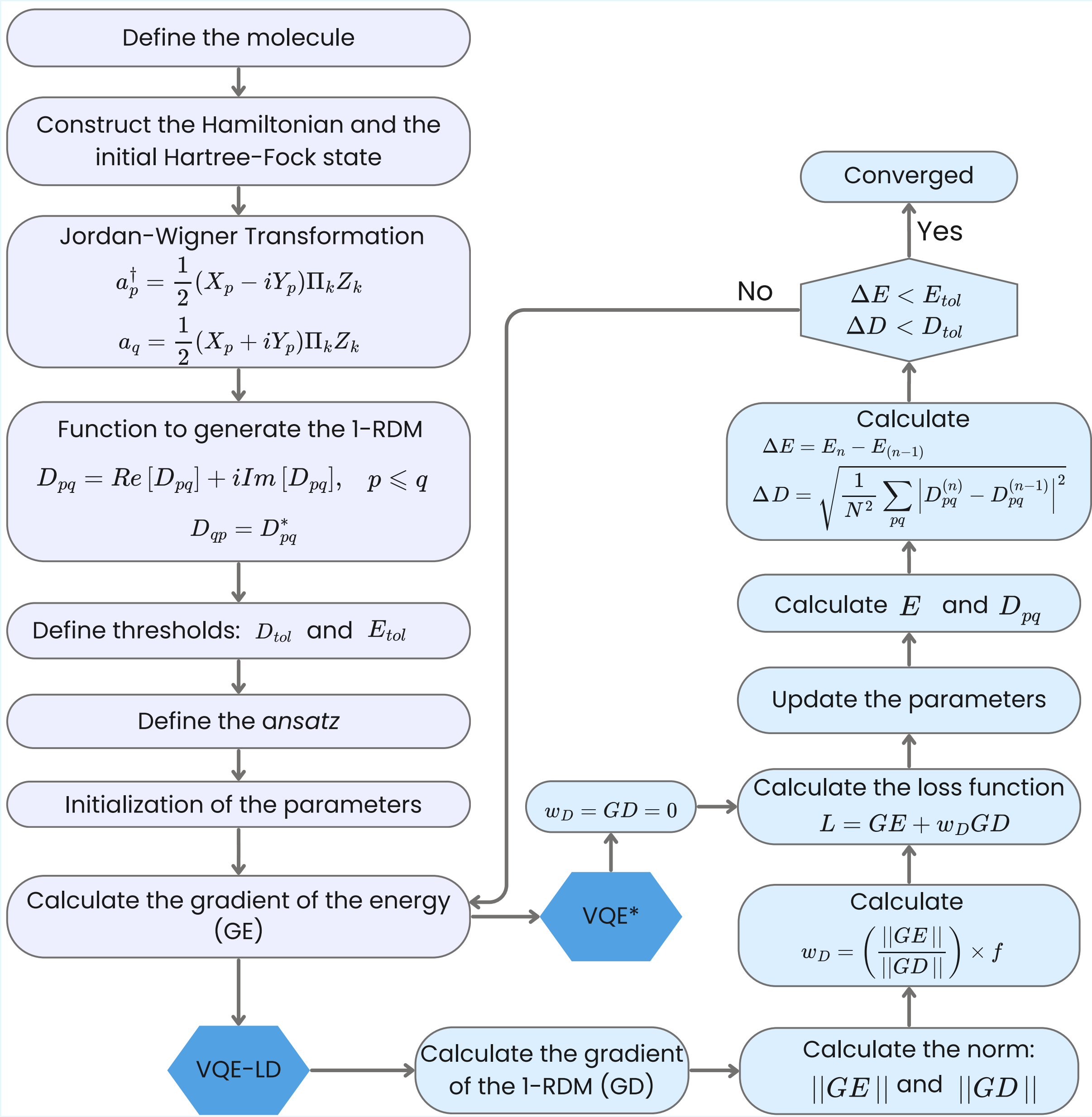

Supplement: Supplementary file 2 — Data S2: jcc70289‐sup‐0002‐Supinfo.zip. [file JCC-47-0-s002.zip › fig/met/fluxograma_rdm1_conv_new.pdf]

$R = 1.3 \text{ \AA}$

$R = 1.4 \text{ \AA}$

$R = 1.6 \text{ \AA}$

$R = 1.8 \text{ \AA}$

$R = 2.1 \text{ \AA}$

VQE

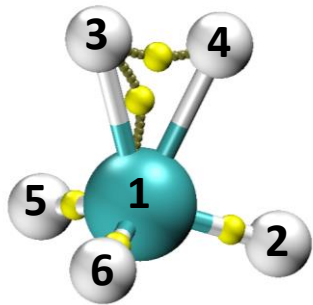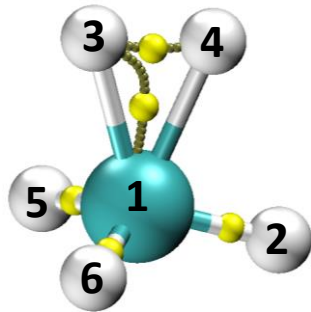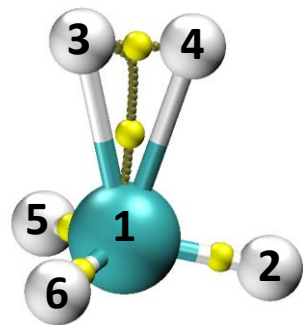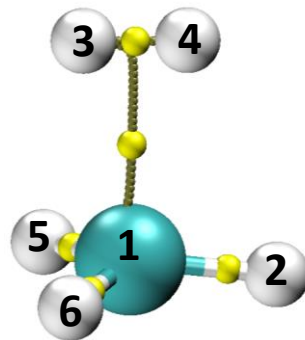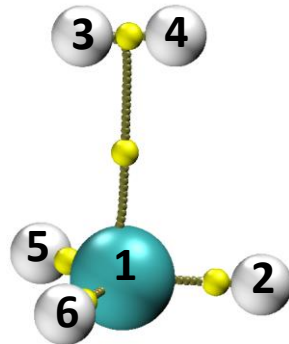

CISD

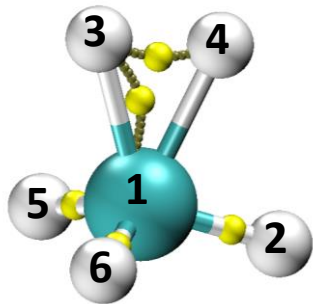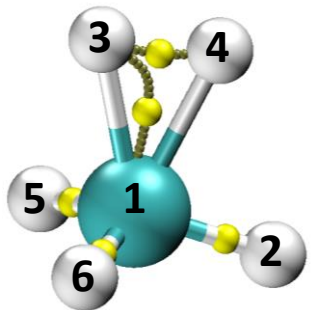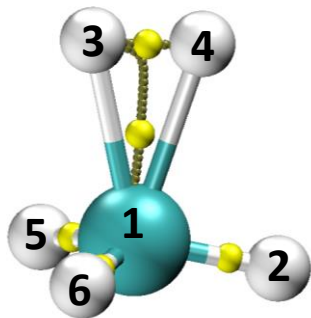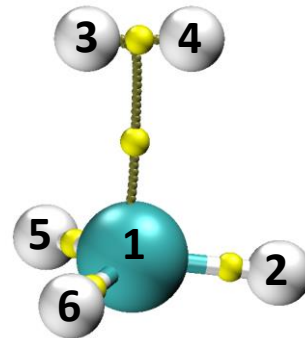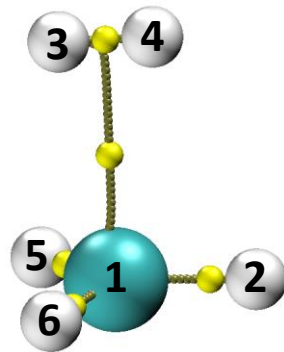

Supplement: Supplementary file 2 — Data S2: jcc70289‐sup‐0002‐Supinfo.zip. [file JCC-47-0-s002.zip › fig/results/critc2.pdf]

**VQE**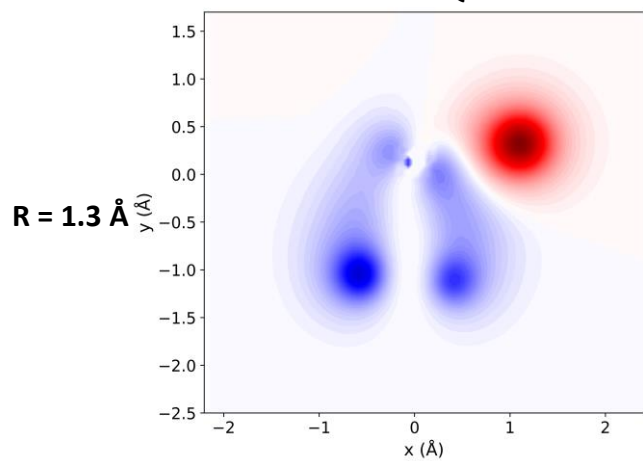**VQE\***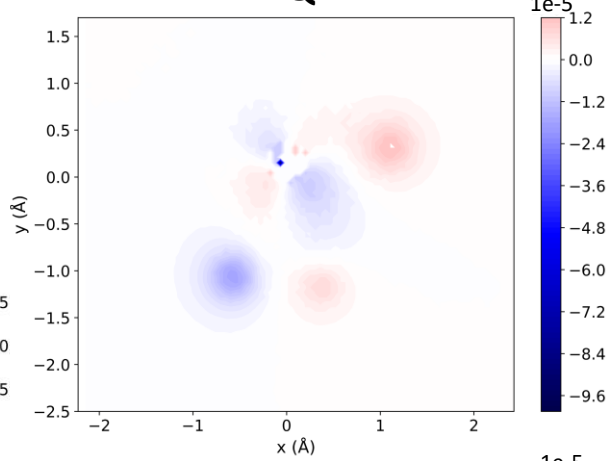**R = 1.4 Å**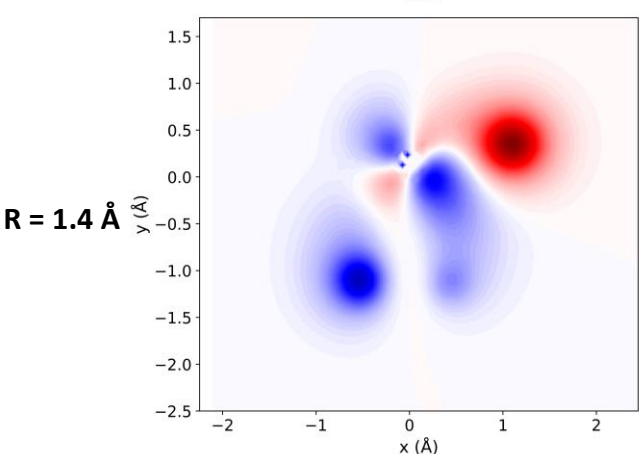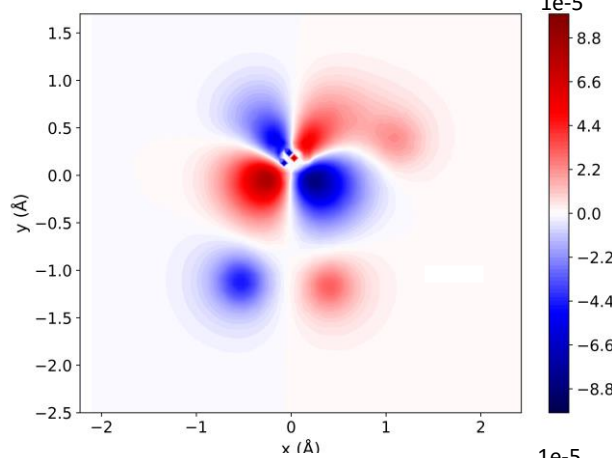**R = 1.6 Å**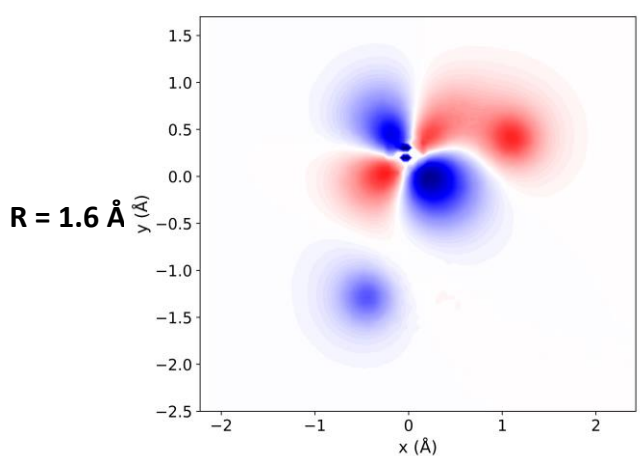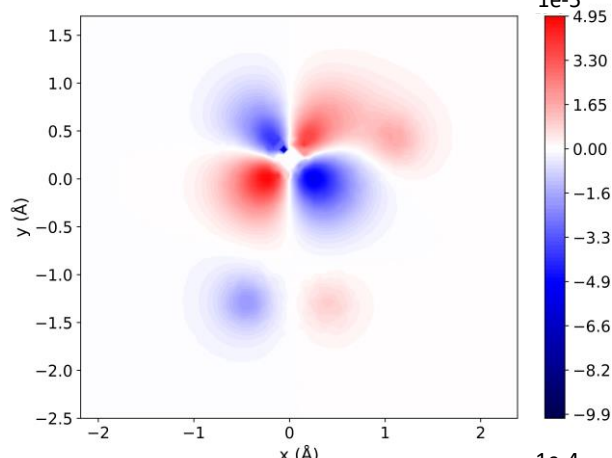**R = 1.8 Å**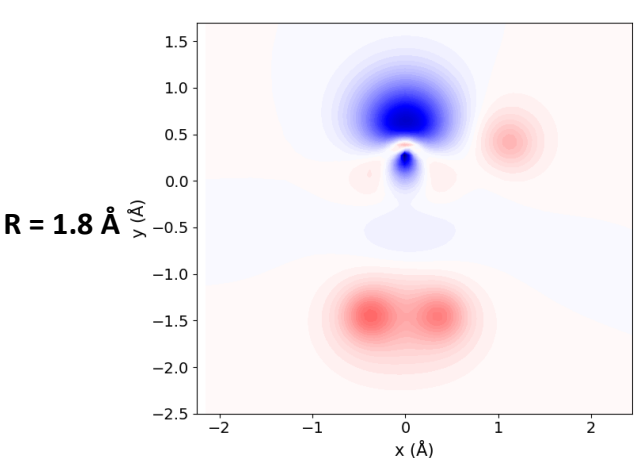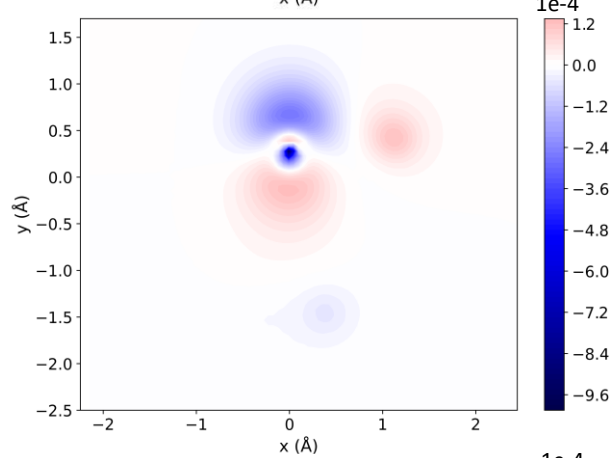**R = 2.1 Å**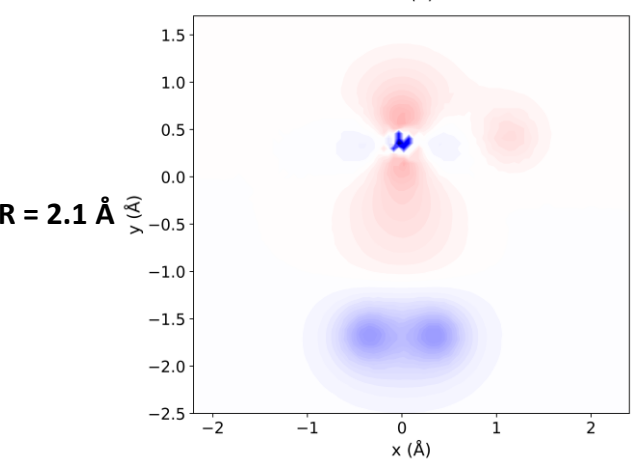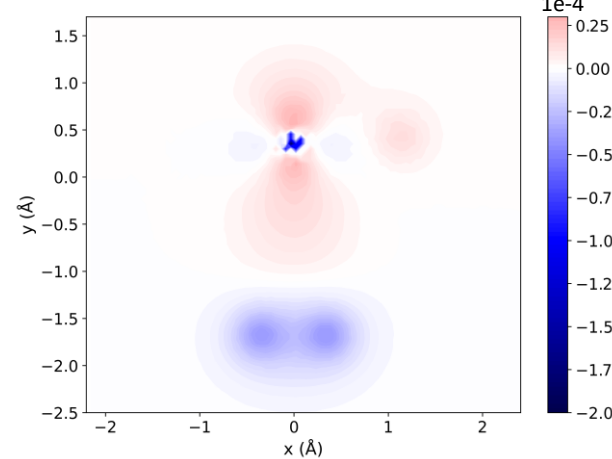

Supplement: Supplementary file 2 — Data S2: jcc70289‐sup‐0002‐Supinfo.zip. [file JCC-47-0-s002.zip › fig/results/dif-densidade-elec.pdf]

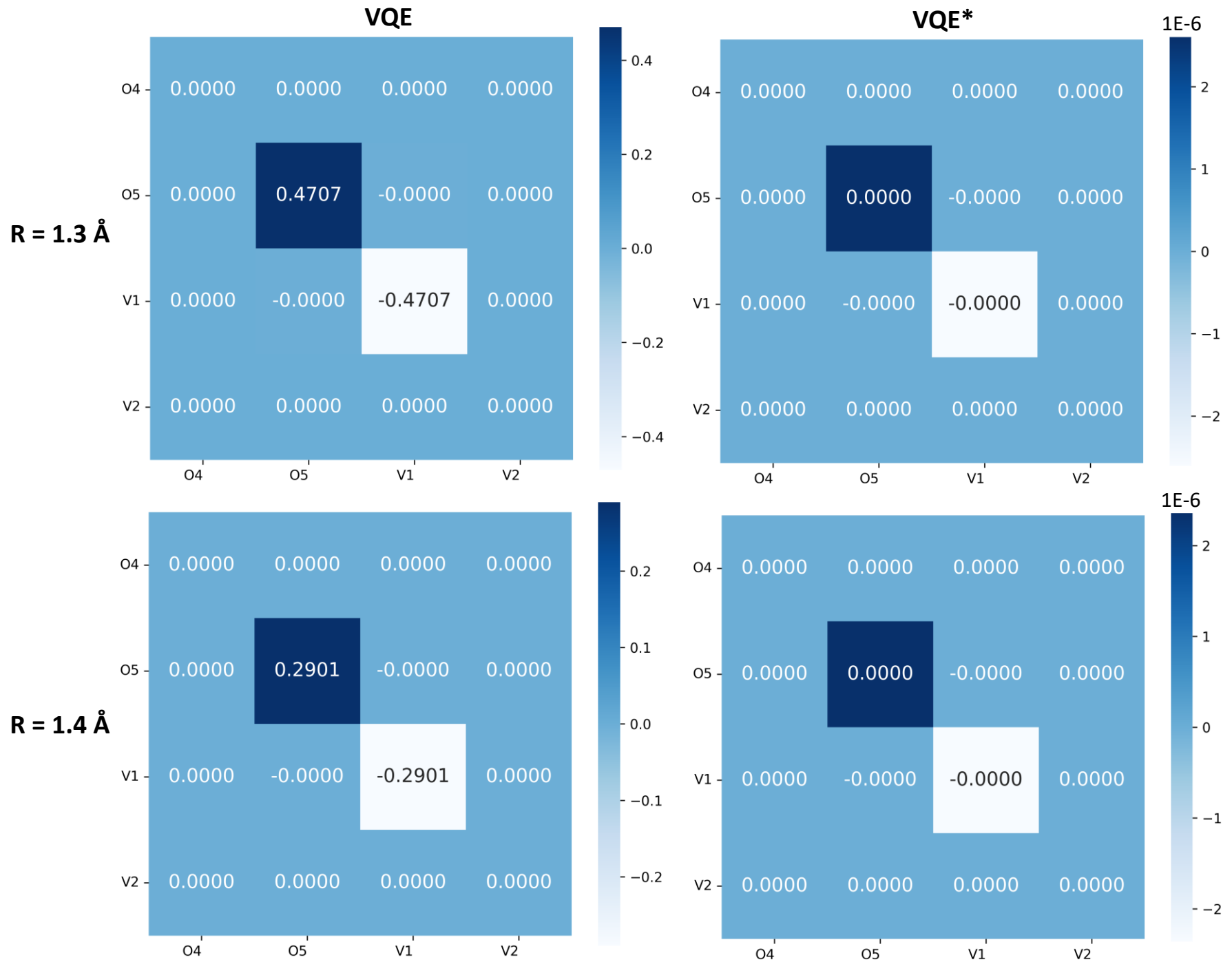

Supplement: Supplementary file 2 — Data S2: jcc70289‐sup‐0002‐Supinfo.zip. [file JCC-47-0-s002.zip › fig/results/gatefabric-as2-rdm.pdf]

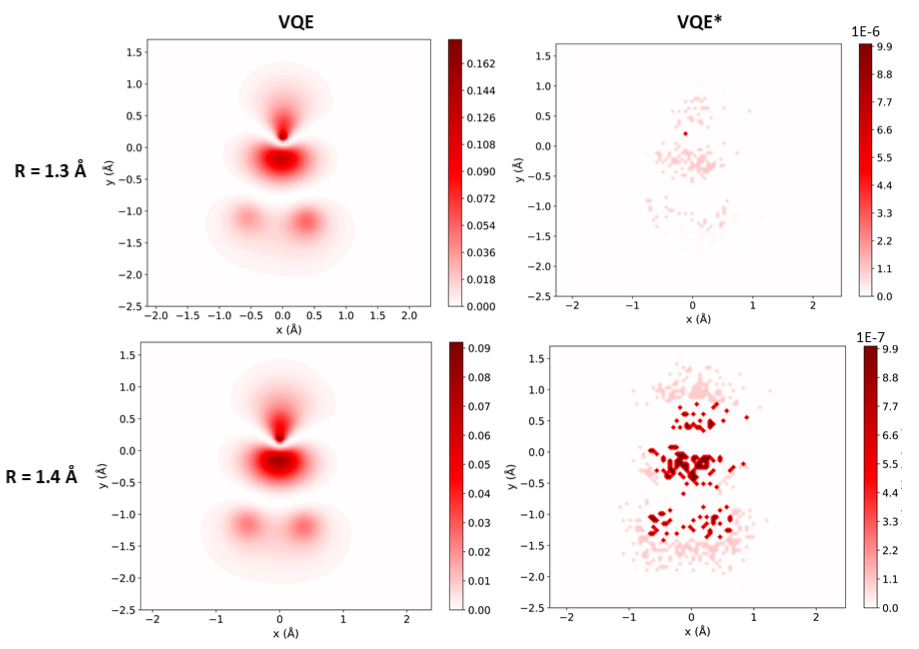

Supplement: Supplementary file 2 — Data S2: jcc70289‐sup‐0002‐Supinfo.zip. [file JCC-47-0-s002.zip › fig/results/gatefabric-as2-dens.png]

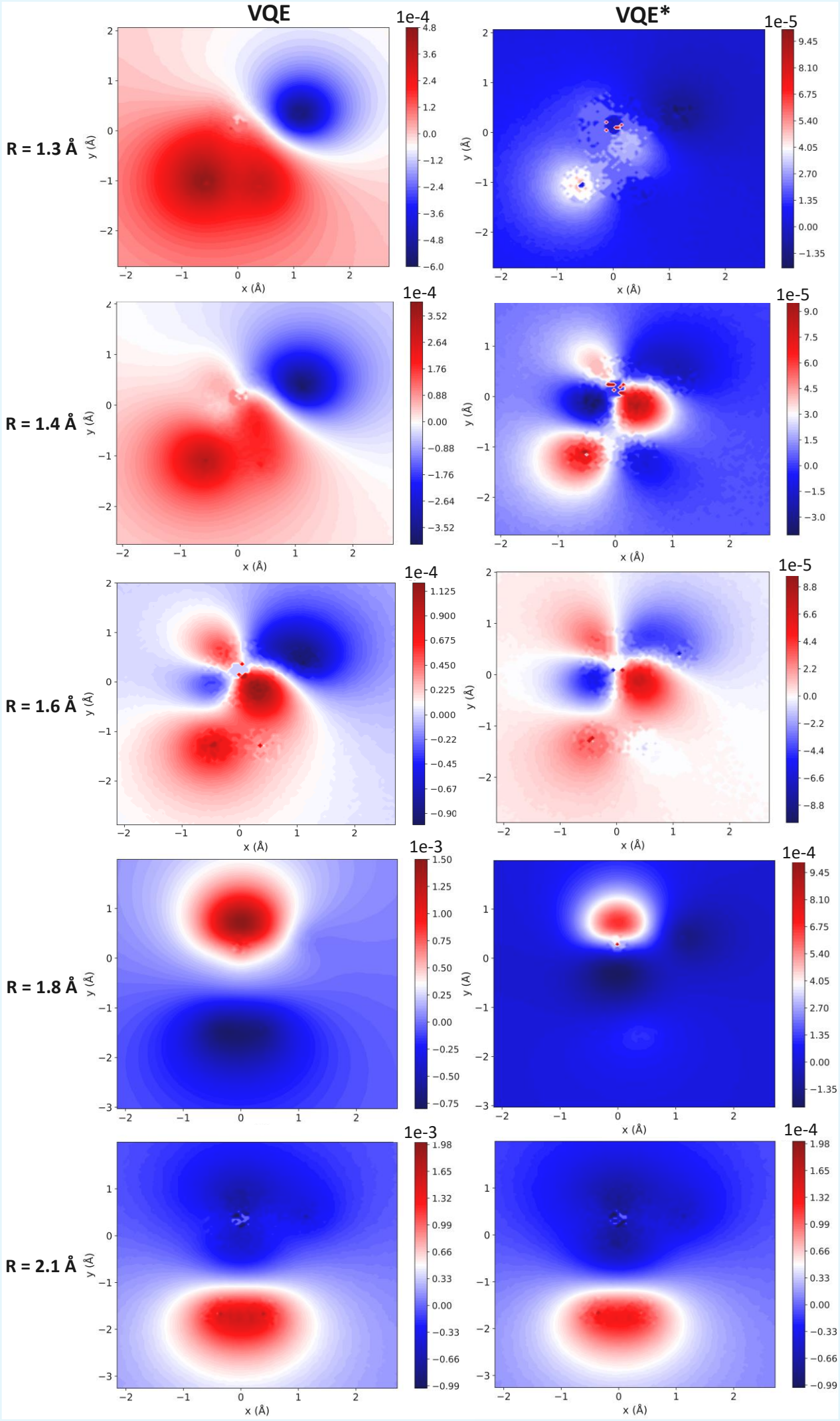

Supplement: Supplementary file 2 — Data S2: jcc70289‐sup‐0002‐Supinfo.zip. [file JCC-47-0-s002.zip › fig/results/dif-pot.pdf]

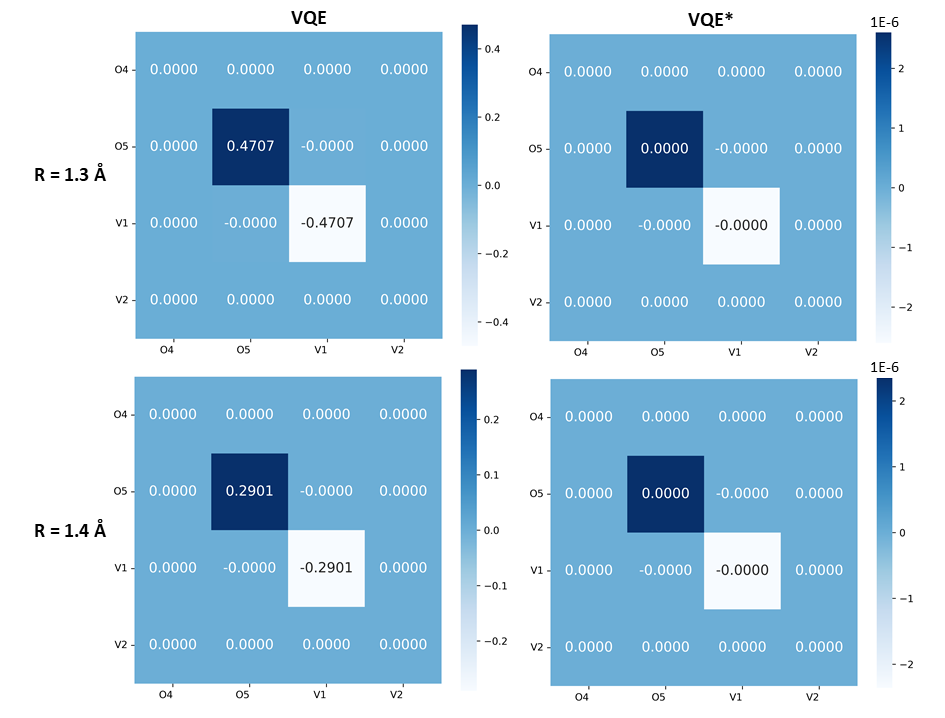

Supplement: Supplementary file 2 — Data S2: jcc70289‐sup‐0002‐Supinfo.zip. [file JCC-47-0-s002.zip › fig/results/gatefabric-as2-rdm.png]

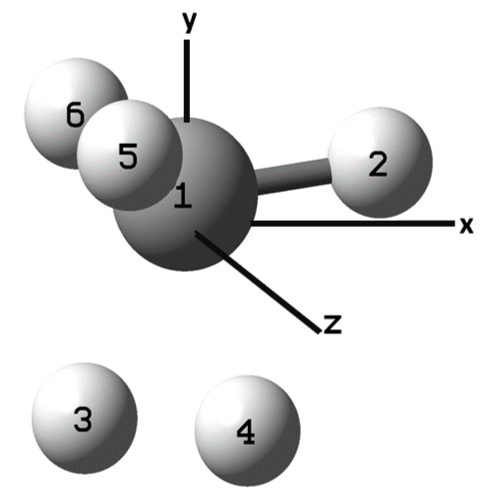

Supplement: Supplementary file 2 — Data S2: jcc70289‐sup‐0002‐Supinfo.zip. [file JCC-47-0-s002.zip › fig/results/mol.png]

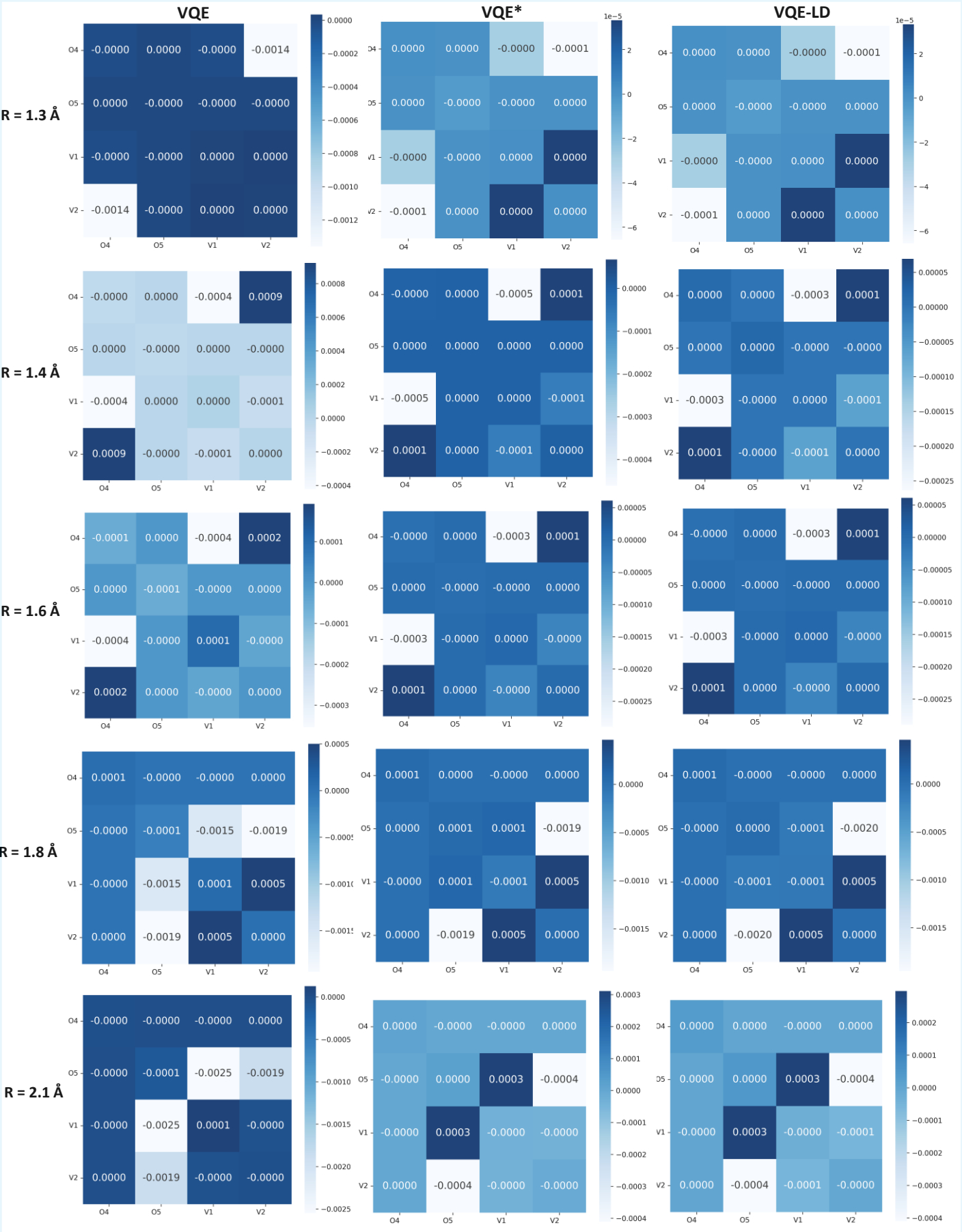

Supplement: Supplementary file 2 — Data S2: jcc70289‐sup‐0002‐Supinfo.zip. [file JCC-47-0-s002.zip › fig/results/rdm1-dif.pdf]

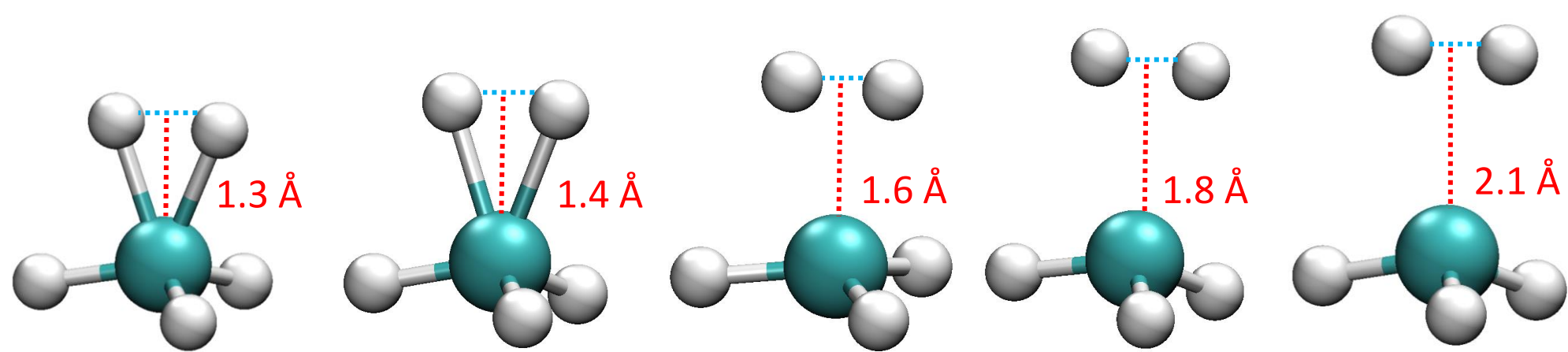

Supplement: Supplementary file 2 — Data S2: jcc70289‐sup‐0002‐Supinfo.zip. [file JCC-47-0-s002.zip › fig/results/ch5+-structure.pdf]

**R = 1.3 Å**

**VQE**

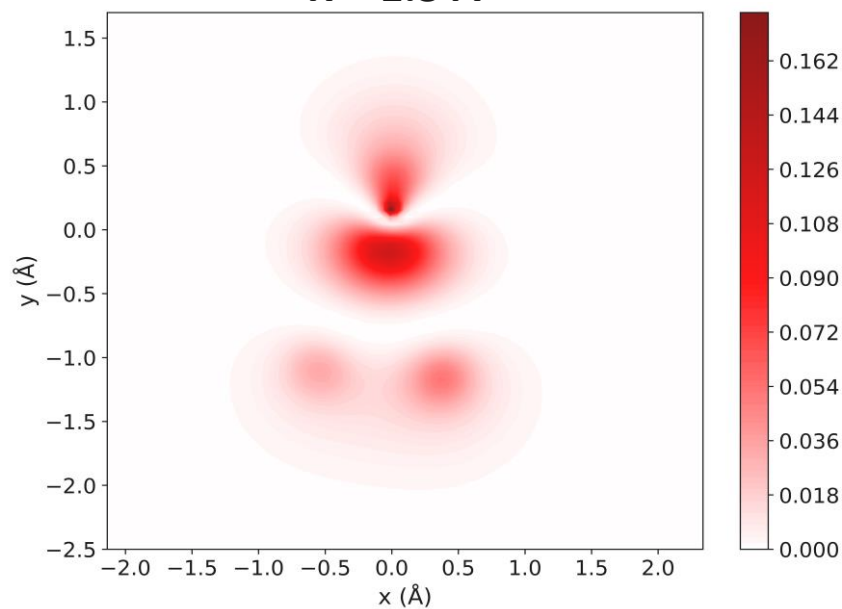

**R = 1.4 Å**

**VQE**

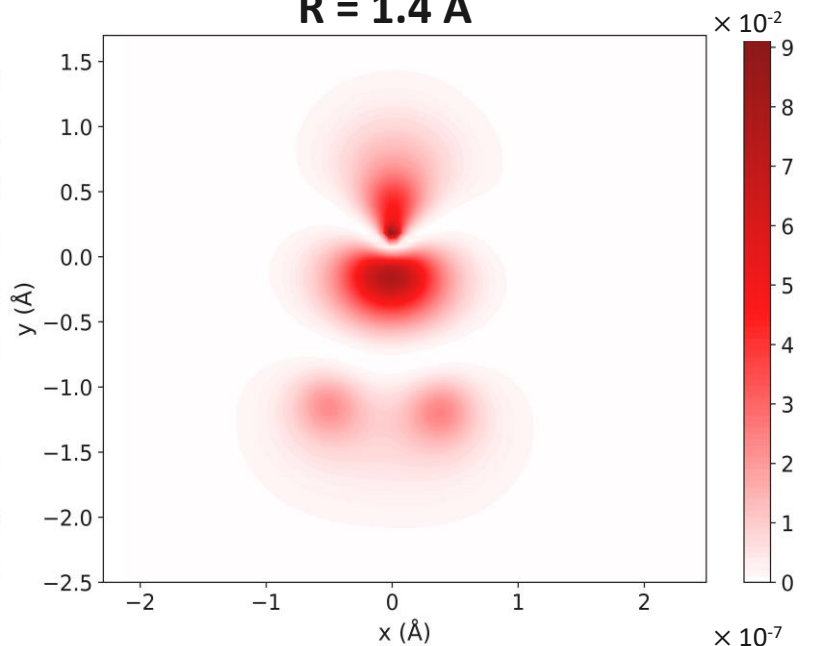

**VQE\***

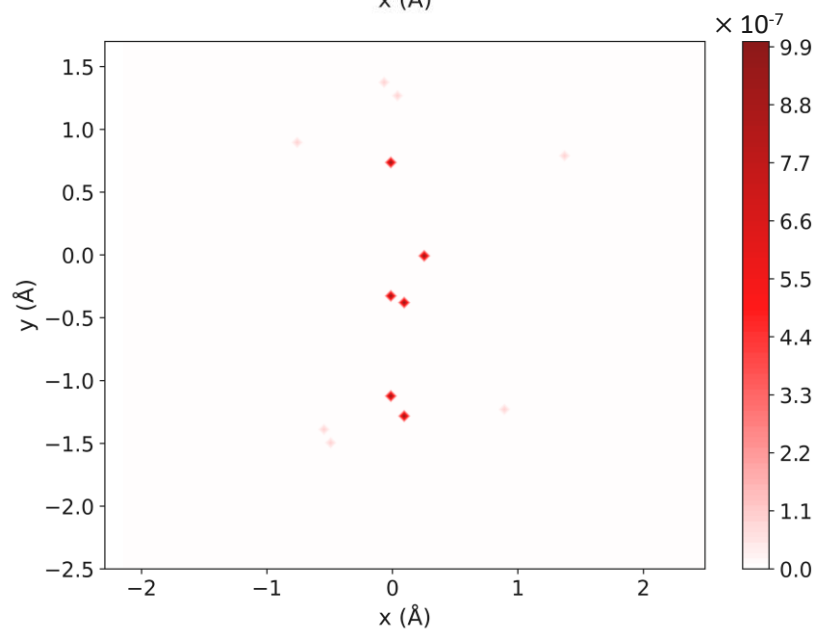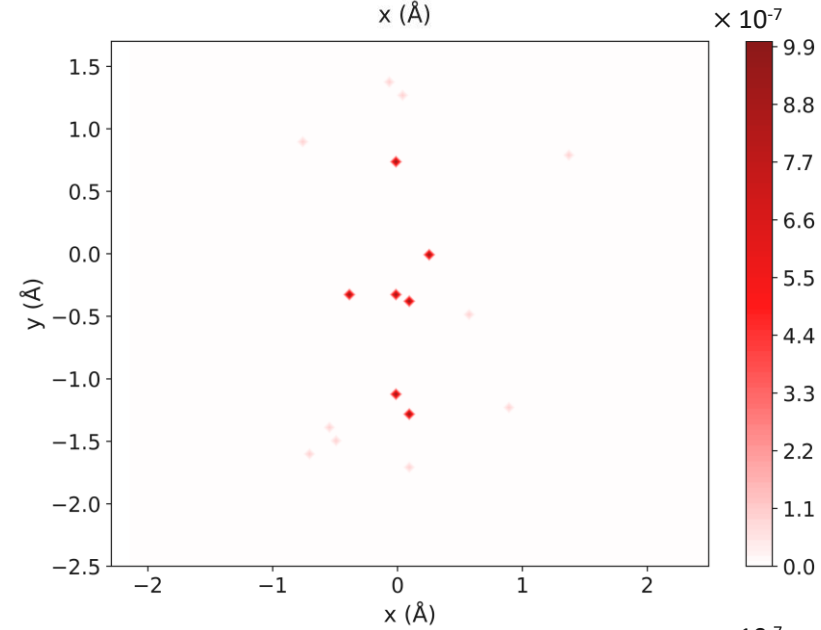

**VQE-LD**

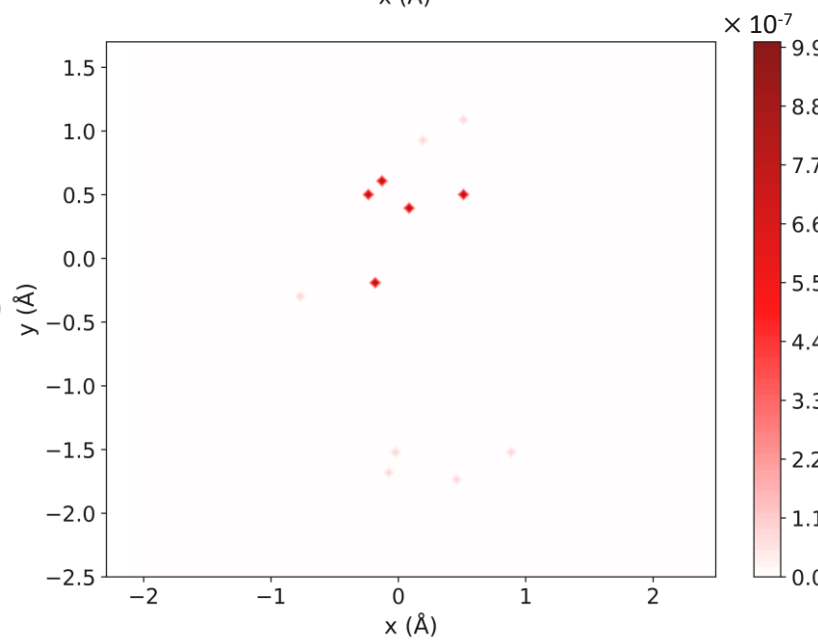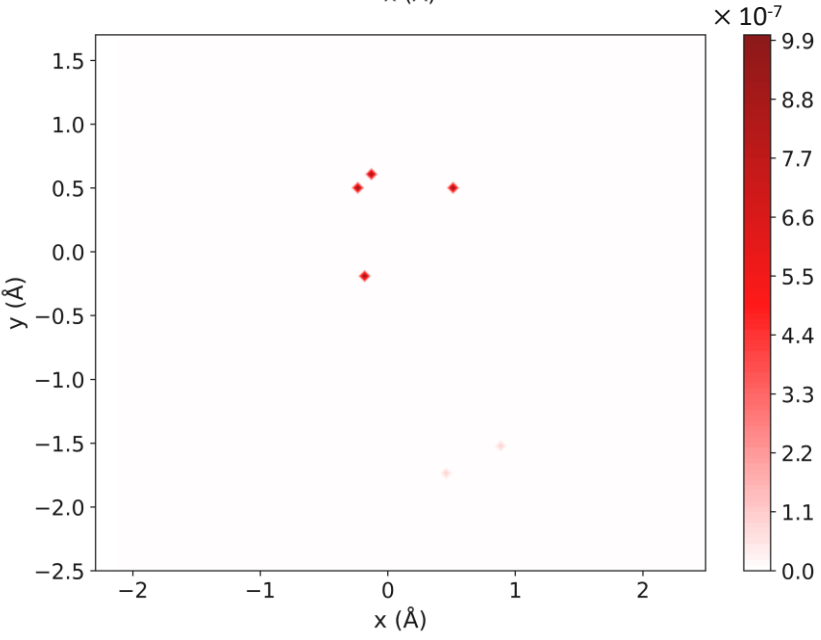

Supplement: Supplementary file 2 — Data S2: jcc70289‐sup‐0002‐Supinfo.zip. [file JCC-47-0-s002.zip › fig/results/gatefabric-as2-dens.pdf]

**R = 1.3 Å**

**VQE**

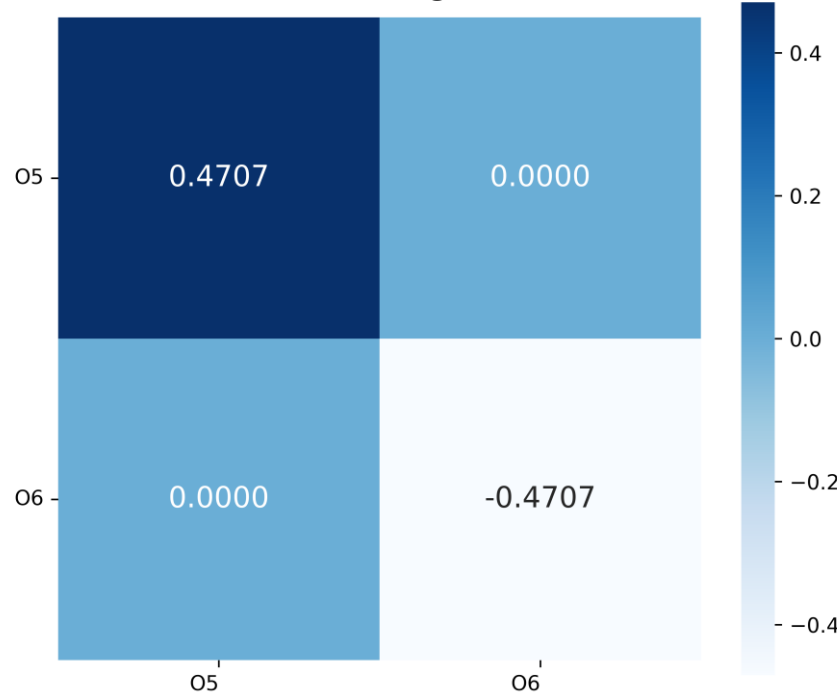

**R = 1.4 Å**

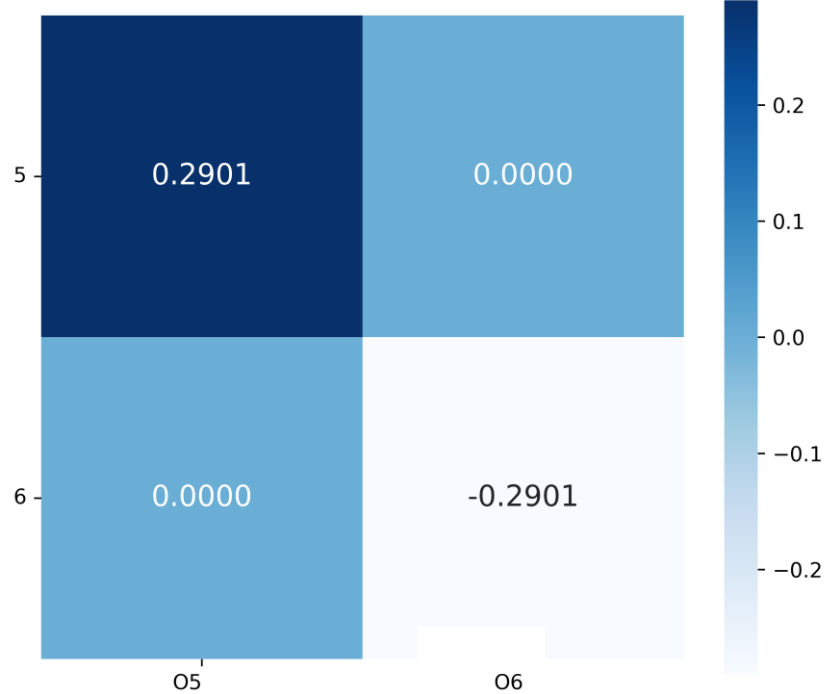

**VQE\***

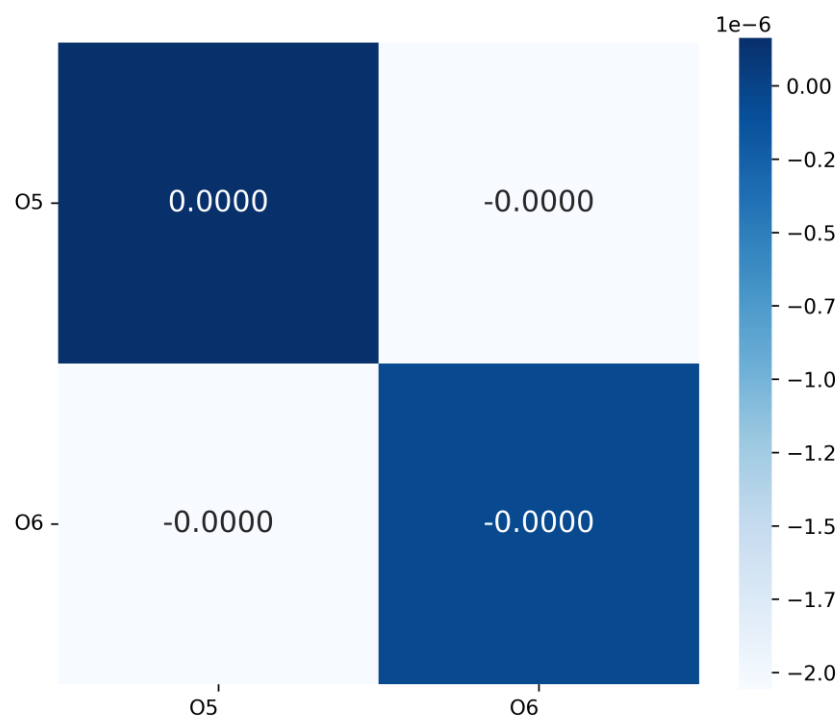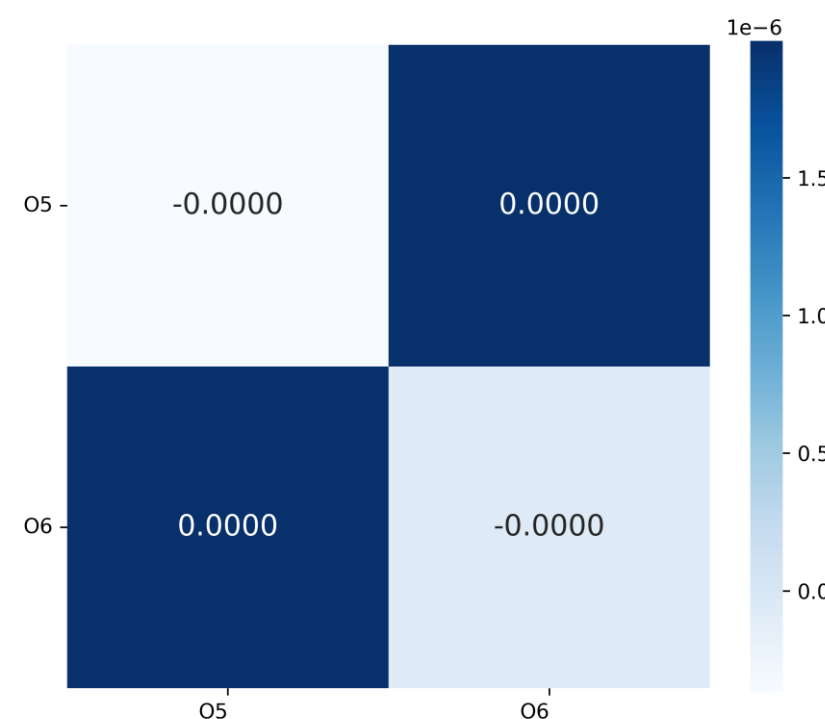

**VQE-LD**

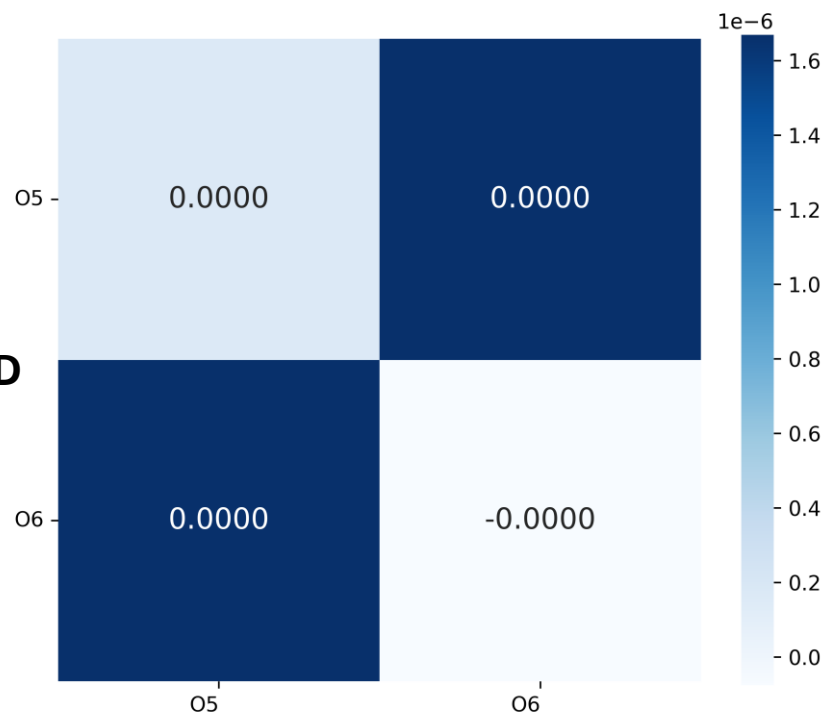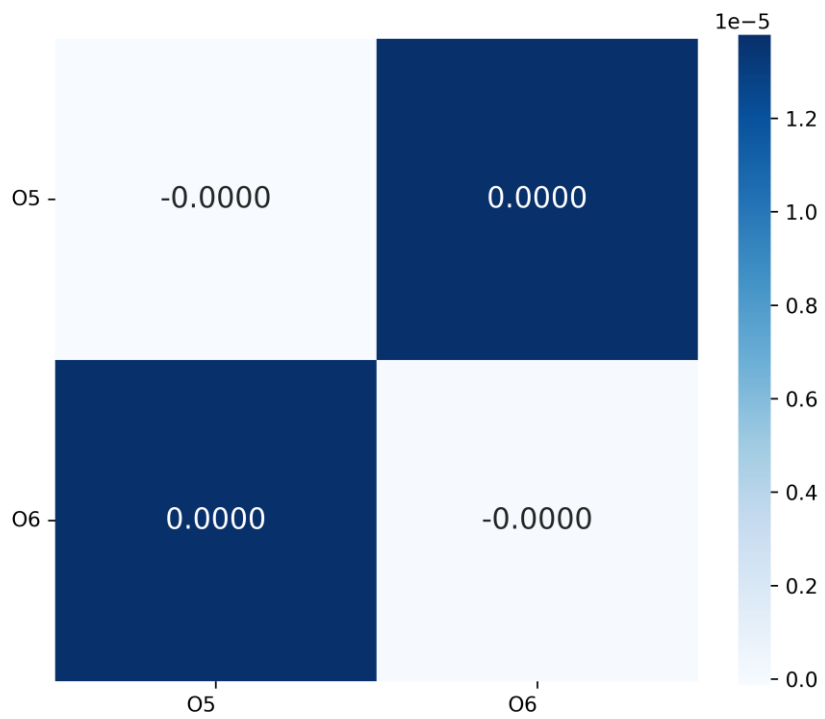

Supplement: Supplementary file 2 — Data S2: jcc70289‐sup‐0002‐Supinfo.zip. [file JCC-47-0-s002.zip › fig/results/gatefabric-as2.pdf]

**$R = 1.3 \text{ \AA}$**

**VQE**

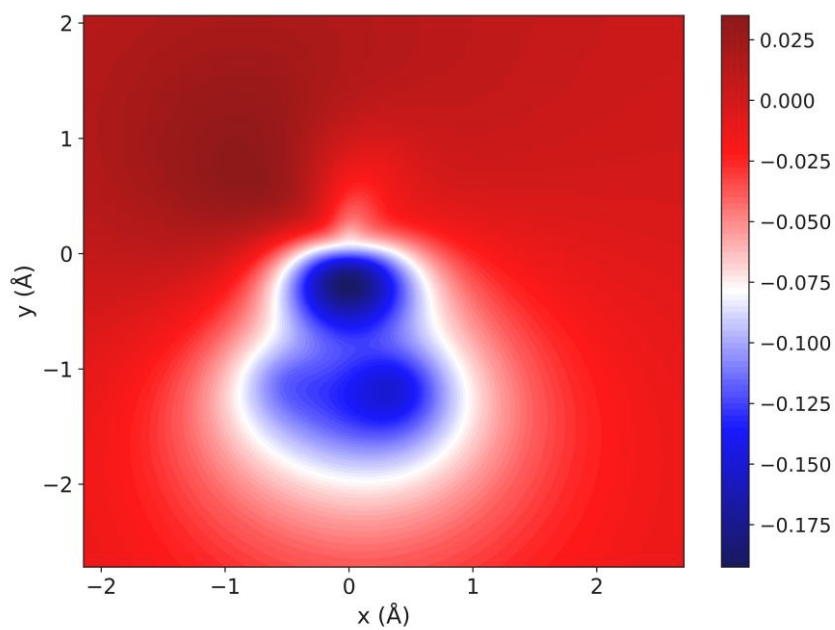

**$R = 1.4 \text{ \AA}$**

**VQE**

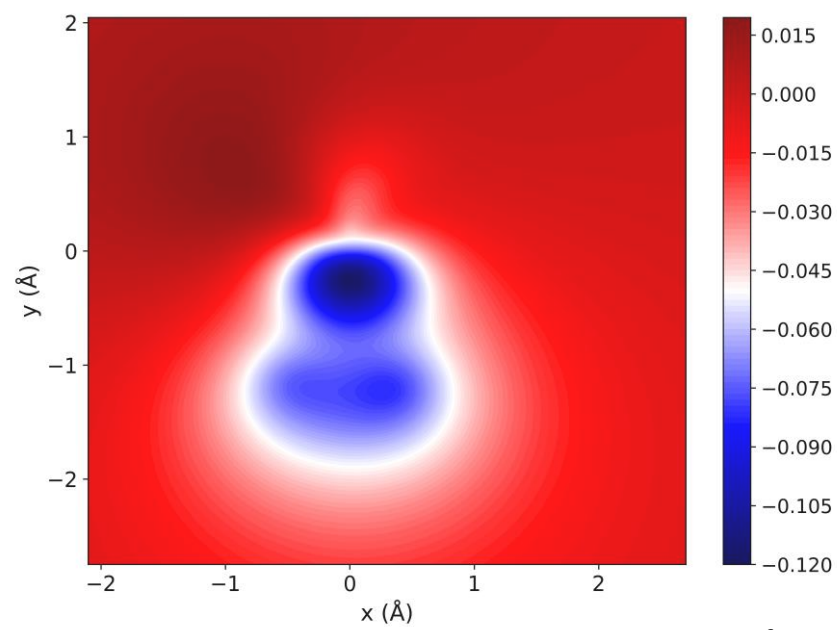

**VQE\***

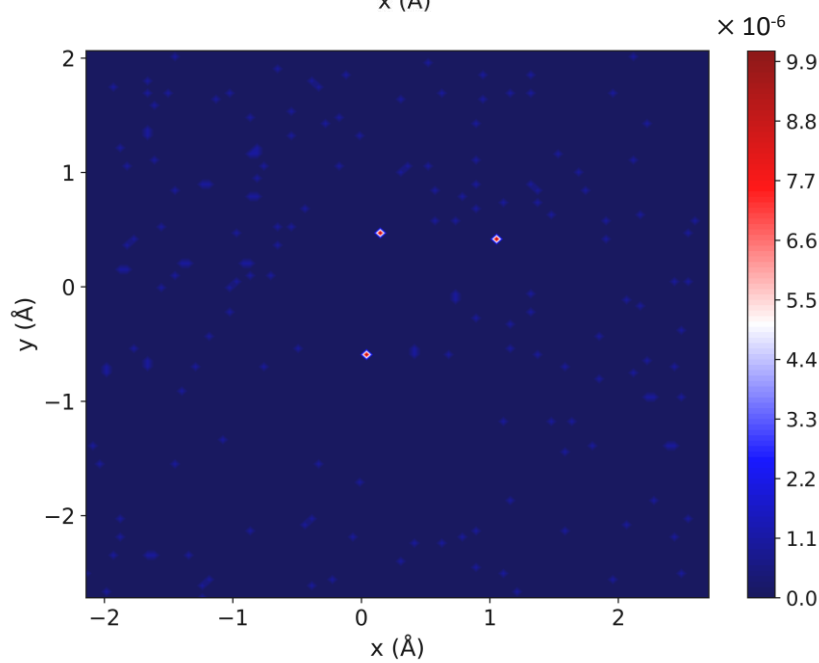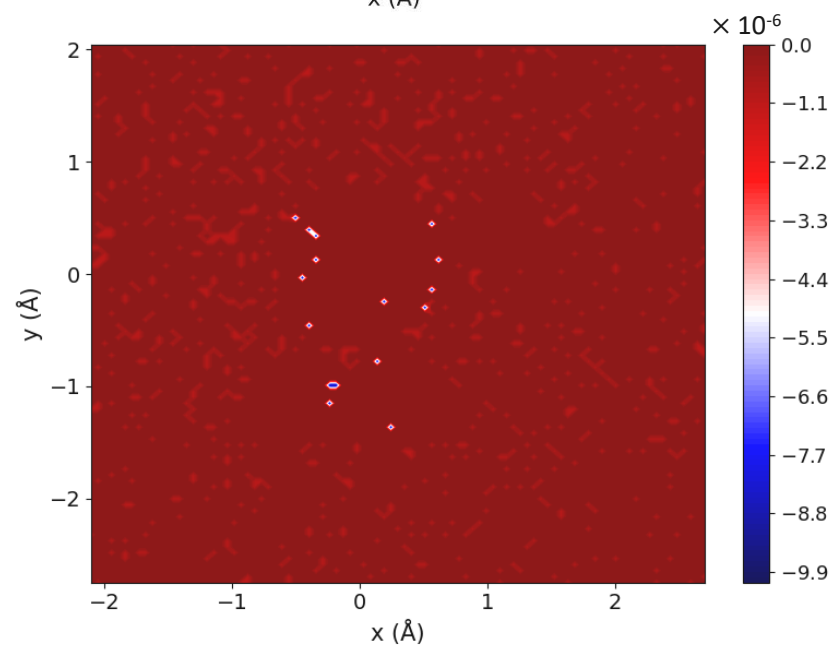

**VQE-LD**

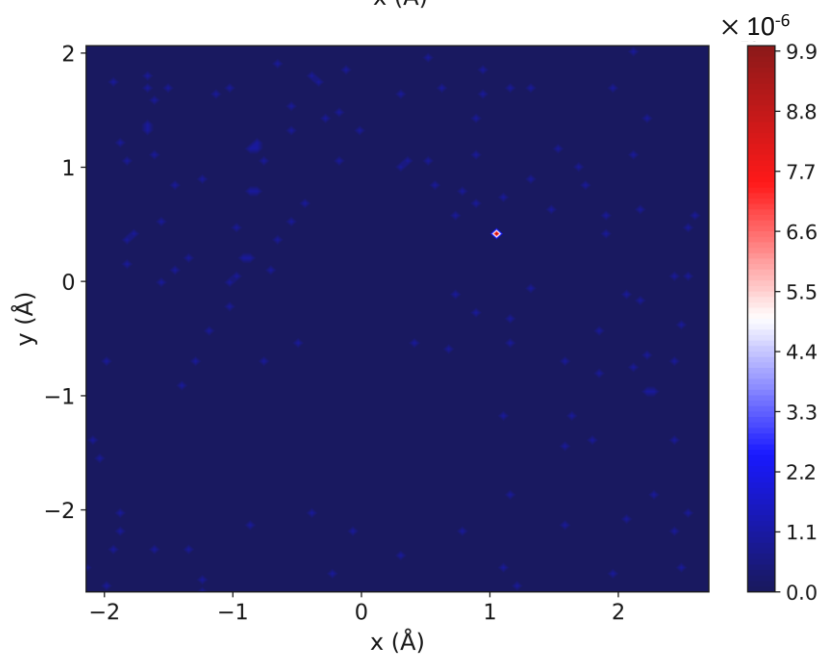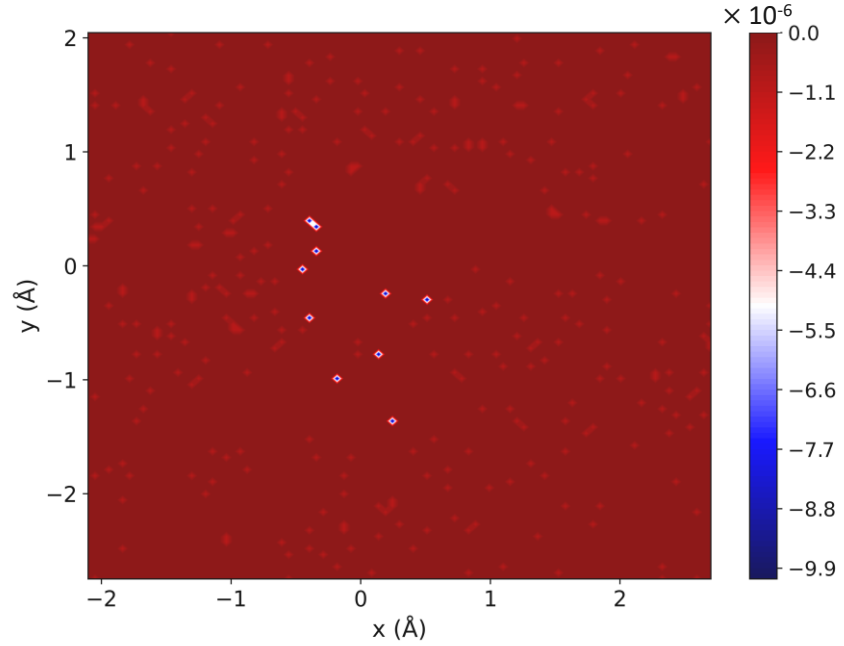

Supplement: Supplementary file 2 — Data S2: jcc70289‐sup‐0002‐Supinfo.zip. [file JCC-47-0-s002.zip › fig/results/gatefabric-as2-elec-pot.pdf]

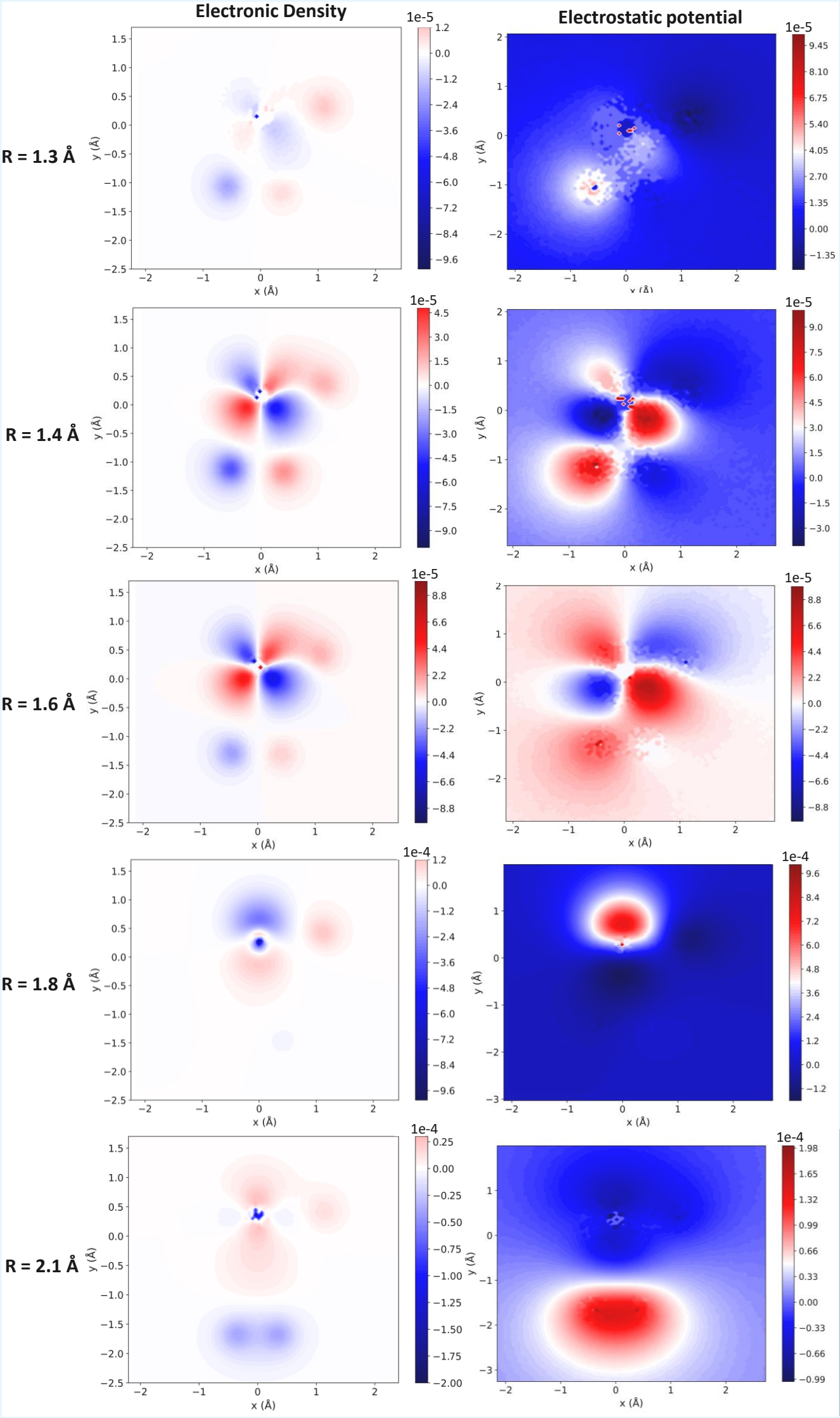

Supplement: Supplementary file 2 — Data S2: jcc70289‐sup‐0002‐Supinfo.zip. [file JCC-47-0-s002.zip › fig/results/dif-pot-vqe-ld.pdf]

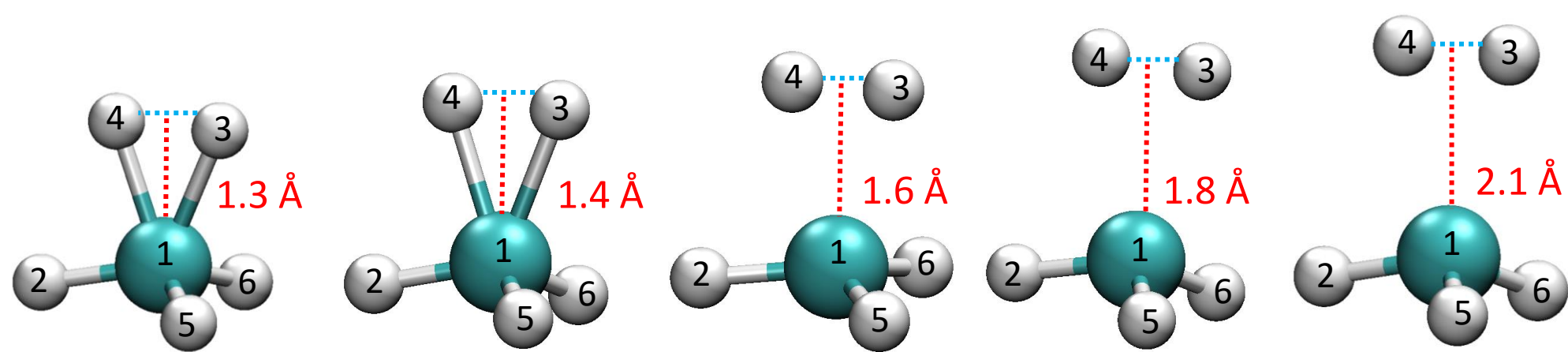

Supplement: Supplementary file 2 — Data S2: jcc70289‐sup‐0002‐Supinfo.zip. [file JCC-47-0-s002.zip › fig/results/structure.pdf]

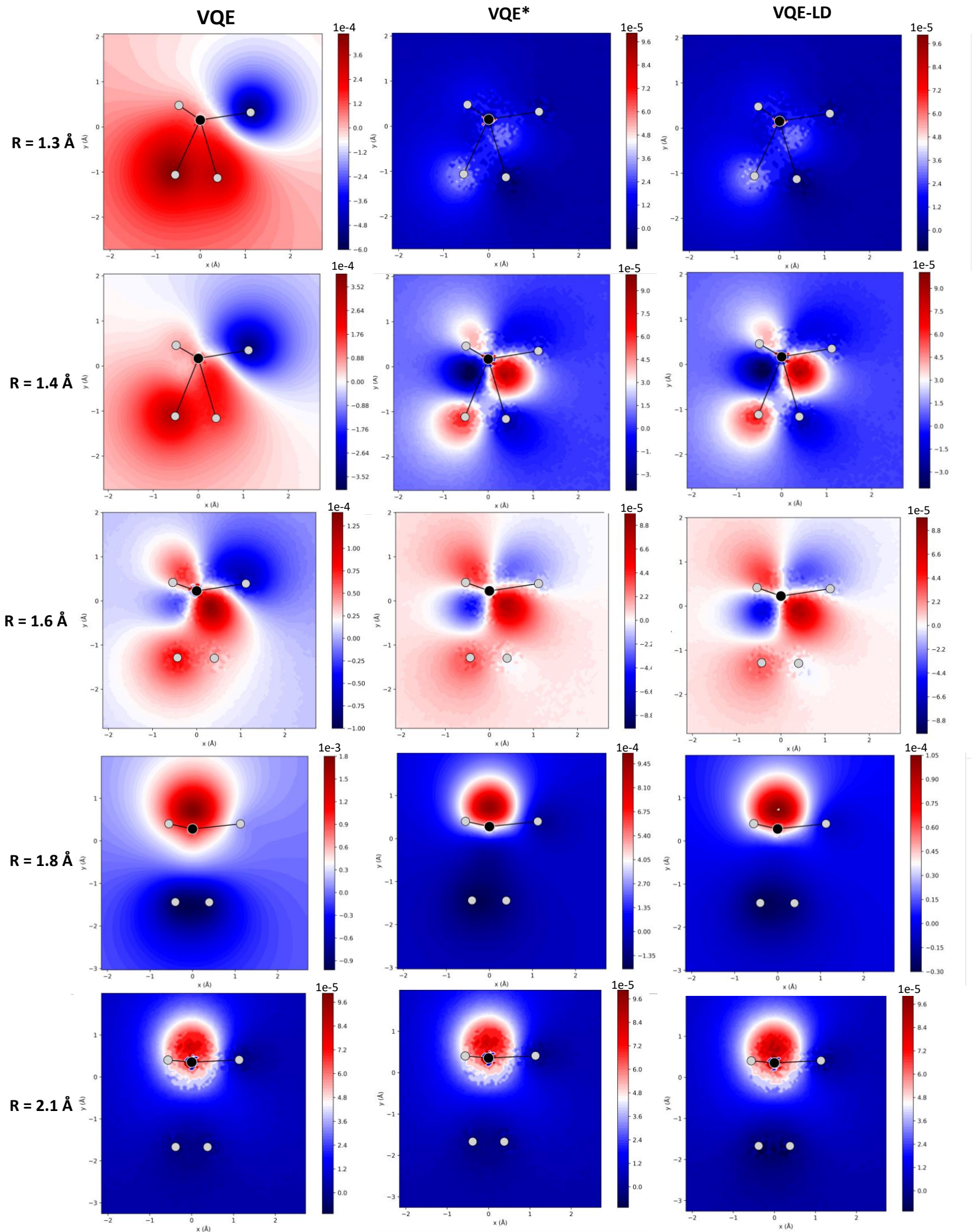

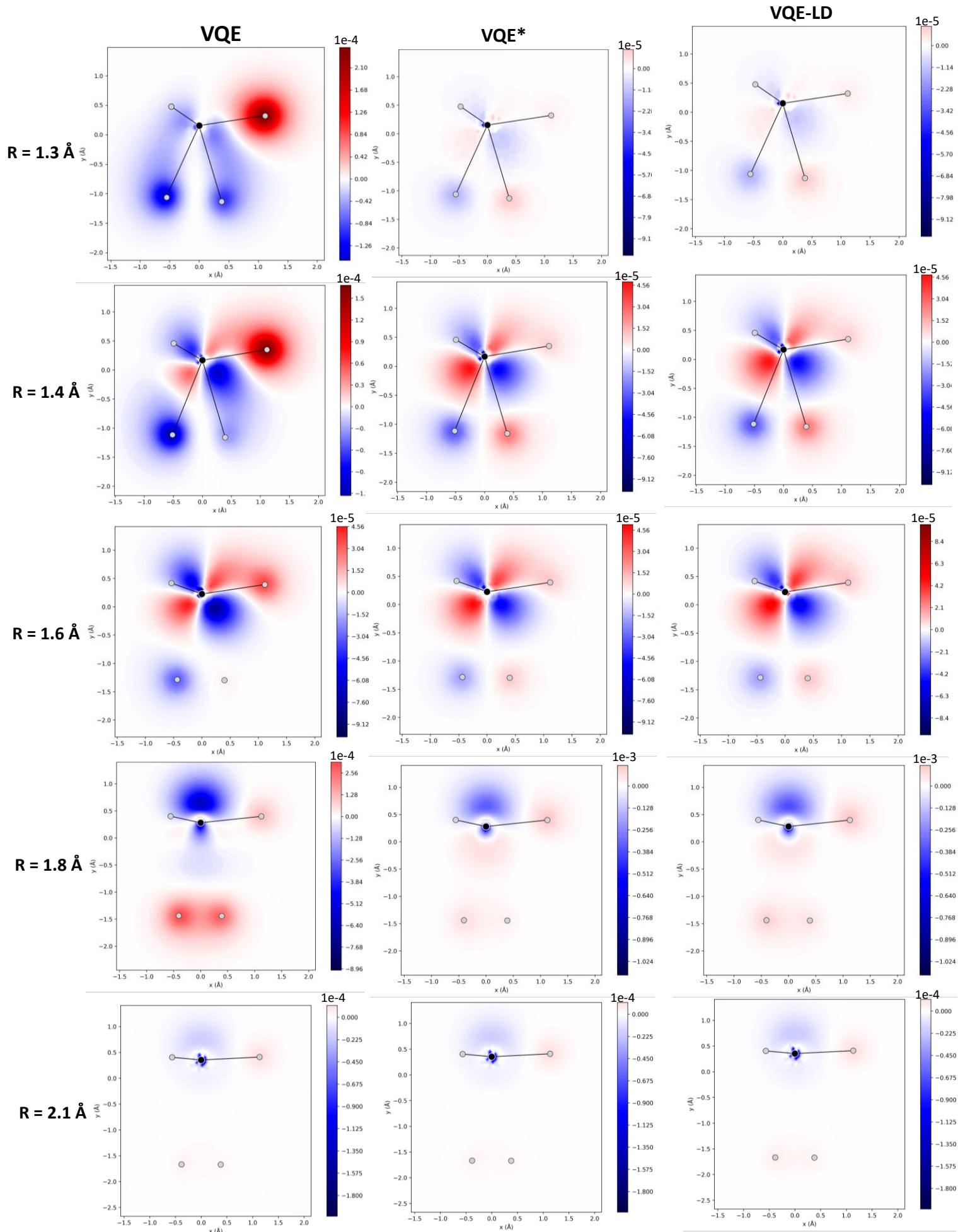

VQE

R = 1.3 Å

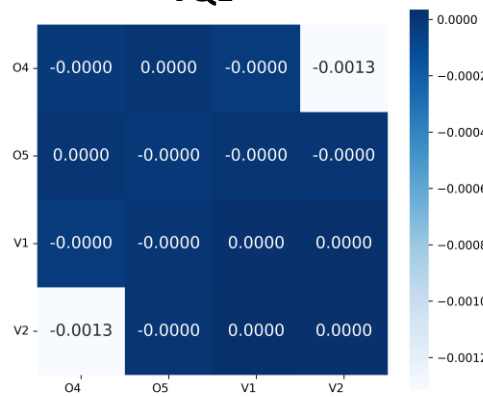

VQE\*

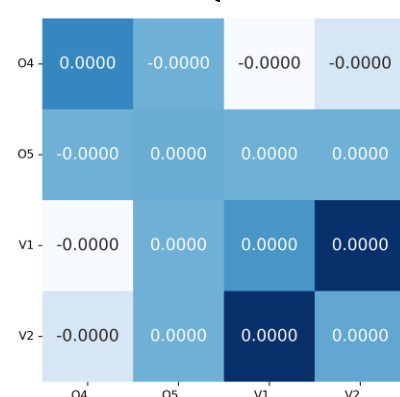

VQE-LD

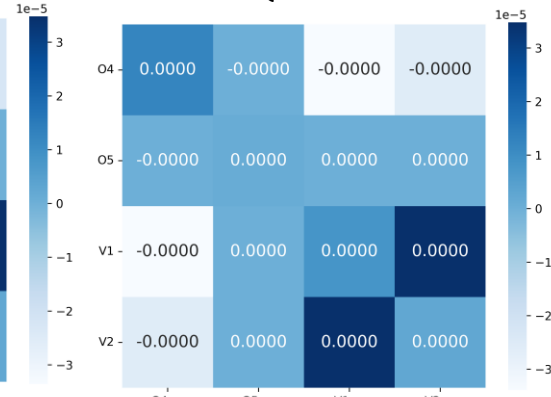

R = 1.4 Å

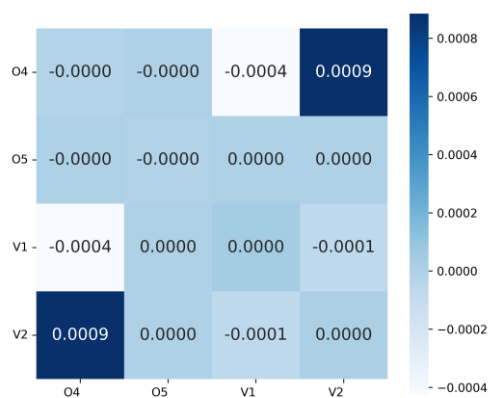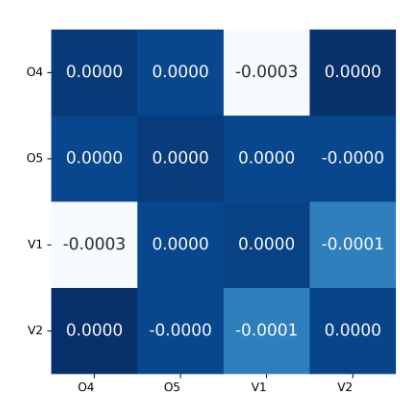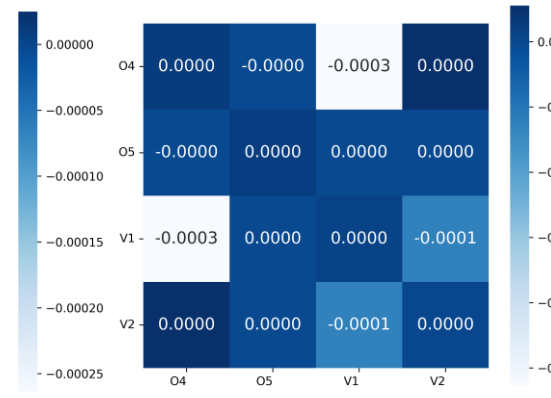

R = 1.6 Å

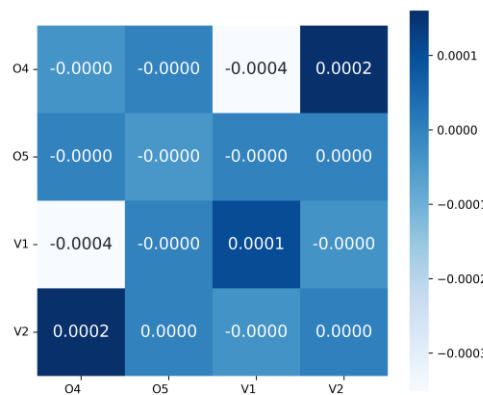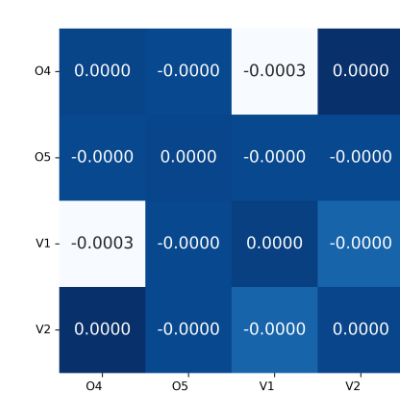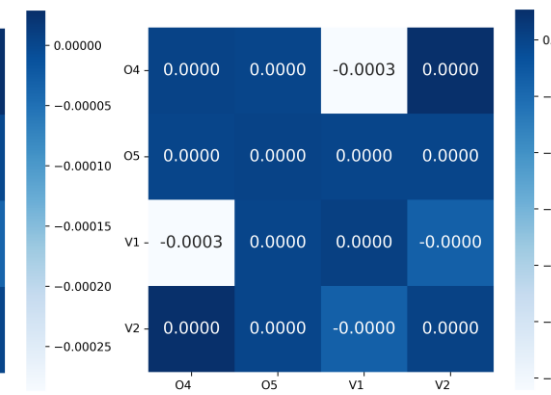

R = 1.8 Å

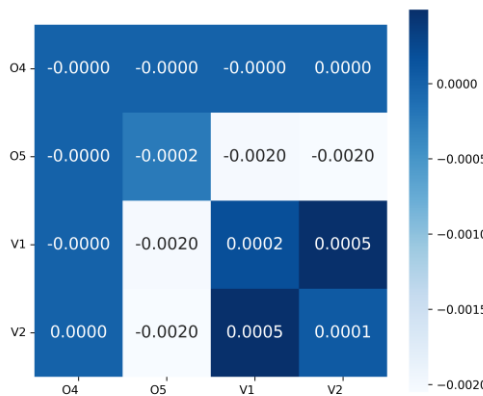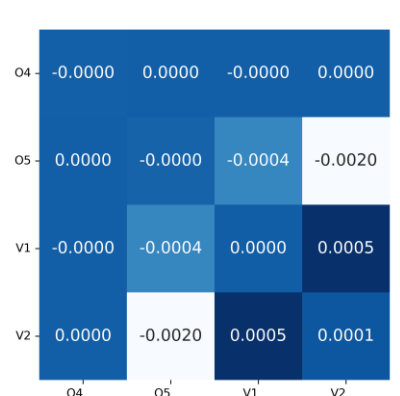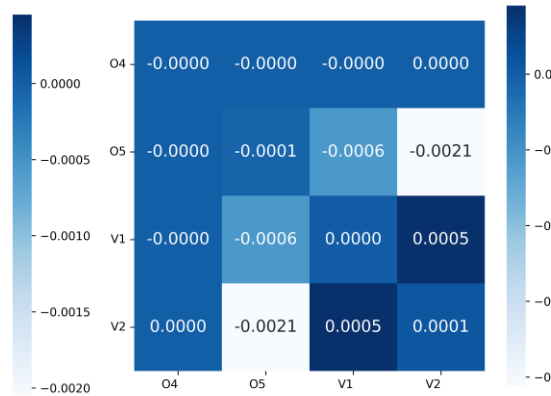

R = 2.1 Å

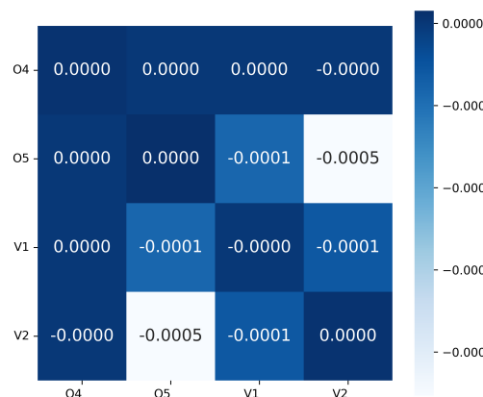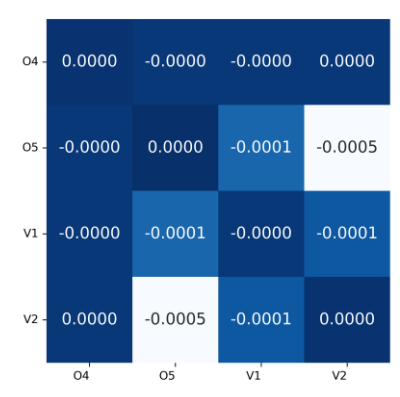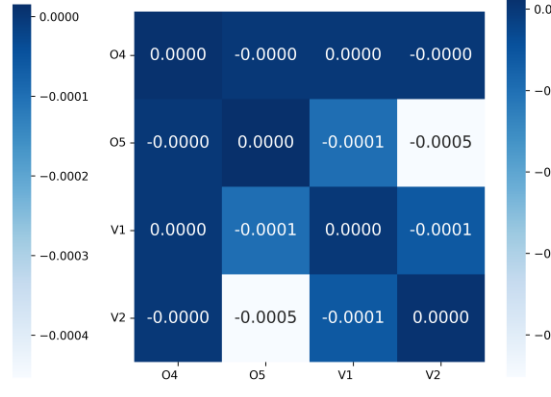

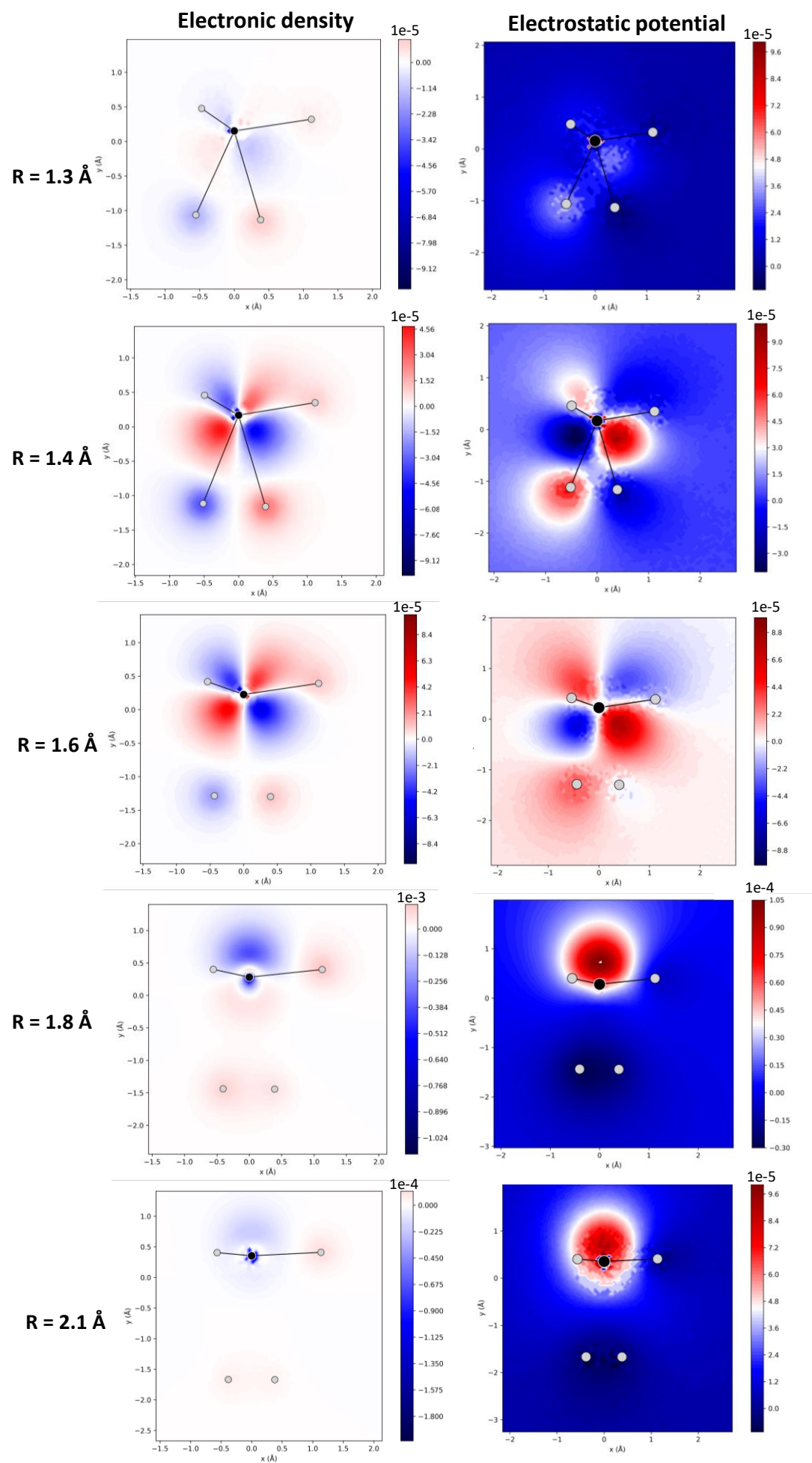

Supplement: Supplementary file 2 — Data S2: jcc70289‐sup‐0002‐Supinfo.zip. [file JCC-47-0-s002.zip › fig/fig-casci/dif -casci(4,4).pdf]

**VQE****R = 1.3 Å**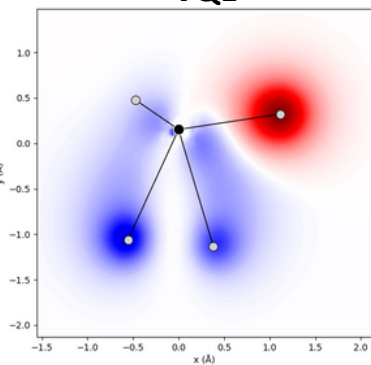**VQE\***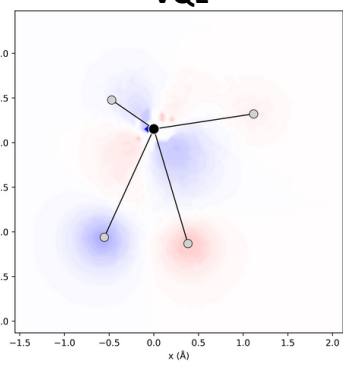**R = 1.4 Å**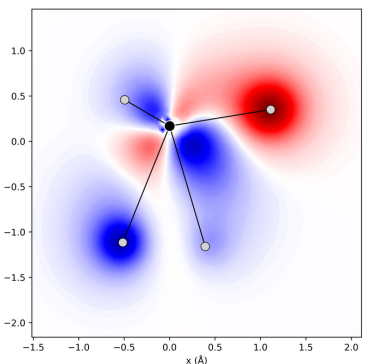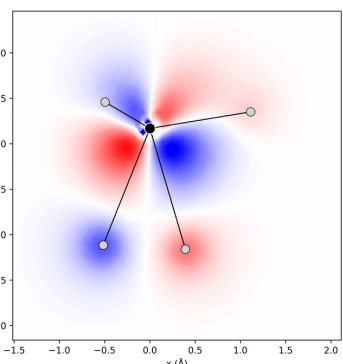**R = 1.6 Å**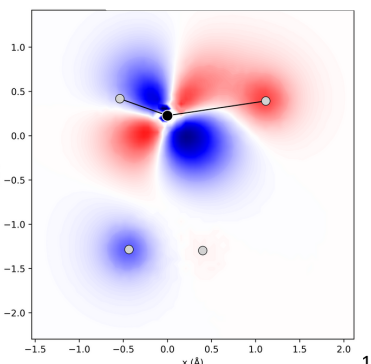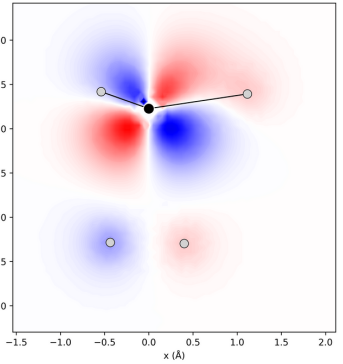**R = 1.8 Å**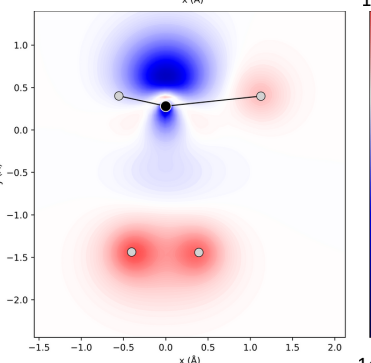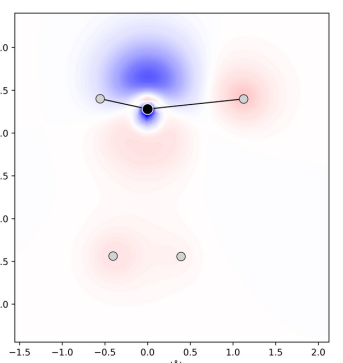**R = 2.1 Å**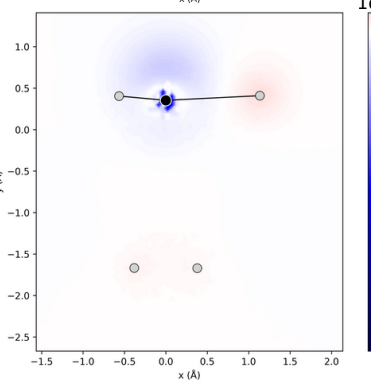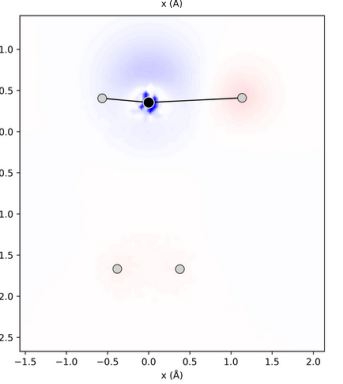

Supplement: Supplementary file 2 — Data S2: jcc70289‐sup‐0002‐Supinfo.zip. [file JCC-47-0-s002.zip › fig/fig-casci/dif-casci(4,4)-dens.pdf]

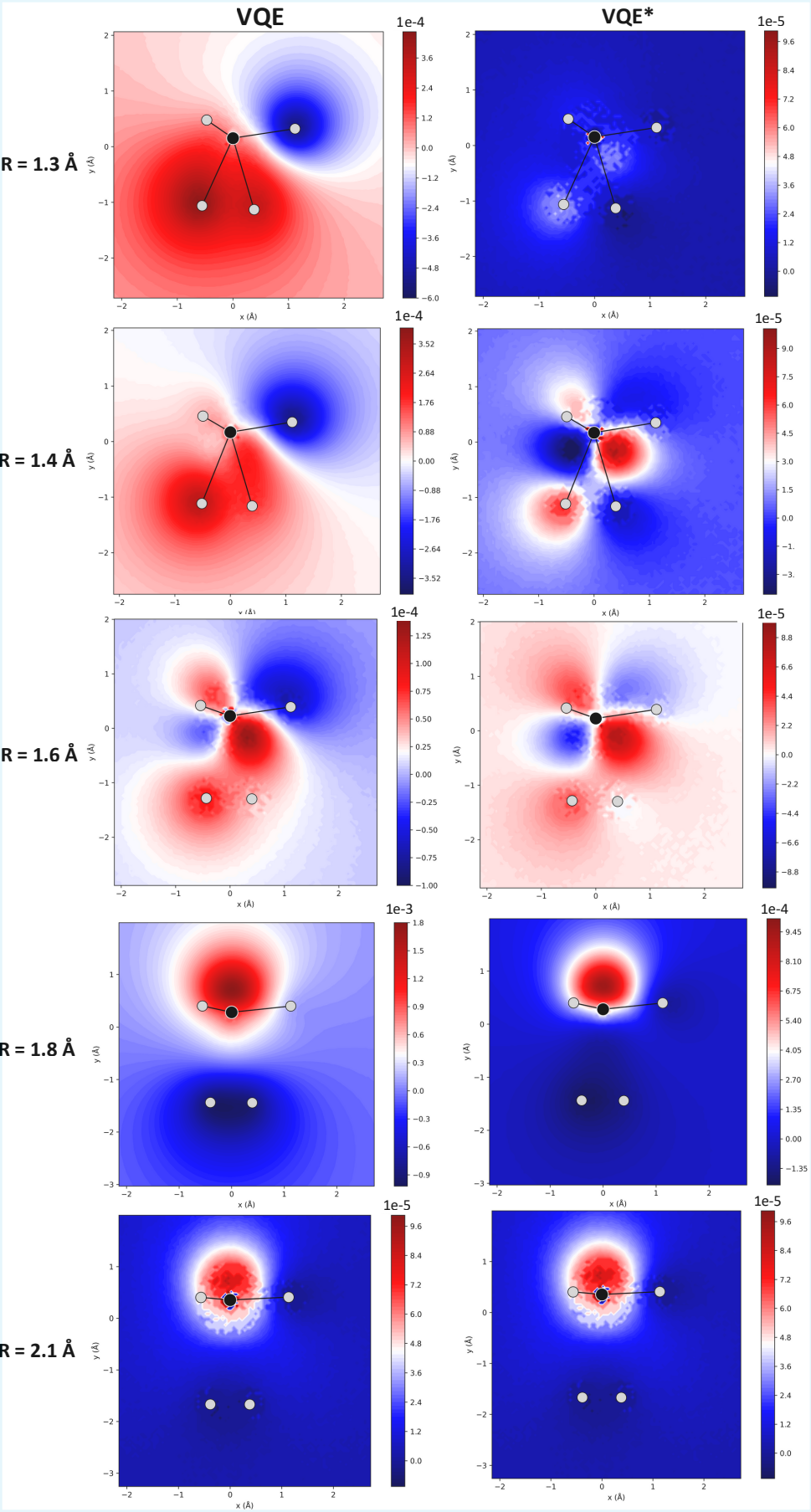

Supplement: Supplementary file 2 — Data S2: jcc70289‐sup‐0002‐Supinfo.zip. [file JCC-47-0-s002.zip › fig/fig-casci/dif-casci(4,4)-pot.pdf]

VQE

R = 1.3 Å

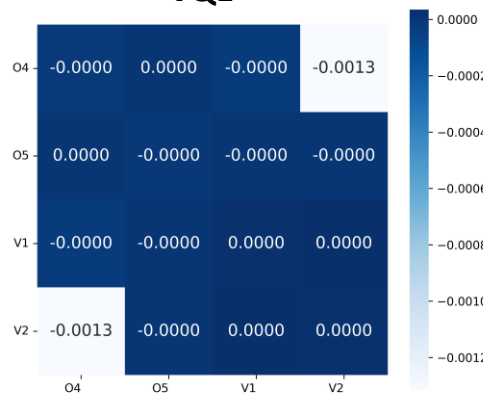

VQE\*

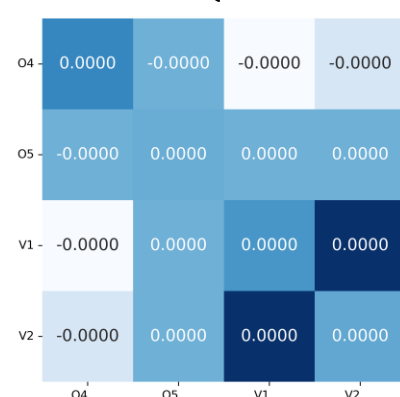

VQE-LD

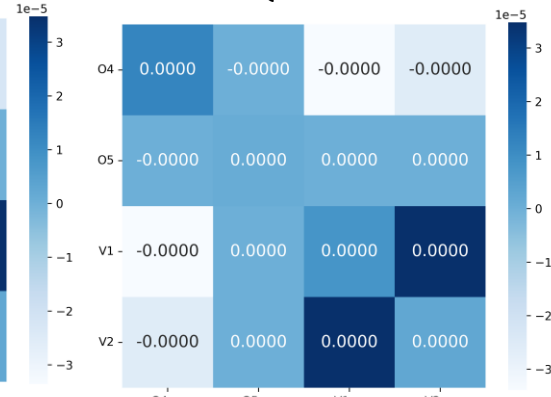

R = 1.4 Å

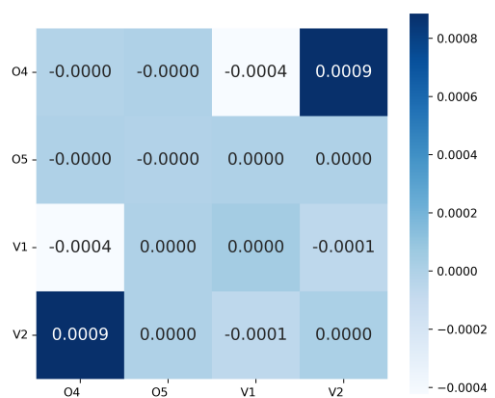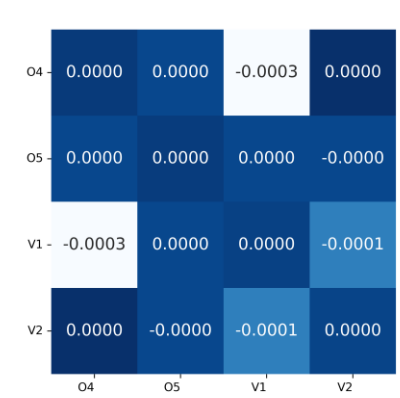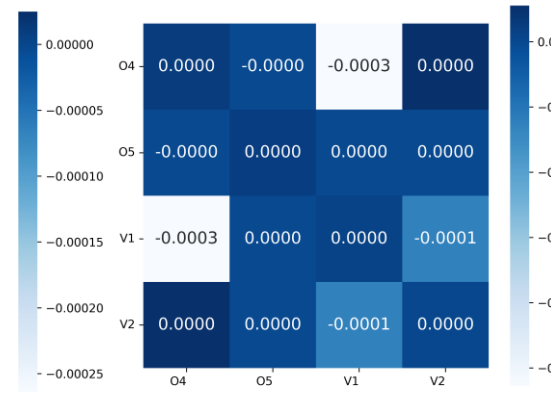

R = 1.6 Å

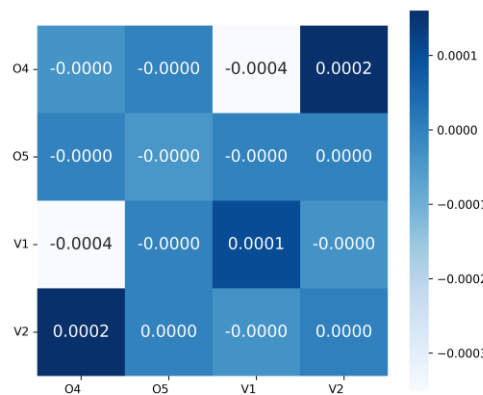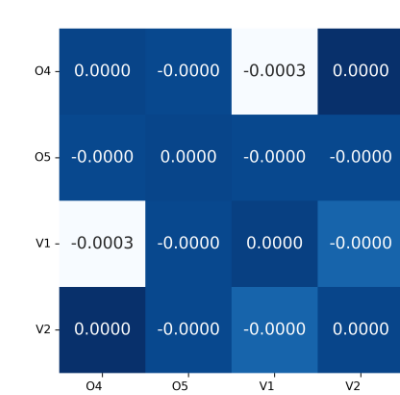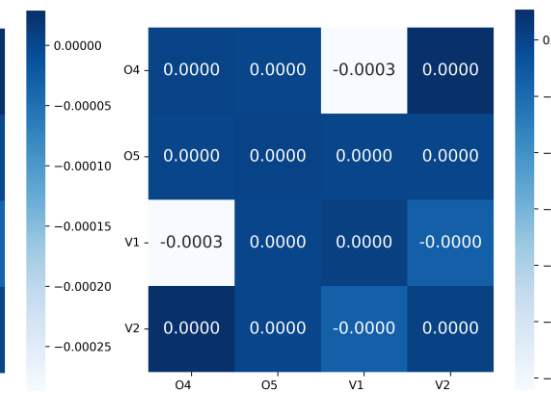

R = 1.8 Å

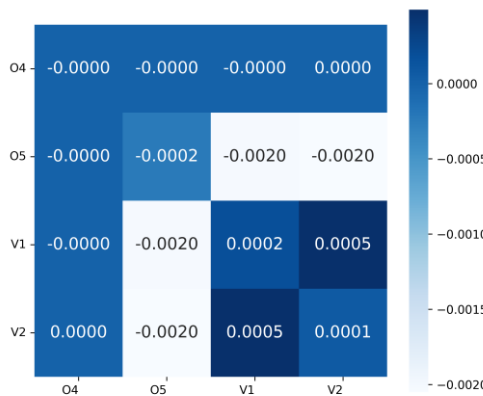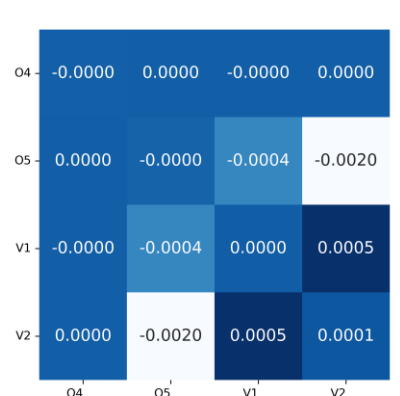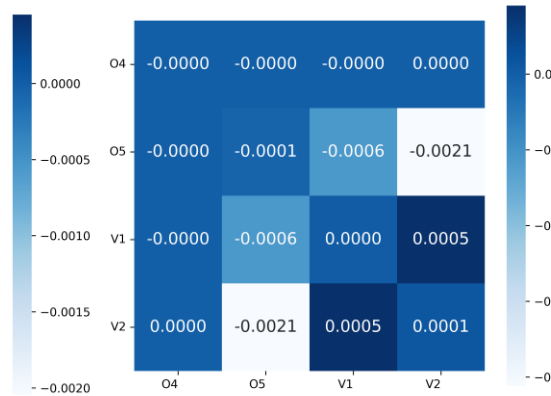

R = 2.1 Å

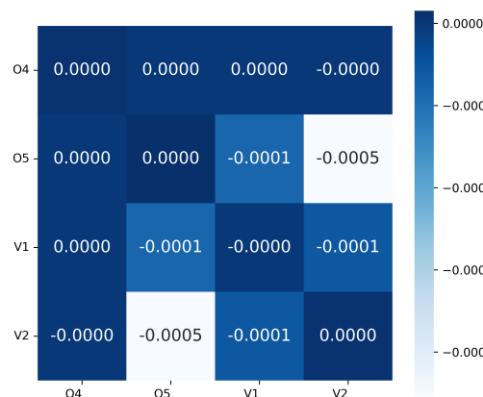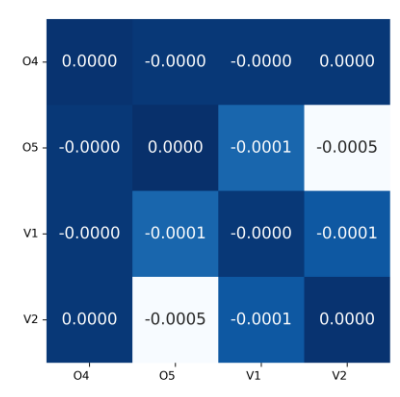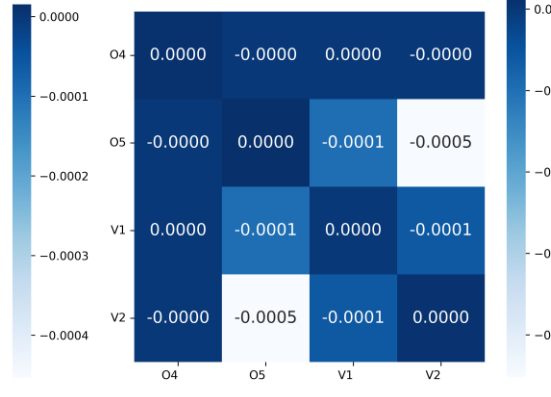

Supplement: Supplementary file 2 — Data S2: jcc70289‐sup‐0002‐Supinfo.zip. [file JCC-47-0-s002.zip › fig/fig-casci/dif-casci(4,4)-rdm.pdf]

Electronic density

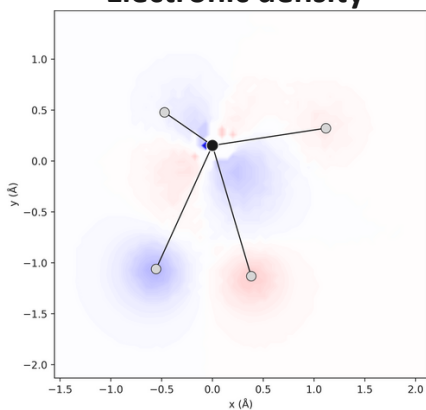

Electrostatic potential

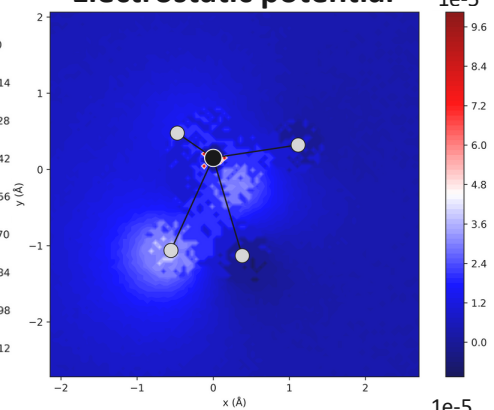 $R = 1.3 \text{ \AA}$ 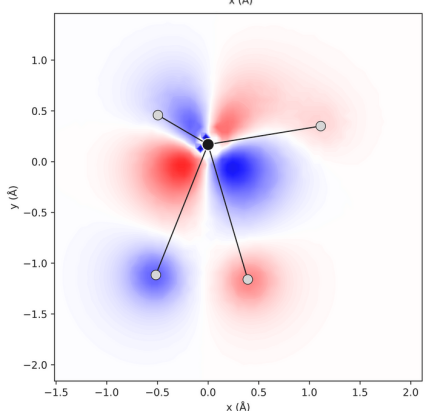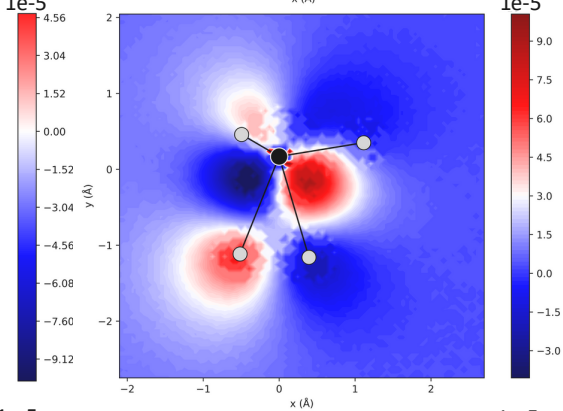 $R = 1.4 \text{ \AA}$ 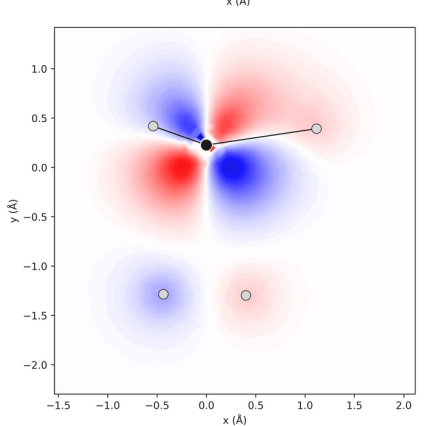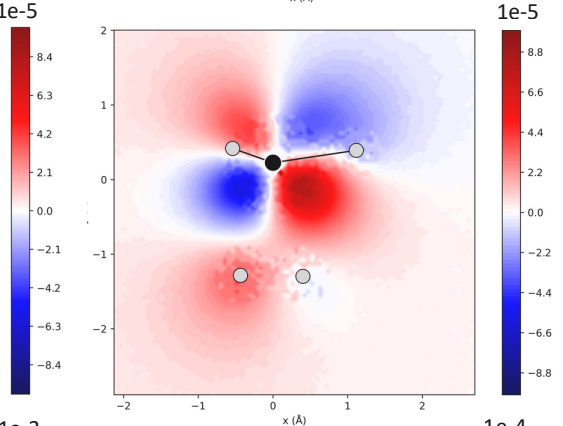 $R = 1.6 \text{ \AA}$ 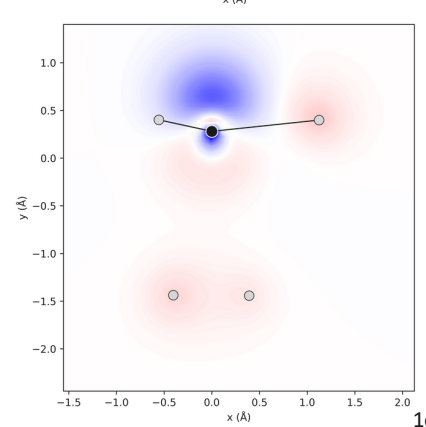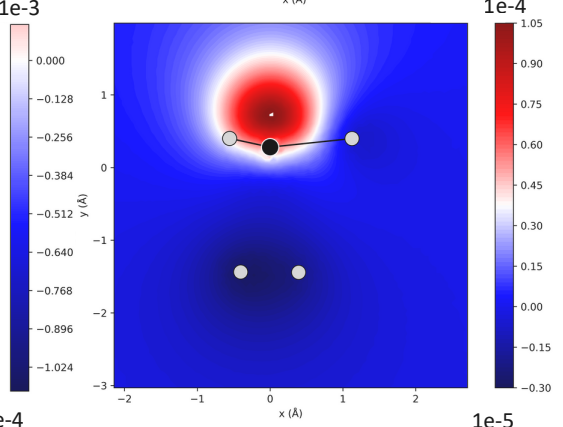 $R = 1.8 \text{ \AA}$ 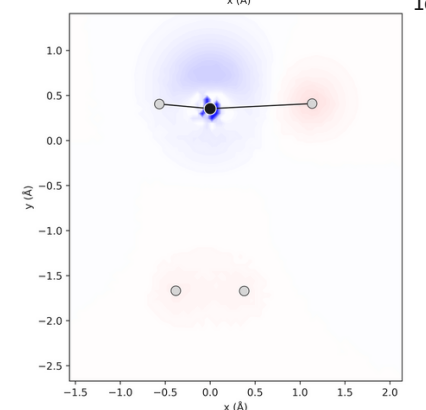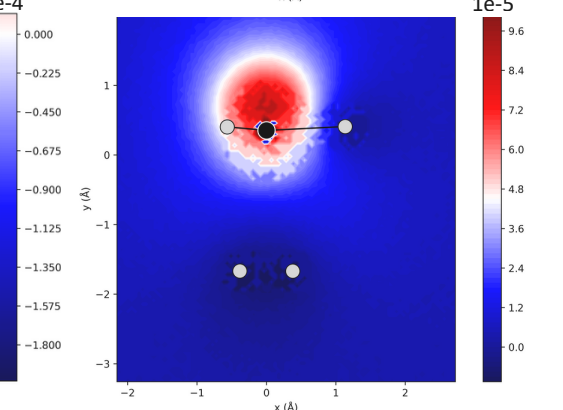 $R = 2.1 \text{ \AA}$

Supplement: Supplementary file 2 — Data S2: jcc70289‐sup‐0002‐Supinfo.zip. [file JCC-47-0-s002.zip › fig/fig-casci/dif-casci(4,4)-vqe-ld.pdf]

**R = 1.3 Å**

**VQE**

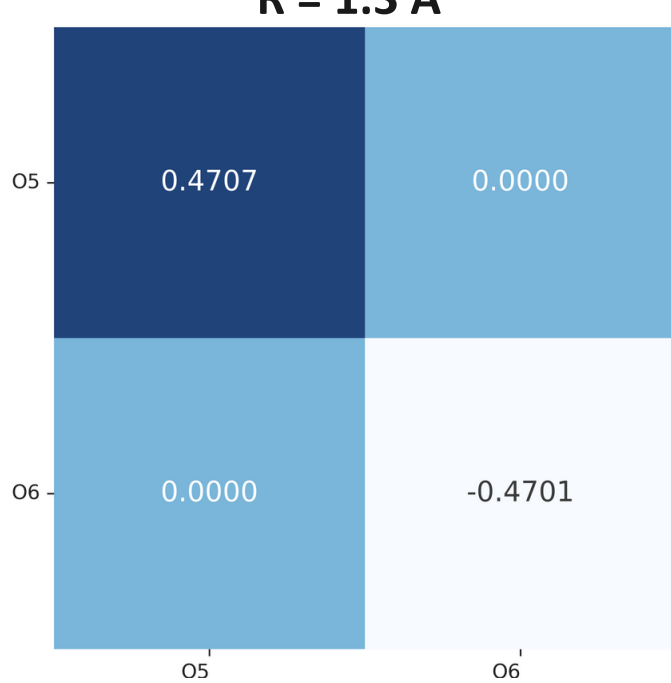

**R = 1.4 Å**

O5

O6

0.2901

0.0000

0.0000

-0.2895

O5

O6

**VQE\***

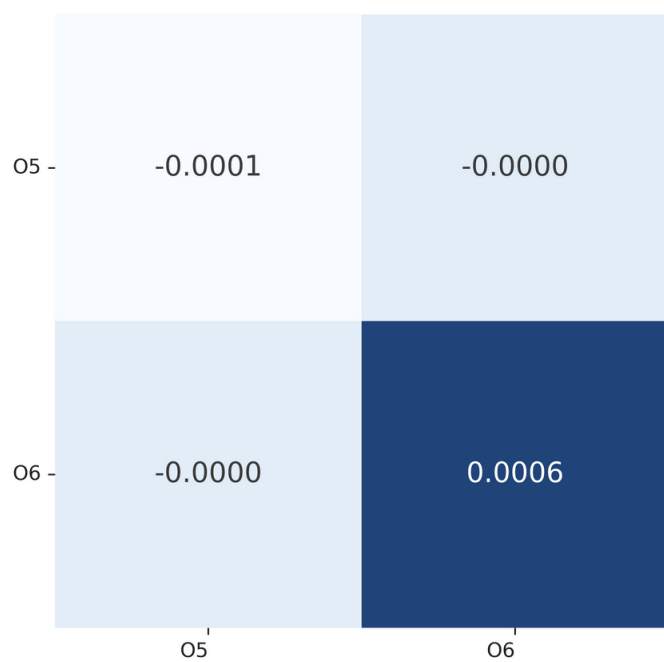

O5

O6

0.0000

0.0000

0.0000

0.0005

O5

O6

**VQE-LD**

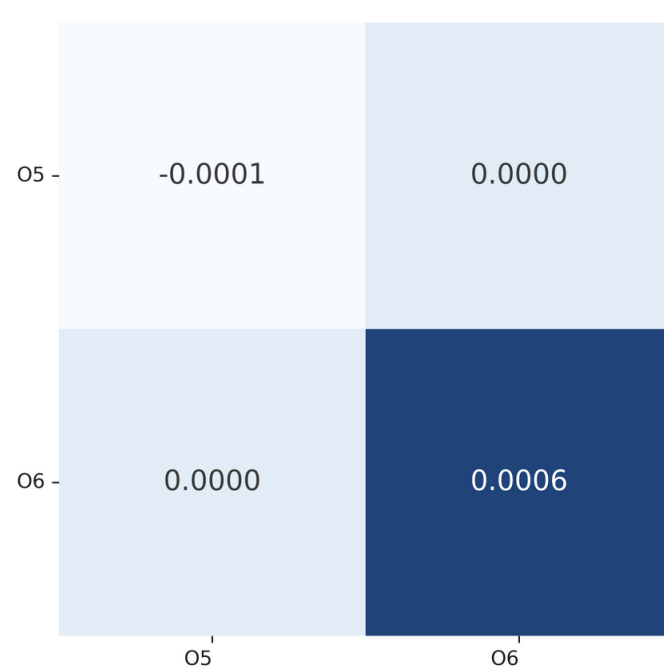

O5

O6

0.0000

0.0000

0.0000

0.0005

O5

O6

Supplement: Supplementary file 2 — Data S2: jcc70289‐sup‐0002‐Supinfo.zip. [file JCC-47-0-s002.zip › fig/fig-casci/rdm1-gf-as2-cas(4,4).pdf]

$R = 1.3 \text{ \AA}$

VQE

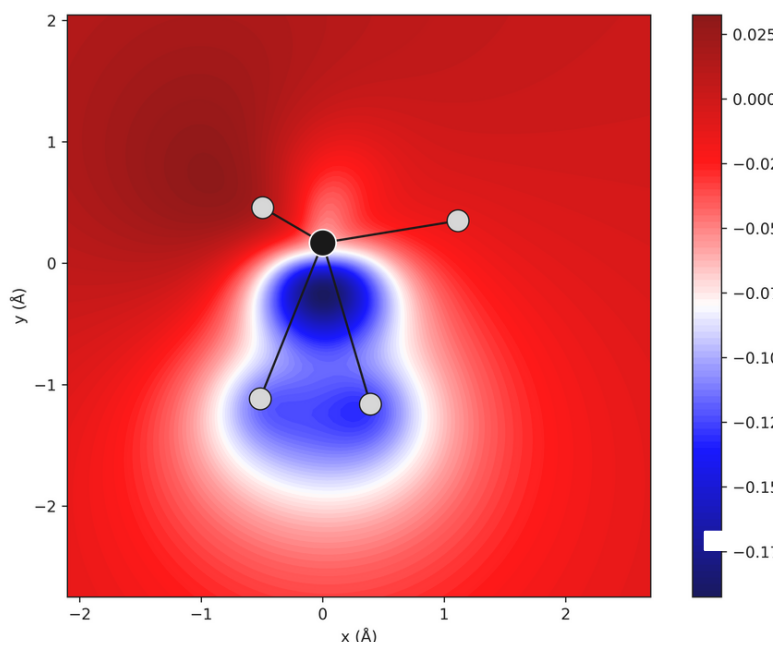

$R = 1.4 \text{ \AA}$

VQE

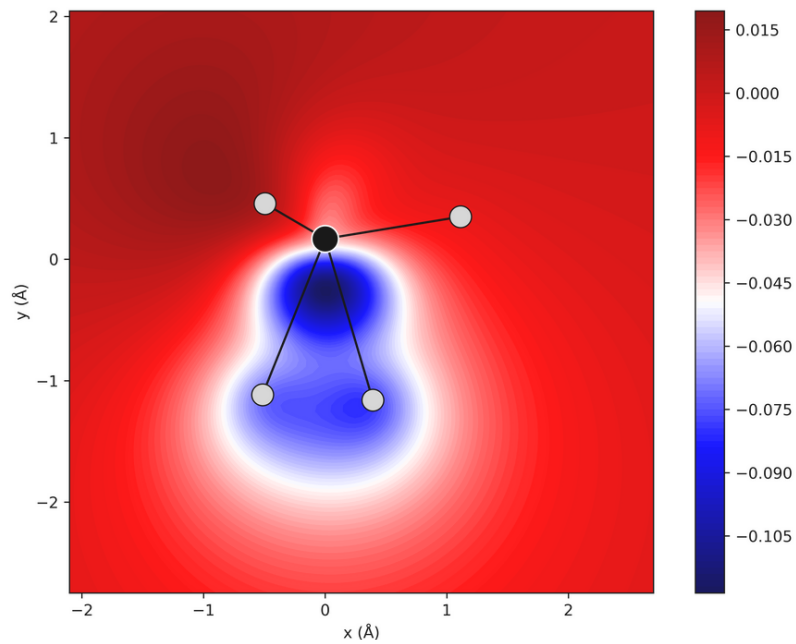

VQE\*

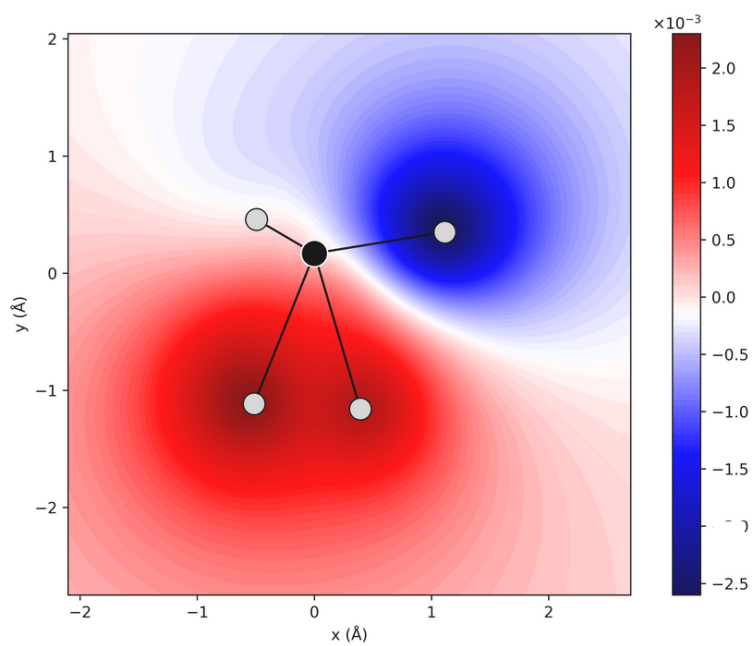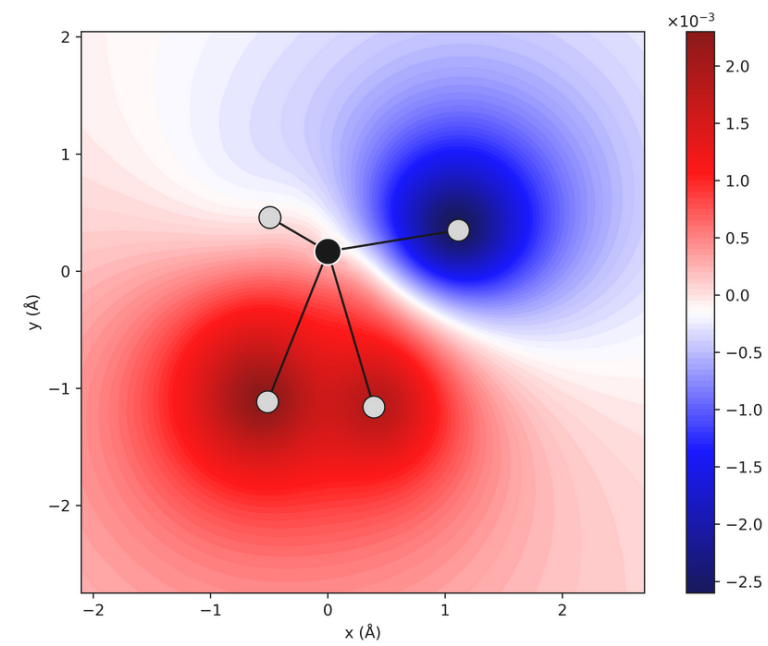

VQE-LD

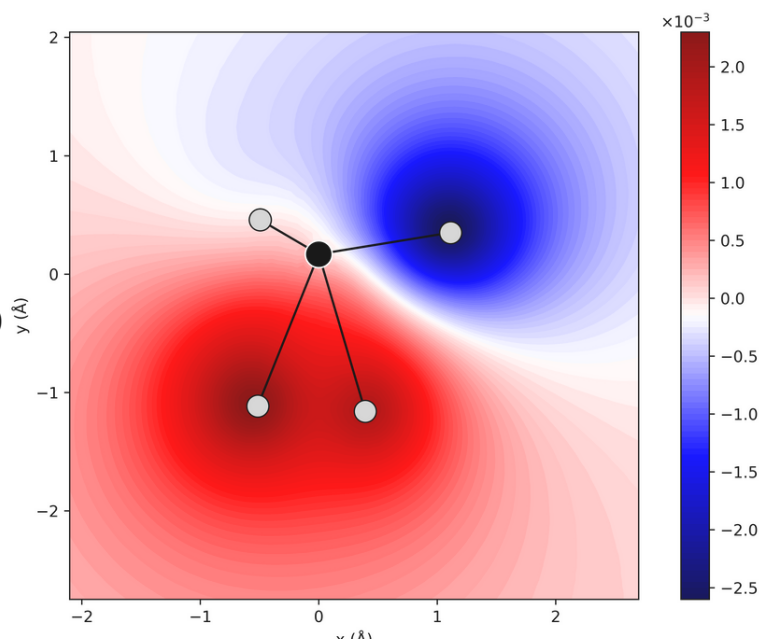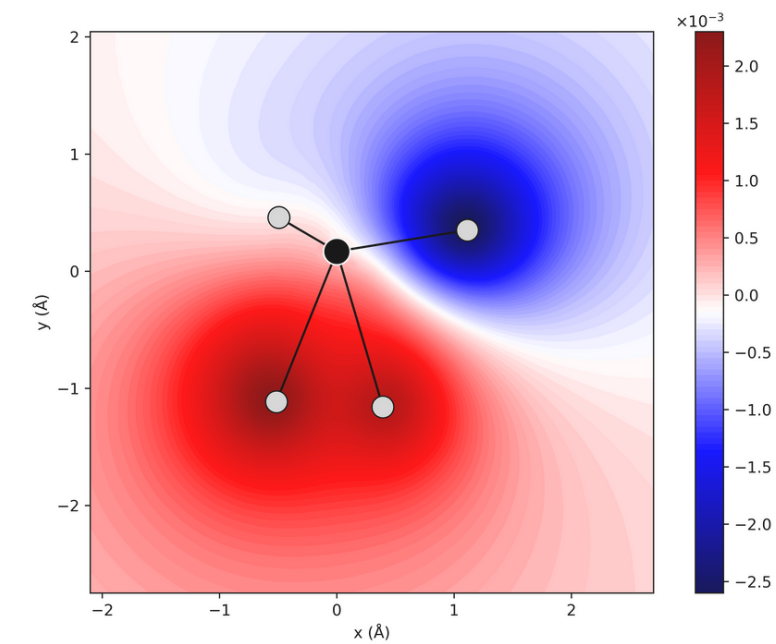

Supplement: Supplementary file 2 — Data S2: jcc70289‐sup‐0002‐Supinfo.zip. [file JCC-47-0-s002.zip › fig/fig-casci/pot-gf-as2-cas(4,4).pdf]
