# Supplementary material for: Improving Energy and Molecular Properties by Convergence of the One‐Particle Reduced Density Matrix in Variational Quantum Eigensolvers (VQE)
Source: J Comput Chem. 2026 Jan 5;47(1):e70289. doi: 10.1002/jcc.70289 (PMC12766880; doi:10.1002/jcc.70289)
Supplement: Supplementary file 2 — Data S2: jcc70289‐sup‐0002‐Supinfo.zip. [file rdm1-as2.pdf › fig/results/rdm1-as2.pdf]

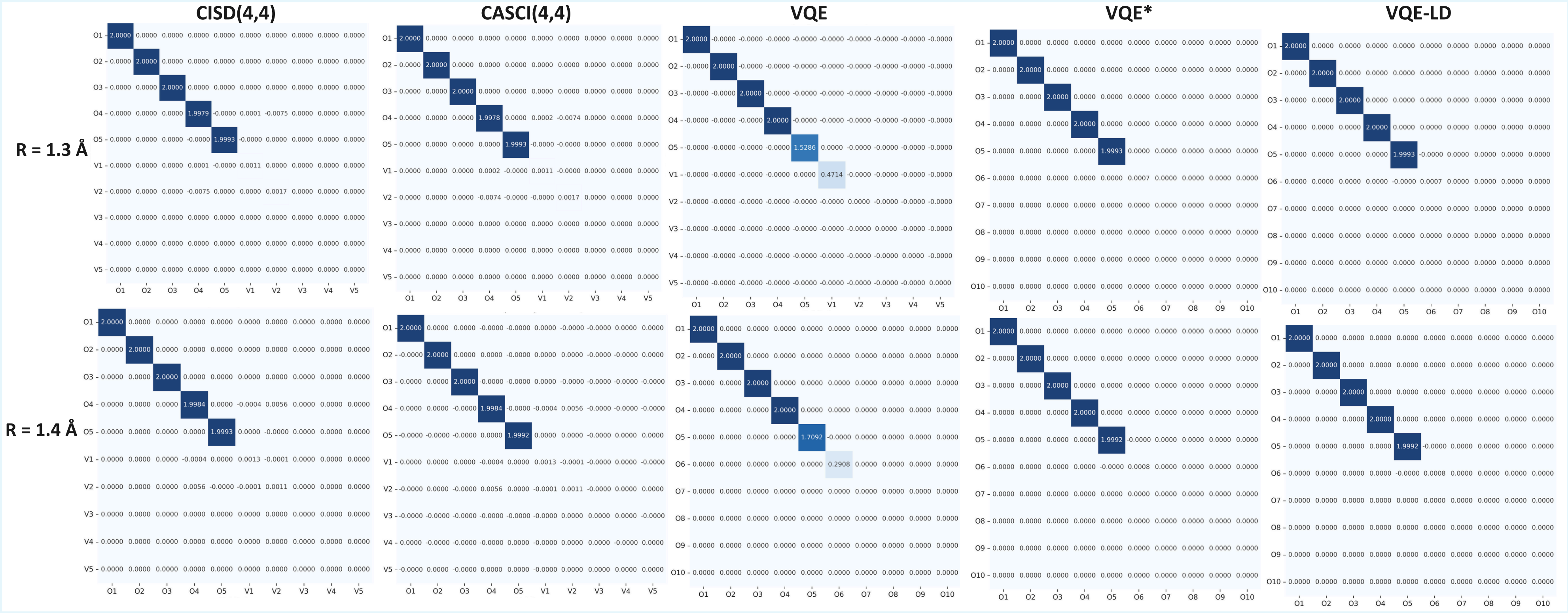
CISD(4,4)
CASCI(4,4)
VQE
VQE*
VQE-LD
R = 1.3 Å
R = 1.4 Å
O1
O2
O3
O4
O5
O6
O7
O8
O9
O10
V1
V2
V3
V4
V5
